# Differential methylation of microRNA encoding genes may contribute to high myopia

**DOI:** 10.3389/fgene.2022.1089784

**Published:** 2023-01-04

**Authors:** Joanna Swierkowska, Sangeetha Vishweswaraiah, Malgorzata Mrugacz, Uppala Radhakrishna, Marzena Gajecka

**Affiliations:** ^1^ Institute of Human Genetics, Polish Academy of Sciences, Poznan, Poland; ^2^ Department of Obstetrics and Gynecology, Oakland University William Beaumont School of Medicine, Royal Oak, MI, United States; ^3^ Department of Ophthalmology and Eye Rehabilitation, Medical University of Bialystok, Bialystok, Poland; ^4^ Chair and Department of Genetics and Pharmaceutical Microbiology, Poznan University of Medical Sciences, Poznan, Poland

**Keywords:** DNA methylation, epigenetic changes, childhood myopia, early-onset high myopia, microRNA target genes, miRNA encoding genes

## Abstract

**Introduction:** High myopia (HM), an eye disorder with a refractive error ≤−6.0 diopters, has multifactorial etiology with environmental and genetic factors involved. Recent studies confirm the impact of alterations in DNA methylation and microRNAs (miRNAs) on myopia. Here, we studied the combined aspects evaluating to the role of methylation of miRNA encoding genes in HM.

**Materials and Methods:** From the genome-wide DNA methylation data of 18 Polish children with HM and 18 matched controls, we retrieved differentially methylated CG dinucleotides localized in miRNA encoding genes. Putative target genes of the highest-ranked miRNAs were obtained from the miRDB and included in overrepresentation analyses in the ConsensusPathDB. Expression of target genes was assessed using the RNA sequencing data of retinal ARPE-19 cell line.

**Results:** We identified differential methylation of CG dinucleotides in promoter regions of *MIR3621*, *MIR34C*, *MIR423* (increased methylation level), and *MIR1178*, *MIRLET7A2*, *MIR885*, *MIR548I3*, *MIR6854*, *MIR675*, *MIRLET7C*, *MIR99A* (decreased methylation level) genes. Several targets of these miRNAs, e.g. *GNAS*, *TRAM1*, *CTNNB1*, *EIF4B*, *TENM3* and *RUNX* were previously associated with myopia/HM/refractive error in Europeans in genome-wide association studies. Overrepresentation analyses of miRNAs’ targets revealed enrichment in pathways/processes related to eye structure/function, such as axon guidance, transcription, focal adhesion, and signaling pathways of TGF-β, insulin, MAPK and EGF-EGFR.

**Conclusion:** Differential methylation of indicated miRNA encoding genes might influence their expression and contribute to HM pathogenesis *via* disrupted regulation of transcription of miRNAs’ target genes. Methylation of genes encoding miRNAs may be a new direction in research on both the mechanisms determining HM and non-invasive indicators in diagnostics.

## 1 Introduction

High myopia (HM) is an eye disorder with a refractive error ≤−6.0 diopters (D) (Young et al., 2007; Flitcroft et al., 2019). It is a complex trait with a multifactorial etiology, including genetic and environmental factors such as near work (Pärssinen et al., 2014), artificial light exposure (Czepita et al., 2004), lack of activity outdoor (Pärssinen et al., 2014; Xiong et al., 2017; Lingham et al., 2021), a higher level of education (Verhoeven et al., 2013) and urbanization (Czepita et al., 2008; Ip et al., 2008; Uzma et al., 2009) or diet with high sugar intake (Galvis et al., 2017). Genetic contribution to refractive error and HM could be monogenic caused by rare mutations but is more often polygenic since a number of genomic regions, candidate genes, and sequence variants involved in HM pathogenesis have been identified (Inamori et al., 2007; Han et al., 2009; Metlapally et al., 2010; Wakazono et al., 2016; Xiao et al., 2016; Wang et al., 2017; Napolitano et al., 2018; Cai et al., 2019; Tideman et al., 2021). Thus far, 27 myopia *loci* have been documented in Online Mendelian Inheritance in Man and we have identified three novel HM *loci* at 7p22.1-7p21.1, 7p12.3-7p11.2, and 12p12.3-12p12.1 in Polish patients (Rydzanicz et al., 2011). Recently, we also recognized two variants in *FLRT3* and *SLC35E2B* genes segregating with the HM phenotype (Swierkowska et al., 2021).

MicroRNAs (miRNAs) constitute an important and highly conserved class of small non-coding RNAs, about 22 nucleotides in length, which play important roles in regulating gene expression (Jiang et al., 2017). MicroRNAs have been reported to be involved in eye development and their abnormal expression or activity were linked to common retinal disorders such as age-related macular degeneration, diabetic retinopathy or retinitis pigmentosa (Xu, 2009; Andreeva and Cooper, 2014; Raghunath and Perumal, 2015; Li et al., 2019; Zuzic et al., 2019). The role of miRNAs, including miR-328 and miR-29a, were also suggested in myopia (Chen et al., 2012; Metlapally et al., 2016; Tkatchenko et al., 2016; Jiang et al., 2017; Mei et al., 2017; Zhang et al., 2017; Kunceviciene et al., 2019, 2021; Tanaka et al., 2019; Xiao et al., 2021; Liu et al., 2022).

Recently, based on the DNA methylation data, we have studied genes overlapping CG dinucleotides that were differentially methylated in Polish children with HM when compared to controls (Vishweswaraiah et al., 2019; Swierkowska et al., 2022). To date, the methylation of miRNA encoding genes has not been studied in myopia or HM and no study was performed in children with HM on the role of miRNA encoding genes. Therefore here, to complement our previously published findings on DNA methylation in Polish children with HM, we assessed the role of methylation in miRNA encoding genes.

## 2 Materials and methods

### 2.1 Patients

A total of 18 Polish Caucasian children with HM and 18 children without HM evaluated as the control group were ascertained at the Department of Paediatric Ophthalmology at Medical University of Bialystok. The participants underwent extensive ophthalmological examinations, including cycloplegic autorefraction, and ocular biometry measurements. The guidelines of the International Myopia Institute, that defines HM as a spherical equivalent refractive error of an eye ≤−6.0 D when ocular accommodation is relaxed (Flitcroft et al., 2019), were followed. Ophthalmic characteristics of HM children and control individuals were described elsewhere (Vishweswaraiah et al., 2019; Swierkowska et al., 2022). The study protocol was approved by the Institutional Review Boards at Poznan University of Medical Sciences in Poland. The written informed consent in accordance with the Declaration of Helsinki was obtained from parents of each child.

### 2.2 Assessment of CG dinucleotides in the miRNA encoding genes

**FIGURE 1:**
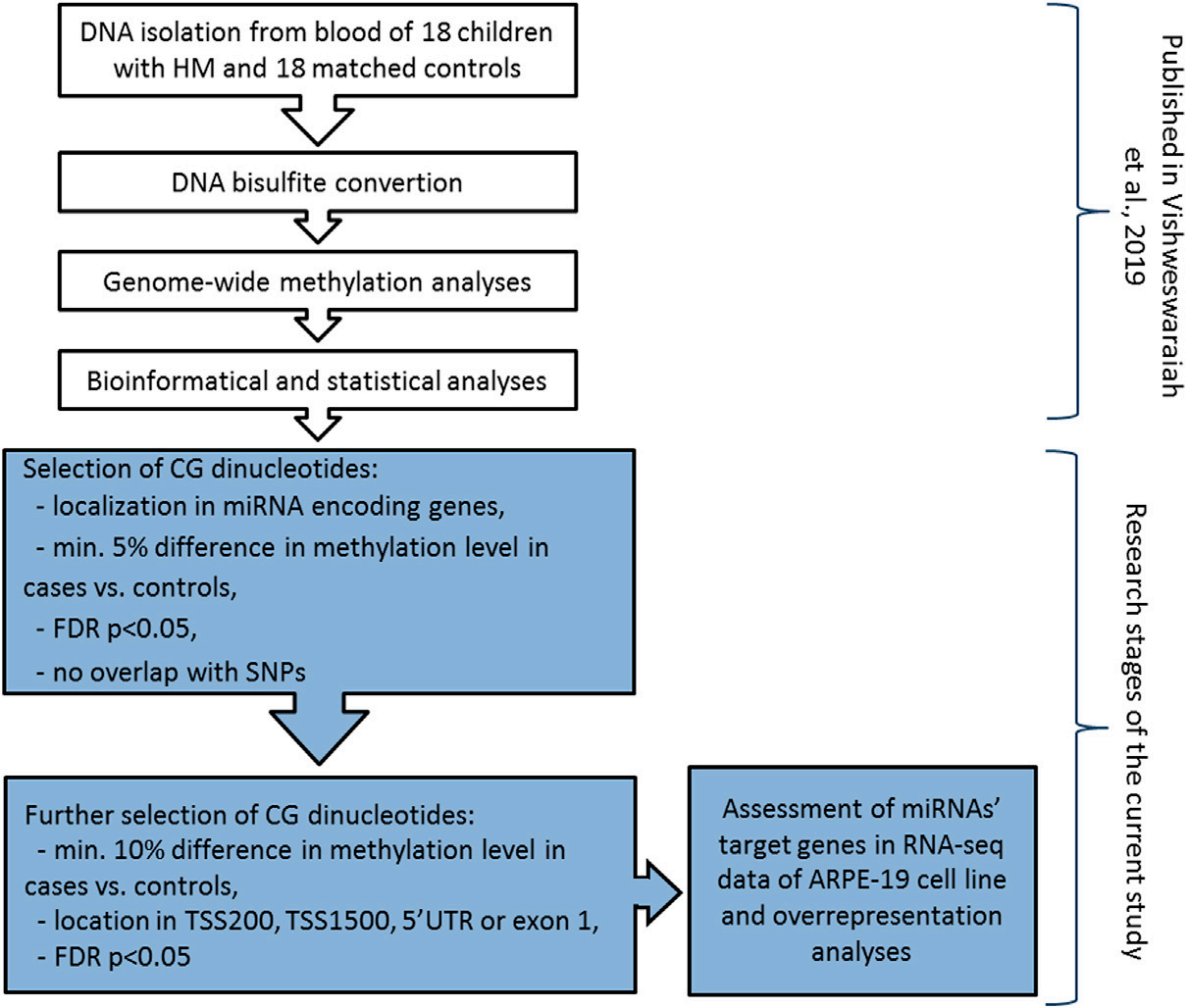
Detailed workflow of miRNA encoding genes methylation analyses in children with HM and matched controls. The research steps marked in white have been already published (Vishweswaraiah et al., 2019). Grey colour indicates the steps performed in the current study. TSS200 means 0–200 bases upstream of the transcriptional start site, TSS1500 means 200–1500 bases upstream of the transcriptional start site, UTR stands for untranslated region, FDR is false discovery rate, and SNP is single nucleotide polymorphism.

DNA methylation analyses was previously performed on genomic DNA extracted from peripheral blood samples using Infinium MethylationEPIC BeadChip arrays (Illumina, Inc., San Diego, CA, United States) covering over 850,000 methylation sites (Vishweswaraiah et al., 2019). The detailed methodology was described elsewhere (Vishweswaraiah et al., 2019). Differentially methylated CG dinucleotides located in miRNA encoding genes (including promoter region) and meeting the following criteria: 1) at least 5% difference in methylation level between HM cases and controls, 2) FDR-corrected *p*-value < 0.05, and 3) no overlap of CG dinucleotides with single nucleotide polymorphisms (SNPs) to avoid potential confounding factors, were retrieved from the previously obtained DNA methylation data. Mean methylation values were calculated for the group of children with HM and the group of children without HM. To avoid any gender-specific methylation bias, CG dinucleotides located on chromosomes X and Y were excluded from the analysis (Kiwerska et al., 2022). Detailed workflow of the study was presented in Figure 1.

Then, to confirm the possibility of altered expression of miRNA encoding genes due to the differential methylation of the promoter region, we applied additional selection criteria. Differential methylation difference of 10% and localization of CG dinucleotides in 5′UTR, exon 1, 0–200 bases upstream of the transcriptional start site (TSS200), or 200–1500 bases upstream of the transcriptional start site (TSS1500) were chosen (Figure 1). Again, the significance of the FDR-corrected *p*-value of < 0.05 was considered. Selected CG dinucleotides localized in the promoter regions of genes encoding miRNAs and those miRNAs were considered as the highest-ranked.

Expression of the highest-ranked miRNA encoding genes and their associations with eye structure and function were assessed in miRBase (http://www.mirbase.org/index.shtml), GeneCards (https://www.genecards.org/), National Center for Biotechnology Information—Gene (NCBI, https://www.ncbi.nlm.nih.gov/), GWAS Catalog (https://www.ebi.ac.uk/gwas/), Mouse Genome Informatics (MGI, http://www.informatics.jax.org/), and available literature data.

### 2.3 Analyses of RNA sequencing data of miRNAs’ putative target genes

Predicted target genes (target score in a range of 50–100) of the highest-ranked miRNAs were obtained from miRDB (http://mirdb.org/) database. The prediction is more reliable with the higher target score. Thus, the expression data of miRNAs’ target genes with target score of ≥90 in the miRDB, was retrieved from available online raw data of RNA sequencing (RNA-seq, GEO: GSE88848) (Samuel et al., 2017) performed on ARPE-19 cell line derived from human retinal pigment epithelium (Figure 1). Transcripts per million (TPM) reads measured at 4 days culture and 4 months culture were available. However, the results of 4 days culture were considered, as the morphology of the ARPE-19 cell line and gene expression may change during the increasing number of passages in the cell culture.

The extensive list of target genes was limited to 5% of the genes with the highest and 5% of the genes with the lowest expression level (>5 TPM) in the ARPE-19 cell line, and assessed in the Human Protein Atlas (proteinatlas.org), GWAS Catalog, GeneCards (https://www.genecards.org/), NCBI, UniProt (https://www.uniprot.org/), MGI databases, and available literature data.

### 2.4 Target genes and pathway overrepresentation analyses

All putative target genes (target score ≥50) of the highest-ranked miRNAs obtained from miRDB were included in the overrepresentation analyses in the Consensus PathDB (http://cpdb.molgen.mpg.de/CPDB) (Figure 1). Following settings were considered: *p*-value cutoff = 0.01 and an overlap of at least five genes from our uploaded gene list, with the Consensus PathDB gene set. Pathways with q-value, *p*-value adjusted for FDR, less than 0.01 were considered.

### 2.5 Statistical analyses

To confirm that the number of the highest-ranked CG dinucleotides was higher than expected by chance, the permutation test of the case-control status of samples and the Student’s t-test were applied. *p*-value < 0.05 was considered.

## 3 Results

### 3.1 Characteristics of the study participants

Children with sporadic HM presented with a refractive error in a range of −6.0 to −15.0 D in at least one eye (mean value of −8.25 D), and axial length ranging from 26.22 to 27.85 mm (mean value of 26.22 mm). Children in the control group had no signs of HM with refractive error ranging from −0.5 D to + 0.5 D (mean −0.25 D), and an axial length between 22.42 mm and 24.11 mm (mean 22.55 mm). All the children were Caucasians, between the ages of 3 and 12 years (mean age of 9.61 years in children with HM and 10.33 years in children without HM). The ratio of boys to girls was comparable between groups. In the HM group 61% and 39% of children and in the control group 56% and 44% were boys or girls, respectively. Detailed characteristics of the patients and controls are presented elsewhere (Vishweswaraiah et al., 2019; Swierkowska et al., 2022).

### 3.2 Identification of differentially methylated CG dinucleotides in miRNA encoding genes

**FIGURE 2:**
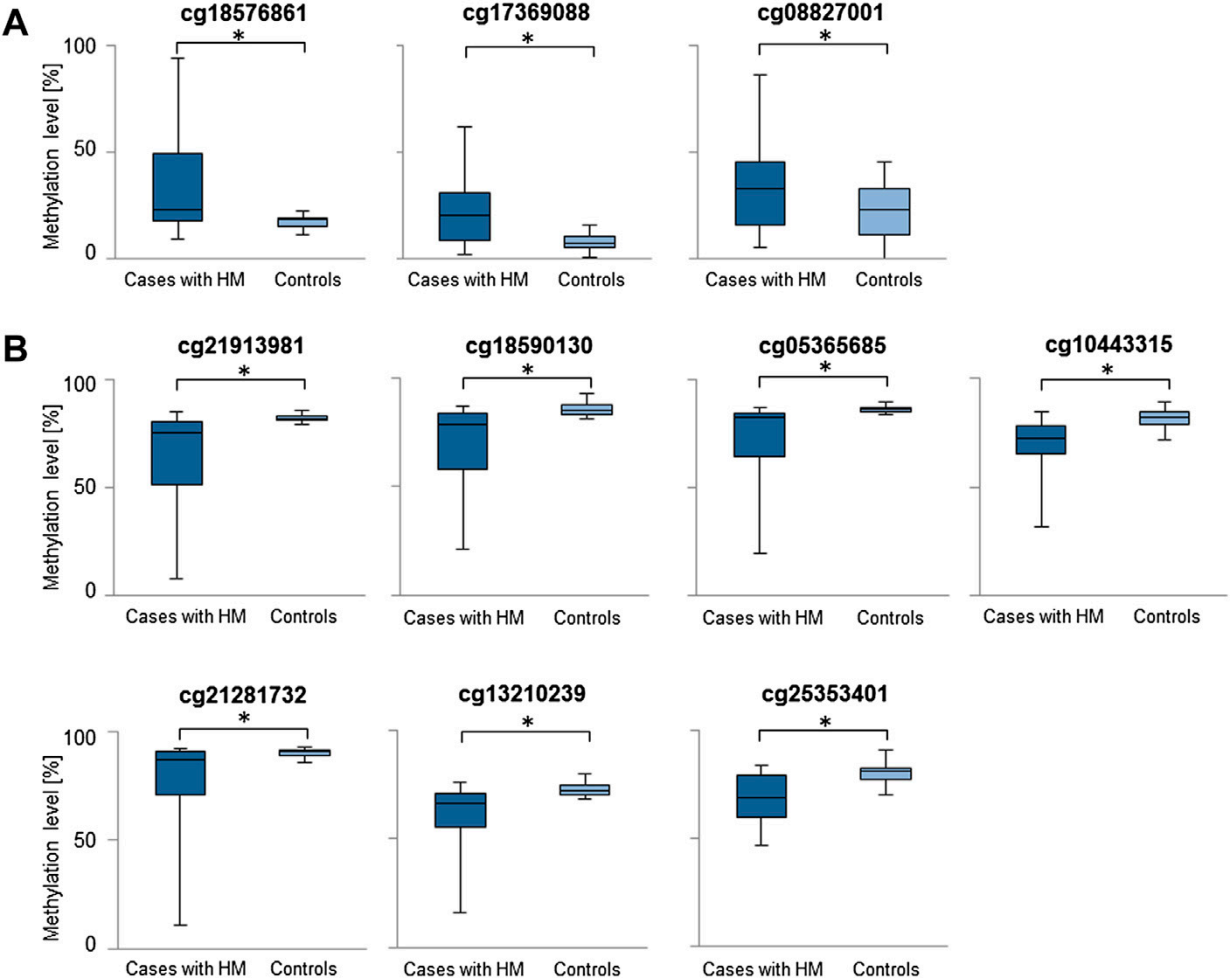
Comparisons of methylation levels of the highest-ranked CG dinucleotides between cases with HM and controls. Presented are CG dinucleotides with at least 10% methylation difference between HM cases and controls and location in miRNA encoding genes promoter regions. Standard deviation is included and asterisk (*) stands for the statistically significant difference in methylation level (FDR-corrected *p*-value < 0.05). **(A)** CG dinucleotides with increased methylation level in HM cases *versus* controls. **(B)** CG dinucleotides with decreased methylation level in HM cases *versus* controls.

Considering the increased methylation level, CG dinucleotides within miRNA encoding genes found with a difference in methylation level in a range of 5.24–16.82% in HM cases vs. controls are listed in Supplementary Table S1. Similarly, considering the decreased methylation level, CG dinucleotides with a difference in methylation level in a range of 5.00–17.38% are provided in Supplementary Table S2. For further analyses we selected differentially methylated CG dinucleotides with at least 10% difference in methylation level, localized in promoter regions of miRNA encoding genes as the highest-ranked CG dinucleotides (Table 1, Figure 2). All the selected CG dinucleotides were found with the FDR-corrected *p*-value < 0.0001 and passed the statistical analyses (*p*-value < 0.05). The highest difference in methylation level between HM cases vs. controls was observed for the cg18576861 in TSS1500 of *MIR3621* (increase of 16.82%) and cg21913981 in TSS200 of *MIR1178* (decrease of 17.38%).

**TABLE 1:** The highest-ranked CG dinucleotides in promoter regions of miRNA encoding genes, with at least 10% difference between HM cases and controls in methylation level.

TargetID	Chromosomal localization	MiRNA encoding gene	Myopia *locus*	*p*-value	FDR *p*-value	Methylation level in HM cases ± SD (%) [range]	Methylation level in controls ± SD (%) [range]	Difference in methylation level (%)	Localization in a gene
*Increased methylation level*									
cg18576861	9q34.3	*MIR3621*		2.29 × 10^−42^	1.98 × 10^−36^	34.09 ± 21.44 [8.9–93.7]	17.27 ± 2.78 [11.5–22.0]	16.82	TSS1500
cg17369088	17q11.2	*MIR423*		5.51 × 10^−41^	4.76 × 10^−35^	21.09 ± 14.95 [2.1–61.4]	8.18 ± 4.29 [0.6–16.0]	12.91	TSS200
cg08827001	11q23.1	*MIR34C*		3.91 × 10^−42^	3.39 × 10^−36^	34.16 ± 21.27 [5.1–86.1]	21.94 ± 13.11 [0–45.0]	12.23	TSS200
*Decreased methylation level*									
cg21913981	12q24.23	*MIR1178*	Nearby the MYP3 (12q21-q23)	5.15 × 10^−30^	4.45 × 10^−24^	64.65 ± 20.06 [7.5–84.8]	82.02 ± 1.67 [79.5–85.6]	−17.38	TSS200
cg18590130	11q24.1	*MIRLET7A2*		2.79 × 10^−30^	2.41 × 10^−24^	69.71 ± 17.62 [21.3–87.3]	85.71 ± 3.31 [81.1–93.1]	−16.00	TSS1500
cg05365685	3p25.3	*MIR885*		2.90 × 10^−23^	2.51 × 10^−17^	72.92 ± 16.6 [19.6–87.0]	86.06 ± 1.55 [84.0–89.9]	−13.14	TSS200
cg10443315	8p23.1	*MIR548I3*	MYP10 (8p23)	2.94 × 10^−19^	2.54 × 10^−13^	69.17 ± 13.19 [31.4–84.7]	82.06 ± 4.35 [71.6–89.1]	−12.89	TSS1500
cg21281732	9q22.33	*MIR6854*		2.90 × 10^−27^	2.51 × 10^−21^	78.26 ± 19.21 [10.6–92.3]	90.43 ± 1.97 [86.0–93.2]	−12.17	TSS1500
cg13210239	11p15.5	*MIR675*		8.46 × 10^−11^	7.31 × 10^−5^	61.51 ± 14.08 [15.8–76.3]	72.56 ± 2.88 [68.7–80.0]	−11.05	TSS1500
cg25353401	21q21.1	*MIRLET7C; MIR99A*		1.57 × 10^−13^	1.36 × 10^−7^	69.54 ± 10.85 [15.8–76.3]	80.46 ± 4.99 [70.7–91.2]	−10.93	TSS1500; TSS1500

### 3.3 Target genes of the highest-ranked miRNAs are expressed in retinal cells

According to the MGI, several the highest-ranked miRNA encoding genes summarized in Table 1, such as *MIR423*, *MIRLET7A2*, *MIRLET7C*, *MIR99A*, have been reported with low expression in a murine eye (GXD: E-GEOD-63810, GXD: E-GEOD-33141, GXD: E-MTAB-6133), but none of them were found to be expressed in ARPE-19 cell line.

For the highest-ranked miRNA encoding genes, we obtained predicted target genes from the miRDB database. Target genes of the highest-ranked miRNAs encoding genes with increased and decreased methylation level are presented in Supplementary Tables S3, S4, respectively.

To narrow the list of miRNAs’ target genes that are probably related to HM, target genes with a target score ≥90 in miRDB were also assessed for expression level in the RNA-seq data (Supplementary Tables S5, S6). Target genes with the highest and the lowest expression level, the role in the function or structure of the eye or localization in myopia *loci* were characterized and summarized in Supplementary Table S7.

### 3.4 Pathways and molecular processes related to myopia/eye disorders

The analyses revealed enrichment in biological pathways related to eye structure and function (Supplementary Tables S8, S9). In our results the most common significant pathways/processes that could be related to myopia were axon guidance, transcription, TGF-β signaling pathway, insulin signaling, focal adhesion, MAPK signaling pathway, and EGF-EGFR signaling pathway.

## 4 Discussion

To compliment the already performed analyses on differentially methylated genes in HM in Polish children (Vishweswaraiah et al., 2019; Swierkowska et al., 2022), we focused here on CG dinucleotides located in miRNA encoding genes. We report significant increase in methylation level of CG dinucleotides located in the promoter regions of *MIR3621*, *MIR34C*, *MIR423*, and significant decrease in the promoter regions of *MIR1178*, *MIRLET7A2*, *MIR885*, *MIR548I3*, *MIR6854*, *MIR675*, *MIRLET7C*, and *MIR99A* genes.

From the indicated miRNA encoding genes, two encoded miRNAs were previously reported in myopia studies. The first one, miR-885, was identified in exosomes in aqueous humor of myopic patients undergoing cataract surgery (Chen et al., 2019). Second one, let-7a, had confirmed significant differential expression in mice sclera of myopic form-deprived eyes when compared to control eyes, supporting the involvement of miRNAs in eye growth regulation (Metlapally et al., 2016). The expression of the let-7a-5p was also significantly downregulated in lens epithelium samples of patients with senile cataract, and a significant difference in expression between nuclear and anterior subcapsular cataracts has been found for the let-7a-5p (Kim et al., 2021). This miRNA was also significantly downregulated in rat glaucomatous retina when compared with controls (Jayaram et al., 2015). Moreover, functional analyses indicated that let-7a could be crucial for RPE differentiation and maintenance of the epithelial phenotype of the cells (Shahriari et al., 2020). Furthermore, upregulation of let-7a-5p represses TGF-β2-induced proliferation, migration, invasion and epithelial-mesenchymal transition in human lens epithelial cells (Liu and Jiang, 2020).

The second indicated miRNA let-7, let-7c-3p, was downregulated in anterior lens capsules of age-related cataract patients aged over 65 years relative to the patients under the age of 65 years (Li et al., 2020). Also, let-7c-3p inhibited autophagy by targeting ATG3 in human lens epithelial cells (Li et al., 2020). Furthermore, increased expression of let-7c was detected in fetal sclera when compared to sclera of the adults (Metlapally et al., 2013), and was only expressed in plasma of patients with age-related macular degeneration and not in plasma of controls (Ertekin et al., 2014). Both let-7a-5p and let-7c-5p were significantly upregulated in aqueous humor samples of normal-tension glaucoma patients when compared to the control group (Seong et al., 2021).

Previously, another miRNA, miR-34c, was found to affect the growth of trigeminal sensory neurons and the repair of diabetic corneal nerve endings in diabetic corneal neuropathy (Hu et al., 2019). Functional experiments demonstrated that miR-34c could reverse the oncogenic function of lncRNA DANCR (differentiation antagonizing non-protein coding RNA) in retinoblastoma tumorigenesis (Wang et al., 2018), and that miR-34c acts as tumor suppressor in uveal melanoma cell proliferation and migration through the down regulation of multiple targets (Dong and Lou, 2012).

A study has shown that miR-423-5p is highly expressed in the vitreous of eyes with proliferative diabetic retinopathy (Hirota et al., 2015), and in aqueous humor of patients with intraocular tuberculosis (Chadalawada et al., 2022).

Moreover, miR-675 regulates the expression of the *CRYAA* gene, that its dysfunction causes cataract as it contributes to the transparency and refractive index of the lens, by targeting the binding site within the 3′UTR (Liu et al., 2018). LncRNA H19, a precursor of miR-675, is significantly up-regulated in the nuclear age-related cataract lenses, and its reduction inhibits miR-675 expression (Liu et al., 2018). Therefore, in general literature supports our results about the possible involvement of miR-885, miR-34c, miR-423, let-7a-2, miR-675, and miR-let7c in HM in children.

Several putative target genes of the highest-ranked miRNAs were previously associated with eye diseases in genome-wide association studies (GWAS). Also, a *GNAS* gene was associated with HM (Tideman et al., 2021), *TRAM1*, *CTNNB1*, *TENM3* and *RUNX* with myopia and/or refractive error (Han et al., 2020; Hysi et al., 2020; Xue et al., 2022), and *EIF4B* with low myopia/hyperopia (Tideman et al., 2021) in Europeans. Moreover, genes *CBX3*, *NAP1L1*, *EIF4B*, *PLS3*, *MRFAP1*, *GPATCH8*, *TENM3*, *BAZ2A*, *AMMECR1*, *JAZF1*, *PIM3*, *DNA2*, *GTPBP1*, *ITGB3*, *DDI2* are localized in myopia/HM *loci*. Furthermore, mutations in *DCBLD2*, *GNAS*, *CTNNB1*, *BZW1*, *ACTN4*, *LIMCH1*, *FKBP1B, VAV2, ENKD1, ITGB3* cause abnormal murine eye phenotype (MGI:1920629, MGI:95777, MGI:88276, MGI:1914132, MGI:1890773, MGI:1924819, MGI:1336205, MGI:102718, MGI:2142593, MGI:96612). Abnormal transcription of the listed target genes might have a crucial role in HM pathogenesis.

Overrepresentation analyses of miRNAs’ targets revealed enrichment in biological pathways/processes related to eye structure and function, such as axon guidance, transcription, focal adhesion, insulin signaling, and signaling pathways of TGF-β, MAPK, and EGF-EGFR. Similarly, Mei et al. also revealed significant enrichment of target genes of differentially expressed miRNAs in murine eyes with form-deprivation myopia in such processes as regulation of transcription, axon guidance, and TGF-β signaling pathway (Mei et al., 2017). Flitcroft et al. (2018) as well identified several biological processes already implicated in refractive error development, including focal adhesion, axon guidance, and extracellular matrix remodeling (Flitcroft et al., 2018). As for the insulin signaling, intravitreally injected insulin promoted axial eye growth in chicks (Penha et al., 2012), and Liu et al. (2015) showed significant association of SNPs in the *INS-IGF2* region, and the *INSR* (insulin receptor) gene with HM (Liu et al., 2015). Summarizing, indicated molecular pathways/processes could be related to HM in the studied children.

The study limitation is the assessment of DNA methylation in blood samples instead of eye tissue. Retinal samples could not be obtained from the ascertained children. However, other studies were also performed on patients’ blood instead of eye tissue, making all results comparable (Hsi et al., 2019). Still, our previously published results in aspects of methylation and the current data were obtained and compiled for the same patients and controls, which effectively brings it all together. Further analyses of a larger cohort, including assessments of children’s lifestyle and other environmental factors data, are needed. Moreover, we did not examine whether the children’s mothers were exposed to pollution, heavy metals, smoking, or nutritional habits during pregnancy, as these are well-known factors affecting methylation in children (Alvarado-Cruz et al., 2018). Furthermore, the ARPE-19 cell line is not fully representative of primary tissue data and epigenetic modifications and expression may be altered within ARPE-19 cultured cells from the primary human state. However, functional studies are necessary to be performed to confirm or deny the findings of this computational study and *in silico* obtained results.

To conclude, differential methylation of CG dinucleotides in promoters of the miRNA encoding genes, *MIR3621*, *MIR34C*, *MIR423*, *MIR1178*, *MIRLET7A2*, *MIR885*, *MIR548I3*, *MIR6854*, *MIR675*, *MIRLET7C*, *MIR99A*, might influence their expression. Therefore, these findings may contribute to HM pathogenesis *via* the disrupted regulation of transcription of miRNAs’ target genes and biological pathways crucial for eye development and function. Further studies of methylation may shed light on the molecular mechanisms underlying both genetic and environmental phenotypic effects. Moreover, the identified features concerning specific CG dinucleotides in miRNA encoding genes could be promising for developing non-invasive biomarkers of HM, detectable in blood.

## Data Availability

The original contributions presented in the study are included in the article/Supplementary Material, further inquiries can be directed to the corresponding author.
