# Supplementary material for: Differential methylation of microRNA encoding genes may contribute to high myopia
Source: Front Genet. 2023 Jan 4;13:1089784. doi: 10.3389/fgene.2022.1089784 (PMC9847511; doi:10.3389/fgene.2022.1089784)
Supplement: Supplementary file 2 [file Table1.DOCX]

**Supplementary Table 1. Increased methylation levels: the highest-ranked CG dinucleotides in promoter regions and gene body of miRNA encoding genes, with at least 5% difference between HM cases and controls in methylation level**

| **TargetID** | **Chromosome** | **miRNA encoding gene** | **p-value** | **FDR p-value** | **Methylation level in HM cases [%]*** | **Methylation level in controls [%]*** | **Difference in methylation level [%]** | **Localization in gene** |
| --- | --- | --- | --- | --- | --- | --- | --- | --- |
| cg18576861 | 9 | *MIR3621* | 2.29x10^-42^ | 1.98x10^-36^ | 34.09 | 17.27 | 16.82 | TSS1500 |
| cg17369088 | 17 | *MIR423* | 5.51x10^-41^ | 4.76x10^-35^ | 21.09 | 8.18 | 12.91 | TSS200 |
| cg11926525 | 15 | *MIR548H4* | 2.43x10^-40^ | 2.10x10^-34^ | 14.91 | 2.39 | 12.52 | gene body |
| cg08827001 | 11 | *MIR34C* | 3.91x10^-42^ | 3.39x10^-36^ | 34.16 | 21.94 | 12.23 | TSS200 |
| cg13767001 | 13 | *MIR759* | 9.49x10^-39^ | 8.21x10^-33^ | 14.84 | 7.21 | 7.63 | TSS1500 |
| cg14738611 | 4 | *MIR4453* | 1.80x10^-9^ | 1.56x10^-3^ | 15.86 | 9.64 | 6.22 | TSS1500 |
| cg03902122 | 5 | *MIR449C* | 6.92x10^-39^ | 5.98x10^-33^ | 14.38 | 8.41 | 5.97 | TSS200 |
| cg27569588 | 14 | *MIR345* | 2.29x10^-13^ | 1.98x10^-7^ | 9.78 | 3.82 | 5.95 | TSS1500 |
| cg19767580 | 16 | *MIR3181* | 1.28x10^-12^ | 1.11x10^-6^ | 7.38 | 1.76 | 5.62 | TSS200 |
| cg06606386 | 19 | *MIR27A;MIR24-2;MIR23A* | 3.30x10^-9^ | 2.86x10^-3^ | 24.27 | 18.67 | 5.60 | TSS200;TSS1500;gene body |
| cg01629329 | 20 | *MIR1292* | 2.62x10^-8^ | 2.26x10^-2^ | 13.47 | 7.92 | 5.55 | TSS200 |
| cg26665229 | 8 | *MIR2052HG* | 4.88x10^-8^ | 4.22x10^-2^ | 18.84 | 13.30 | 5.54 | gene body |
| cg26945813 | 10 | *MIR1915* | 2.02x10^-11^ | 1.75x10^-5^ | 9.60 | 4.30 | 5.30 | TSS1500 |
| cg06162516 | 12 | *MIRLET7I* | 2.50x10^-10^ | 2.16x10^-4^ | 16.12 | 10.88 | 5.24 | TSS1500 |

FDR - false discovery rate, TSS1500 - 200-1500 bases upstream of the transcriptional start site, TSS200 - 0-200 bases upstream of the transcriptional start site,

* Methylation levels in children are presented as mean values
