# Supplementary material for: Differential methylation of microRNA encoding genes may contribute to high myopia
Source: Front Genet. 2023 Jan 4;13:1089784. doi: 10.3389/fgene.2022.1089784 (PMC9847511; doi:10.3389/fgene.2022.1089784)
Supplement: Supplementary file 3 [file Table8.DOCX]

**Supplementary Table 8. Identified signaling pathways and biological processes in overrepresentation analyses of target genes of the highest-ranked miRNAs - increased methylation level**

| **Pathway name** | **Set size** | **Candidates contained** | **p-value** | **q-value** | **Pathway source** |
| --- | --- | --- | --- | --- | --- |
| ***MIR34C*** | | | | | |
| **miR-34c-5p** | | | | | |
| Cushing syndrome - Homo sapiens (human) | 154 | 22 (14.3%) | 5.65e-07 | 0.000344 | KEGG |
| Notch signaling pathway | 54 | 12 (22.6%) | 1.66e-06 | 0.000506 | PID |
| Regulation of RUNX1 Expression and Activity | 18 | 7 (41.2%) | 3.22e-06 | 0.000537 | Reactome |
| Axon guidance | 358 | 35 (9.8%) | 3.53e-06 | 0.000537 | Reactome |
| Notch Signaling Pathway | 61 | 12 (19.7%) | 7.95e-06 | 0.000968 | Wikipathways |
| Aldosterone synthesis and secretion - Homo sapiens (human) | 96 | 15 (15.6%) | 1.19e-05 | 0.00121 | KEGG |
| Developmental Biology | 620 | 49 (7.9%) | 1.86e-05 | 0.00162 | Reactome |
| Parathyroid hormone synthesis, secretion and action - Homo sapiens (human) | 106 | 15 (14.2%) | 3.98e-05 | 0.00287 | KEGG |
| Phospholipase D signaling pathway - Homo sapiens (human) | 146 | 18 (12.4%) | 4.24e-05 | 0.00287 | KEGG |
| Cortisol synthesis and secretion - Homo sapiens (human) | 64 | 11 (17.2%) | 7.07e-05 | 0.0043 | KEGG |
| Oxytocin signaling pathway - Homo sapiens (human) | 152 | 18 (11.8%) | 7.92e-05 | 0.00439 | KEGG |
| Melanogenesis - Homo sapiens (human) | 101 | 14 (13.9%) | 9,00E-05 | 0.00442 | KEGG |
| L1CAM interactions | 103 | 14 (13.7%) | 0.0001 | 0.00442 | Reactome |
| Canonical and Non-canonical Notch signaling | 27 | 7 (25.9%) | 0.000102 | 0.00442 | Wikipathways |
| miR-targeted genes in lymphocytes - TarBase | 489 | 39 (8.0%) | 0.000115 | 0.00468 | Wikipathways |
| Receptor-ligand binding initiates the second proteolytic cleavage of Notch receptor | 13 | 5 (38.5%) | 0.000132 | 0.00498 | Reactome |
| Notch | 59 | 10 (17.2%) | 0.000146 | 0.00498 | NetPath |
| Diseases of signal transduction | 248 | 24 (9.7%) | 0.000147 | 0.00498 | Reactome |
| Regulation of insulin secretion | 70 | 11 (15.7%) | 0.000163 | 0.00516 | Reactome |
| Kit receptor signaling pathway | 59 | 10 (16.9%) | 0.000169 | 0.00516 | Wikipathways |
| EPHB forward signaling | 39 | 8 (20.5%) | 0.000193 | 0.00559 | PID |
| miR-targeted genes in muscle cell - TarBase | 400 | 33 (8.2%) | 0.000207 | 0.00559 | Wikipathways |
| Adherens junction - Homo sapiens (human) | 72 | 11 (15.3%) | 0.000211 | 0.00559 | KEGG |
| Nongenotropic Androgen signaling | 31 | 7 (22.6%) | 0.00026 | 0.00659 | PID |
| Cholinergic synapse - Homo sapiens (human) | 112 | 14 (12.5%) | 0.000275 | 0.00669 | KEGG |
| LPA receptor mediated events | 64 | 10 (15.9%) | 0.000296 | 0.00693 | PID |
| Intracellular signaling by second messengers | 245 | 23 (9.4%) | 0.000313 | 0.00706 | Reactome |
| Amplification and Expansion of Oncogenic Pathways as Metastatic Traits | 16 | 5 (31.2%) | 0.000402 | 0.00874 | Wikipathways |
| VEGFA-VEGFR2 Signaling Pathway | 236 | 22 (9.3%) | 0.000462 | 0.00929 | Wikipathways |
| Sphingolipid signaling pathway - Homo sapiens (human) | 118 | 14 (11.9%) | 0.000473 | 0.00929 | KEGG |
| Rap1 signaling pathway - Homo sapiens (human) | 206 | 20 (9.7%) | 0.000493 | 0.00929 | KEGG |
| G Protein Signaling Pathways | 92 | 12 (13.0%) | 0.0005 | 0.00929 | Wikipathways |
| Disease | 510 | 38 (7.5%) | 0.000504 | 0.00929 | Reactome |
| Amphetamine addiction - Homo sapiens (human) | 68 | 10 (14.7%) | 0.000557 | 0.00969 | KEGG |
| Signaling events mediated by VEGFR1 and VEGFR2 | 68 | 10 (14.7%) | 0.000557 | 0.00969 | PID |
| **miR-34c-5p and miR-34c-3p** | | | | | |
| Cushing syndrome - Homo sapiens (human) | 154 | 29 (18.8%) | 3.94e-08 | 3.35e-05 | KEGG |
| VEGFA-VEGFR2 Signaling Pathway | 236 | 36 (15.3%) | 2.65e-07 | 0.000112 | Wikipathways |
| Signaling events mediated by Hepatocyte Growth Factor Receptor (c-Met) | 82 | 18 (22.2%) | 1.24e-06 | 0.000351 | PID |
| miR-targeted genes in muscle cell - TarBase | 400 | 48 (12.0%) | 4.29e-06 | 0.000911 | Wikipathways |
| Notch signaling pathway | 54 | 13 (24.5%) | 1.19e-05 | 0.00158 | PID |
| miR-targeted genes in lymphocytes - TarBase | 489 | 54 (11.0%) | 1.29e-05 | 0.00158 | Wikipathways |
| Regulation of Microtubule Cytoskeleton | 46 | 12 (26.1%) | 1.31e-05 | 0.00158 | Wikipathways |
| Axon guidance | 358 | 42 (11.8%) | 2.74e-05 | 0.00258 | Reactome |
| Melanogenesis - Homo sapiens (human) | 101 | 18 (17.8%) | 3.22e-05 | 0.00258 | KEGG |
| Intracellular signaling by second messengers | 245 | 32 (13.1%) | 3.29e-05 | 0.00258 | Reactome |
| Regulation of RUNX1 Expression and Activity | 18 | 7 (41.2%) | 3.34e-05 | 0.00258 | Reactome |
| Oxytocin signaling pathway - Homo sapiens (human) | 152 | 23 (15.1%) | 4.3e-05 | 0.00304 | KEGG |
| Aldosterone synthesis and secretion - Homo sapiens (human) | 96 | 17 (17.7%) | 5.76e-05 | 0.00332 | KEGG |
| Notch Signaling Pathway | 61 | 13 (21.3%) | 5.91e-05 | 0.00332 | Wikipathways |
| Developmental Biology | 620 | 62 (10.0%) | 6.05e-05 | 0.00332 | Reactome |
| Parathyroid hormone synthesis, secretion and action - Homo sapiens (human) | 106 | 18 (17.0%) | 6.26e-05 | 0.00332 | KEGG |
| Autophagy - animal - Homo sapiens (human) | 128 | 20 (15.6%) | 8.45e-05 | 0.00402 | KEGG |
| Energy Metabolism | 47 | 11 (23.4%) | 8.85e-05 | 0.00402 | Wikipathways |
| Adherens junction - Homo sapiens (human) | 72 | 14 (19.4%) | 9.01e-05 | 0.00402 | KEGG |
| Cortisol synthesis and secretion - Homo sapiens (human) | 64 | 13 (20.3%) | 0.0001 | 0.00419 | KEGG |
| signaling pathway from g-protein families | 26 | 8 (30.8%) | 0.000104 | 0.00419 | BioCarta |
| Dopaminergic synapse - Homo sapiens (human) | 131 | 20 (15.3%) | 0.000118 | 0.00436 | KEGG |
| Renin secretion - Homo sapiens (human) | 65 | 13 (20.0%) | 0.000118 | 0.00436 | KEGG |
| Integration of energy metabolism | 94 | 16 (17.0%) | 0.000153 | 0.00537 | Reactome |
| Insulin secretion - Homo sapiens (human) | 85 | 15 (17.6%) | 0.000161 | 0.00537 | KEGG |
| Myometrial Relaxation and Contraction Pathways | 155 | 22 (14.2%) | 0.000164 | 0.00537 | Wikipathways |
| Amphetamine addiction - Homo sapiens (human) | 68 | 13 (19.1%) | 0.000191 | 0.006 | KEGG |
| Apelin signaling pathway - Homo sapiens (human) | 137 | 20 (14.6%) | 0.000219 | 0.00658 | KEGG |
| Neuronal System | 368 | 40 (10.9%) | 0.000225 | 0.00658 | Reactome |
| Regulation of insulin secretion | 70 | 13 (18.6%) | 0.000258 | 0.0073 | Reactome |
| ***MIR423*** | | | | | |
| **miR-423-5p** | | | | | |
| Axon guidance | 358 | 31 (8.7%) | 6.2e-06 | 0.00283 | Reactome |
| VEGFA-VEGFR2 Pathway | 92 | 13 (14.1%) | 2.59e-05 | 0.00381 | Reactome |
| Wnt | 69 | 11 (16.2%) | 2.99e-05 | 0.00381 | NetPath |
| Regulation of Actin Cytoskeleton | 151 | 17 (11.3%) | 3.33e-05 | 0.00381 | Wikipathways |
| Signaling by VEGF | 100 | 13 (13.0%) | 6.33e-05 | 0.0051 | Reactome |
| Developmental Biology | 620 | 42 (6.8%) | 6.69e-05 | 0.0051 | Reactome |
| Regulation of actin cytoskeleton - Homo sapiens (human) | 213 | 20 (9.4%) | 9.57e-05 | 0.00625 | KEGG |
| Senescence and Autophagy in Cancer | 106 | 13 (12.3%) | 0.000116 | 0.00664 | Wikipathways |
| Phase 0 - rapid depolarisation | 44 | 8 (18.2%) | 0.000158 | 0.00801 | Reactome |
| Regulation of Microtubule Cytoskeleton | 46 | 8 (17.4%) | 0.000218 | 0.00996 | Wikipathways |
| **miR-423-3p** | | | | | |
| Pathways in cancer - Homo sapiens (human) | 526 | 5 (1.0%) | 0.00158 | 0.00758 | KEGG |
| **miR-423-5p and miR-423-3p** | | | | | |
| Regulation of Actin Cytoskeleton | 151 | 20 (13.2%) | 1.01e-06 | 0.000293 | Wikipathways |
| VEGFA-VEGFR2 Pathway | 92 | 15 (16.3%) | 1.66e-06 | 0.000293 | Reactome |
| Regulation of actin cytoskeleton - Homo sapiens (human) | 213 | 24 (11.3%) | 1.71e-06 | 0.000293 | KEGG |
| Signaling by VEGF | 100 | 15 (15.0%) | 4.86e-06 | 0.000552 | Reactome |
| Axon guidance | 358 | 32 (9.0%) | 5.36e-06 | 0.000552 | Reactome |
| Wnt | 69 | 12 (17.6%) | 7.85e-06 | 0.000674 | NetPath |
| MAPK Signaling Pathway | 246 | 24 (9.8%) | 2.05e-05 | 0.00151 | Wikipathways |
| RAC1 signaling pathway | 54 | 10 (18.5%) | 2.9e-05 | 0.00187 | PID |
| Regulation of Microtubule Cytoskeleton | 46 | 9 (19.6%) | 4.62e-05 | 0.00234 | Wikipathways |
| VEGFR2 mediated vascular permeability | 27 | 7 (25.9%) | 4.85e-05 | 0.00234 | Reactome |
| Ephrin signaling | 19 | 6 (31.6%) | 5.03e-05 | 0.00234 | Reactome |
| MAPK signaling pathway - Homo sapiens (human) | 295 | 26 (8.8%) | 5.44e-05 | 0.00234 | KEGG |
| Ras Signaling | 184 | 19 (10.3%) | 6.89e-05 | 0.00273 | Wikipathways |
| Developmental Biology | 620 | 43 (6.9%) | 8.31e-05 | 0.00295 | Reactome |
| EPHB forward signaling | 39 | 8 (20.5%) | 8.59e-05 | 0.00295 | PID |
| EPH-Ephrin signaling | 74 | 11 (14.9%) | 9.72e-05 | 0.00302 | Reactome |
| CXCR4-mediated signaling events | 87 | 12 (13.8%) | 9.98e-05 | 0.00302 | PID |
| Cell death signalling via NRAGE, NRIF and NADE | 78 | 11 (14.5%) | 0.000124 | 0.00337 | Reactome |
| Nectin adhesion pathway | 31 | 7 (22.6%) | 0.000126 | 0.00337 | PID |
| Endothelins | 64 | 10 (15.6%) | 0.000131 | 0.00337 | PID |
| Noncanonical Wnt signaling pathway | 32 | 7 (21.9%) | 0.000156 | 0.00382 | PID |
| Senescence and Autophagy in Cancer | 106 | 13 (12.3%) | 0.000176 | 0.00384 | Wikipathways |
| ErbB1 downstream signaling | 107 | 13 (12.3%) | 0.000176 | 0.00384 | PID |
| GnRH signaling pathway - Homo sapiens (human) | 93 | 12 (12.9%) | 0.000191 | 0.00384 | KEGG |
| CD28 co-stimulation | 33 | 7 (21.2%) | 0.000192 | 0.00384 | Reactome |
| Ras signaling pathway - Homo sapiens (human) | 232 | 21 (9.1%) | 0.000194 | 0.00384 | KEGG |
| Phase 0 - rapid depolarisation | 44 | 8 (18.2%) | 0.00021 | 0.004 | Reactome |
| Mesodermal Commitment Pathway | 153 | 16 (10.5%) | 0.00022 | 0.00404 | Wikipathways |
| Integrin | 124 | 14 (11.3%) | 0.000242 | 0.00431 | INOH |
| p75 NTR receptor-mediated signalling | 99 | 12 (12.5%) | 0.000258 | 0.00444 | Reactome |
| EPHB-mediated forward signaling | 35 | 7 (20.0%) | 0.000282 | 0.00469 | Reactome |
| Rap1 signaling pathway - Homo sapiens (human) | 206 | 19 (9.2%) | 0.000304 | 0.0049 | KEGG |
| PCP/CE pathway | 47 | 8 (17.0%) | 0.000337 | 0.00514 | Reactome |
| E-cadherin signaling in the nascent adherens junction | 36 | 7 (19.4%) | 0.000339 | 0.00514 | PID |
| Adrenergic signaling in cardiomyocytes - Homo sapiens (human) | 144 | 15 (10.4%) | 0.000357 | 0.00526 | KEGG |
| Rett syndrome causing genes | 48 | 8 (16.7%) | 0.000391 | 0.00558 | Wikipathways |
| RHO GTPases Activate WASPs and WAVEs | 37 | 7 (18.9%) | 0.000405 | 0.00558 | Reactome |
| Cardiac conduction | 131 | 14 (10.7%) | 0.000429 | 0.00558 | Reactome |
| Signaling by Rho GTPases | 435 | 31 (7.2%) | 0.000439 | 0.00558 | Reactome |
| Bacterial invasion of epithelial cells - Homo sapiens (human) | 74 | 10 (13.5%) | 0.000444 | 0.00558 | KEGG |
| Ion channel transport | 179 | 17 (9.5%) | 0.000445 | 0.00558 | Reactome |
| Focal Adhesion | 198 | 18 (9.1%) | 0.000518 | 0.00635 | Wikipathways |
| role of mal in rho-mediated activation of srf | 19 | 5 (26.3%) | 0.000565 | 0.00677 | BioCarta |
| Oxytocin signaling pathway - Homo sapiens (human) | 152 | 15 (9.9%) | 0.000636 | 0.00745 | KEGG |
| Renin secretion - Homo sapiens (human) | 65 | 9 (13.8%) | 0.00071 | 0.00795 | KEGG |
| Shigellosis - Homo sapiens (human) | 65 | 9 (13.8%) | 0.00071 | 0.00795 | KEGG |
| Beta-agonist/Beta-blocker Pathway, Pharmacodynamics | 66 | 9 (13.6%) | 0.000795 | 0.00871 | PharmGKB |
| Activated NOTCH1 Transmits Signal to the Nucleus | 32 | 6 (19.4%) | 0.000929 | 0.00997 | Reactome |
