# Supplementary material for: Differential methylation of microRNA encoding genes may contribute to high myopia
Source: Front Genet. 2023 Jan 4;13:1089784. doi: 10.3389/fgene.2022.1089784 (PMC9847511; doi:10.3389/fgene.2022.1089784)
Supplement: Supplementary file 4 [file Table9.DOCX]

**Supplementary Table 9. Identified signaling pathways and biological processes in overrepresentation analyses of target genes of the highest-ranked miRNAs - decreased methylation level**

| **Pathway name** | **Set size** | **Candidates contained** | **p-value** | **q-value** | **Pathway source** |
| --- | --- | --- | --- | --- | --- |
| ***MIR1178*** | | | | | |
| **miR-1178-3p** | | | | | |
| ErbB1 downstream signaling | 107 | 14 (13.2%) | 2.48e-07 | 0.000105 | PID |
| EGF-Core | 105 | 13 (12.4%) | 1.42e-06 | 0.000301 | Signalink |
| Wnt Signaling Pathway | 51 | 9 (17.6%) | 3.12e-06 | 0.000441 | Wikipathways |
| Insulin Signaling | 160 | 15 (9.4%) | 7.64e-06 | 0.000624 | Wikipathways |
| Transmission across Chemical Synapses | 224 | 18 (8.1%) | 7.93e-06 | 0.000624 | Reactome |
| EGF-EGFR Signaling Pathway | 162 | 15 (9.3%) | 8.89e-06 | 0.000624 | Wikipathways |
| EPO signaling | 186 | 16 (8.6%) | 1.07e-05 | 0.000624 | INOH |
| Prolactin Signaling Pathway | 76 | 10 (13.2%) | 1.37e-05 | 0.000624 | Wikipathways |
| Wnt signaling pathway - Homo sapiens (human) | 149 | 14 (9.5%) | 1.39e-05 | 0.000624 | KEGG |
| IL6-mediated signaling events | 47 | 8 (17.0%) | 1.47e-05 | 0.000624 | PID |
| Physiological and Pathological Hypertrophy of the Heart | 24 | 6 (25.0%) | 1.77e-05 | 0.000682 | Wikipathways |
| Trk receptor signaling mediated by PI3K and PLC-gamma | 36 | 7 (19.4%) | 2.06e-05 | 0.000727 | PID |
| B Cell Receptor Signaling Pathway | 98 | 11 (11.2%) | 2.38e-05 | 0.000775 | Wikipathways |
| Signaling events mediated by Stem cell factor receptor (c-Kit) | 52 | 8 (15.4%) | 3.17e-05 | 0.000878 | PID |
| L1CAM interactions | 103 | 11 (10.8%) | 3.47e-05 | 0.000878 | Reactome |
| IL-7 signaling | 185 | 15 (8.2%) | 4.04e-05 | 0.000878 | INOH |
| Colorectal cancer - Homo sapiens (human) | 86 | 10 (11.6%) | 4.1e-05 | 0.000878 | KEGG |
| IL1 | 54 | 8 (14.8%) | 4.21e-05 | 0.000878 | NetPath |
| ErbB2/ErbB3 signaling events | 40 | 7 (17.5%) | 4.23e-05 | 0.000878 | PID |
| Prolactin | 70 | 9 (12.9%) | 4.46e-05 | 0.000878 | NetPath |
| EGF | 28 | 6 (21.4%) | 4.57e-05 | 0.000878 | INOH |
| IL-1 signaling pathway | 55 | 8 (14.5%) | 4.82e-05 | 0.000878 | Wikipathways |
| VEGF | 188 | 15 (8.0%) | 4.88e-05 | 0.000878 | INOH |
| JAK-STAT | 106 | 11 (10.4%) | 4.97e-05 | 0.000878 | Wikipathways |
| VEGFA-VEGFR2 Signaling Pathway | 236 | 17 (7.2%) | 6.08e-05 | 0.00103 | Wikipathways |
| IL-6 signaling pathway | 43 | 7 (16.3%) | 6.87e-05 | 0.0011 | Wikipathways |
| IL6 | 74 | 9 (12.2%) | 6.97e-05 | 0.0011 | NetPath |
| T-Cell antigen Receptor (TCR) Signaling Pathway | 92 | 10 (10.9%) | 7.36e-05 | 0.00111 | Wikipathways |
| Neurotransmitter receptors and postsynaptic signal transmission | 153 | 13 (8.6%) | 8.07e-05 | 0.00118 | Reactome |
| Leptin signaling pathway | 76 | 9 (11.8%) | 8.62e-05 | 0.00122 | Wikipathways |
| Wnt Signaling | 114 | 11 (9.6%) | 9.69e-05 | 0.00133 | Wikipathways |
| MAPK Signaling Pathway | 246 | 17 (6.9%) | 0.000101 | 0.00134 | Wikipathways |
| EGFR1 | 457 | 25 (5.5%) | 0.000115 | 0.00147 | NetPath |
| Neurotrophin signaling pathway - Homo sapiens (human) | 119 | 11 (9.2%) | 0.000143 | 0.00178 | KEGG |
| Axon guidance | 358 | 21 (5.9%) | 0.000161 | 0.00191 | Reactome |
| IL-3 Signaling Pathway | 49 | 7 (14.3%) | 0.000162 | 0.00191 | Wikipathways |
| angiotensin ii mediated activation of jnk pathway via pyk2 dependent signaling | 35 | 6 (17.1%) | 0.000171 | 0.00196 | BioCarta |
| JAK STAT pathway and regulation | 310 | 19 (6.1%) | 0.000187 | 0.00209 | INOH |
| Developmental Biology | 620 | 30 (4.8%) | 0.000226 | 0.00246 | Reactome |
| PDGFR-beta signaling pathway | 127 | 11 (8.7%) | 0.000254 | 0.00267 | PID |
| Intracellular Signalling Through Adenosine Receptor A2a and Adenosine | 38 | 6 (15.8%) | 0.000273 | 0.00267 | SMPDB |
| Intracellular Signalling Through Adenosine Receptor A2b and Adenosine | 38 | 6 (15.8%) | 0.000273 | 0.00267 | SMPDB |
| Prolactin signaling pathway - Homo sapiens (human) | 70 | 8 (11.4%) | 0.000273 | 0.00267 | KEGG |
| Androgen receptor signaling pathway | 89 | 9 (10.1%) | 0.000292 | 0.00267 | Wikipathways |
| IL2-mediated signaling events | 54 | 7 (13.0%) | 0.000301 | 0.00267 | PID |
| Cardiac Hypertrophic Response | 54 | 7 (13.0%) | 0.000301 | 0.00267 | Wikipathways |
| CDC42 signaling events | 71 | 8 (11.3%) | 0.000302 | 0.00267 | PID |
| MAPK signaling pathway - Homo sapiens (human) | 295 | 18 (6.1%) | 0.000303 | 0.00267 | KEGG |
| Angiopoietin Like Protein 8 Regulatory Pathway | 131 | 11 (8.4%) | 0.000332 | 0.00286 | Wikipathways |
| RANKL-RANK (Receptor activator of NFKB (ligand)) Signaling Pathway | 55 | 7 (12.7%) | 0.000338 | 0.00286 | Wikipathways |
| Chromosomal and microsatellite instability in colorectal cancer | 73 | 8 (11.0%) | 0.000365 | 0.00304 | Wikipathways |
| GnRH signaling pathway - Homo sapiens (human) | 93 | 9 (9.7%) | 0.000407 | 0.0032 | KEGG |
| transcription factor creb and its extracellular signals | 27 | 5 (18.5%) | 0.000416 | 0.0032 | BioCarta |
| TNF related weak inducer of apoptosis (TWEAK) Signaling Pathway | 41 | 6 (14.6%) | 0.000419 | 0.0032 | Wikipathways |
| Gastrin | 41 | 6 (14.6%) | 0.000419 | 0.0032 | NetPath |
| mapkinase signaling pathway | 57 | 7 (12.3%) | 0.000422 | 0.0032 | BioCarta |
| role of erbb2 in signal transduction and oncology | 28 | 5 (17.9%) | 0.000497 | 0.00363 | BioCarta |
| TWEAK | 28 | 5 (17.9%) | 0.000497 | 0.00363 | NetPath |
| Kit receptor signaling pathway | 59 | 7 (11.9%) | 0.000523 | 0.00376 | Wikipathways |
| Signaling pathways regulating pluripotency of stem cells - Homo sapiens (human) | 139 | 11 (7.9%) | 0.000552 | 0.0039 | KEGG |
| TNFalpha | 234 | 15 (6.4%) | 0.000573 | 0.00397 | NetPath |
| ATF-2 transcription factor network | 60 | 7 (11.7%) | 0.00058 | 0.00397 | PID |
| Neuronal System | 368 | 20 (5.4%) | 0.000614 | 0.00413 | Reactome |
| Angiogenesis overview | 61 | 7 (11.5%) | 0.000642 | 0.00425 | Wikipathways |
| Activation of BH3-only proteins | 30 | 5 (16.7%) | 0.000692 | 0.00452 | Reactome |
| Leptin | 62 | 7 (11.3%) | 0.000709 | 0.00455 | NetPath |
| Synthesis of substrates in N-glycan biosythesis | 63 | 7 (11.1%) | 0.000781 | 0.00494 | Reactome |
| Trafficking of AMPA receptors | 31 | 5 (16.1%) | 0.000809 | 0.00497 | Reactome |
| Glutamate binding, activation of AMPA receptors and synaptic plasticity | 31 | 5 (16.1%) | 0.000809 | 0.00497 | Reactome |
| Oocyte meiosis - Homo sapiens (human) | 125 | 10 (8.0%) | 0.000899 | 0.00544 | KEGG |
| Intracellular signaling by second messengers | 245 | 15 (6.1%) | 0.000919 | 0.00549 | Reactome |
| ErbB signaling pathway - Homo sapiens (human) | 85 | 8 (9.4%) | 0.00102 | 0.006 | KEGG |
| AGE-RAGE pathway | 66 | 7 (10.6%) | 0.00103 | 0.006 | Wikipathways |
| bioactive peptide induced signaling pathway | 33 | 5 (15.2%) | 0.00109 | 0.00623 | BioCarta |
| cAMP signaling pathway - Homo sapiens (human) | 199 | 13 (6.5%) | 0.00111 | 0.00623 | KEGG |
| EGFR Inhibitor Pathway, Pharmacodynamics | 67 | 7 (10.4%) | 0.00113 | 0.00623 | PharmGKB |
| Fc epsilon RI signaling pathway - Homo sapiens (human) | 68 | 7 (10.4%) | 0.00113 | 0.00623 | KEGG |
| FoxO family signaling | 50 | 6 (12.0%) | 0.00123 | 0.00654 | PID |
| BCR signaling pathway | 68 | 7 (10.3%) | 0.00124 | 0.00654 | PID |
| RAC1-PAK1-p38-MMP2 Pathway | 68 | 7 (10.3%) | 0.00124 | 0.00654 | Wikipathways |
| Signaling of Hepatocyte Growth Factor Receptor | 34 | 5 (14.7%) | 0.00125 | 0.00654 | Wikipathways |
| Hepatitis C - Homo sapiens (human) | 155 | 11 (7.1%) | 0.00136 | 0.00691 | KEGG |
| IL3 | 51 | 6 (11.8%) | 0.00137 | 0.00691 | NetPath |
| Insulin receptor signalling cascade | 51 | 6 (11.8%) | 0.00137 | 0.00691 | Reactome |
| KitReceptor | 70 | 7 (10.0%) | 0.00147 | 0.00731 | NetPath |
| ErbB Signaling Pathway | 92 | 8 (8.7%) | 0.0017 | 0.00831 | Wikipathways |
| Corticotropin-releasing hormone signaling pathway | 92 | 8 (8.7%) | 0.0017 | 0.00831 | Wikipathways |
| TNF alpha Signaling Pathway | 93 | 8 (8.6%) | 0.00183 | 0.00861 | Wikipathways |
| IL2 signaling events mediated by PI3K | 37 | 5 (13.5%) | 0.00184 | 0.00861 | PID |
| IL-4 Signaling Pathway | 54 | 6 (11.1%) | 0.00185 | 0.00861 | Wikipathways |
| RAC1 signaling pathway | 54 | 6 (11.1%) | 0.00185 | 0.00861 | PID |
| Fibroblast growth factor-1 | 74 | 7 (9.5%) | 0.00203 | 0.00934 | NetPath |
| Signaling events mediated by TCPTP | 38 | 5 (13.2%) | 0.00208 | 0.0095 | PID |
| PIP3 activates AKT signaling | 214 | 13 (6.1%) | 0.00213 | 0.00959 | Reactome |
| Pancreatic cancer - Homo sapiens (human) | 75 | 7 (9.3%) | 0.00219 | 0.00973 | KEGG |
| Role of Calcineurin-dependent NFAT signaling in lymphocytes | 56 | 6 (10.7%) | 0.00223 | 0.00973 | PID |
| Sphingolipid signaling pathway - Homo sapiens (human) | 118 | 9 (7.6%) | 0.00224 | 0.00973 | KEGG |
| Signaling events regulated by Ret tyrosine kinase | 39 | 5 (12.8%) | 0.00234 | 0.00973 | PID |
| Viral Acute Myocarditis | 76 | 7 (9.2%) | 0.00236 | 0.00973 | Wikipathways |
| IL2 | 76 | 7 (9.2%) | 0.00236 | 0.00973 | NetPath |
| MyD88:Mal cascade initiated on plasma membrane | 97 | 8 (8.2%) | 0.00239 | 0.00973 | Reactome |
| Toll Like Receptor TLR1:TLR2 Cascade | 97 | 8 (8.2%) | 0.00239 | 0.00973 | Reactome |
| Toll Like Receptor TLR6:TLR2 Cascade | 97 | 8 (8.2%) | 0.00239 | 0.00973 | Reactome |
| Toll Like Receptor 2 (TLR2) Cascade | 97 | 8 (8.2%) | 0.00239 | 0.00973 | Reactome |
| **miR-1178-5p and miR-1178-3p** | | | | | |
| ErbB1 downstream signaling | 107 | 17 (16.0%) | 2.87e-07 | 0.000168 | PID |
| EGF-Core | 105 | 16 (15.2%) | 1.3e-06 | 0.000381 | Signalink |
| B Cell Receptor Signaling Pathway | 98 | 15 (15.3%) | 2.64e-06 | 0.000514 | Wikipathways |
| IL-1 signaling pathway | 55 | 11 (20.0%) | 4.03e-06 | 0.000589 | Wikipathways |
| IL6-mediated signaling events | 47 | 10 (21.3%) | 6.17e-06 | 0.000721 | PID |
| EPO signaling | 186 | 20 (10.8%) | 1.52e-05 | 0.00147 | INOH |
| VEGFA-VEGFR2 Signaling Pathway | 236 | 23 (9.7%) | 1.96e-05 | 0.00147 | Wikipathways |
| IL1 | 54 | 10 (18.5%) | 2.27e-05 | 0.00147 | NetPath |
| Insulin Signaling | 160 | 18 (11.2%) | 2.35e-05 | 0.00147 | Wikipathways |
| HDAC6 interactions | 11 | 5 (45.5%) | 2.51e-05 | 0.00147 | Wikipathways |
| EGF-EGFR Signaling Pathway | 162 | 18 (11.1%) | 2.78e-05 | 0.00148 | Wikipathways |
| MAPK Signaling Pathway | 246 | 23 (9.3%) | 3.8e-05 | 0.0017 | Wikipathways |
| IL-7 signaling | 185 | 19 (10.3%) | 4.68e-05 | 0.0017 | INOH |
| MyD88:Mal cascade initiated on plasma membrane | 97 | 13 (13.4%) | 5.21e-05 | 0.0017 | Reactome |
| Toll Like Receptor TLR1:TLR2 Cascade | 97 | 13 (13.4%) | 5.21e-05 | 0.0017 | Reactome |
| Toll Like Receptor TLR6:TLR2 Cascade | 97 | 13 (13.4%) | 5.21e-05 | 0.0017 | Reactome |
| Toll Like Receptor 2 (TLR2) Cascade | 97 | 13 (13.4%) | 5.21e-05 | 0.0017 | Reactome |
| Intracellular Signalling Through Adenosine Receptor A2b and Adenosine | 38 | 8 (21.1%) | 5.76e-05 | 0.0017 | SMPDB |
| Intracellular Signalling Through Adenosine Receptor A2a and Adenosine | 38 | 8 (21.1%) | 5.76e-05 | 0.0017 | SMPDB |
| VEGF | 188 | 19 (10.2%) | 5.84e-05 | 0.0017 | INOH |
| EGFR1 | 457 | 34 (7.5%) | 6.76e-05 | 0.0017 | NetPath |
| Transmission across Chemical Synapses | 224 | 21 (9.4%) | 7.39e-05 | 0.0017 | Reactome |
| IL6 | 74 | 11 (14.9%) | 7.51e-05 | 0.0017 | NetPath |
| MyD88 cascade initiated on plasma membrane | 87 | 12 (13.8%) | 7.58e-05 | 0.0017 | Reactome |
| Toll Like Receptor 10 (TLR10) Cascade | 87 | 12 (13.8%) | 7.58e-05 | 0.0017 | Reactome |
| Toll Like Receptor 5 (TLR5) Cascade | 87 | 12 (13.8%) | 7.58e-05 | 0.0017 | Reactome |
| TRAF6 mediated induction of NFkB and MAP kinases upon TLR7/8 or 9 activation | 88 | 12 (13.6%) | 8.49e-05 | 0.00172 | Reactome |
| ErbB2/ErbB3 signaling events | 40 | 8 (20.0%) | 8.49e-05 | 0.00172 | PID |
| Wnt Signaling Pathway | 51 | 9 (17.6%) | 8.65e-05 | 0.00172 | Wikipathways |
| MAPK signaling pathway - Homo sapiens (human) | 295 | 25 (8.5%) | 8.95e-05 | 0.00172 | KEGG |
| Prolactin Signaling Pathway | 76 | 11 (14.5%) | 9.63e-05 | 0.00172 | Wikipathways |
| Leptin signaling pathway | 76 | 11 (14.5%) | 9.63e-05 | 0.00172 | Wikipathways |
| Signaling events mediated by Stem cell factor receptor (c-Kit) | 52 | 9 (17.3%) | 0.000101 | 0.00172 | PID |
| Gastrin | 41 | 8 (19.5%) | 0.000102 | 0.00172 | NetPath |
| MyD88 dependent cascade initiated on endosome | 90 | 12 (13.3%) | 0.000106 | 0.00172 | Reactome |
| Toll Like Receptor 7/8 (TLR7/8) Cascade | 90 | 12 (13.3%) | 0.000106 | 0.00172 | Reactome |
| MAP kinase activation | 65 | 10 (15.4%) | 0.000118 | 0.00182 | Reactome |
| Interleukin-17 signaling | 65 | 10 (15.4%) | 0.000118 | 0.00182 | Reactome |
| JAK-STAT | 106 | 13 (12.3%) | 0.000132 | 0.00195 | Wikipathways |
| AGE-RAGE pathway | 66 | 10 (15.2%) | 0.000135 | 0.00195 | Wikipathways |
| RAC1 signaling pathway | 54 | 9 (16.7%) | 0.000137 | 0.00195 | PID |
| IL-6 signaling pathway | 43 | 8 (18.6%) | 0.000145 | 0.00202 | Wikipathways |
| Toll Like Receptor 9 (TLR9) Cascade | 94 | 12 (12.8%) | 0.000162 | 0.0022 | Reactome |
| Physiological and Pathological Hypertrophy of the Heart | 24 | 6 (25.0%) | 0.000182 | 0.00241 | Wikipathways |
| JAK STAT pathway and regulation | 310 | 25 (8.1%) | 0.000186 | 0.00241 | INOH |
| mapkinase signaling pathway | 57 | 9 (15.8%) | 0.00021 | 0.00267 | BioCarta |
| Toll Like Receptor 3 (TLR3) Cascade | 97 | 12 (12.4%) | 0.000219 | 0.0027 | Reactome |
| Prolactin | 70 | 10 (14.3%) | 0.000222 | 0.0027 | NetPath |
| Toll Like Receptor 4 (TLR4) Cascade | 127 | 14 (11.0%) | 0.000232 | 0.00276 | Reactome |
| angiotensin ii mediated activation of jnk pathway via pyk2 dependent signaling | 35 | 7 (20.0%) | 0.000237 | 0.00276 | BioCarta |
| CDC42 signaling events | 71 | 10 (14.1%) | 0.00025 | 0.00287 | PID |
| Brain-Derived Neurotrophic Factor (BDNF) signaling pathway | 144 | 15 (10.4%) | 0.000263 | 0.00292 | Wikipathways |
| AGE-RAGE signaling pathway in diabetic complications - Homo sapiens (human) | 99 | 12 (12.1%) | 0.000265 | 0.00292 | KEGG |
| Trk receptor signaling mediated by PI3K and PLC-gamma | 36 | 7 (19.4%) | 0.000285 | 0.00302 | PID |
| IL1-mediated signaling events | 36 | 7 (19.4%) | 0.000285 | 0.00302 | PID |
| Colorectal cancer - Homo sapiens (human) | 86 | 11 (12.8%) | 0.000296 | 0.00309 | KEGG |
| TRIF(TICAM1)-mediated TLR4 signaling | 101 | 12 (11.9%) | 0.00032 | 0.00317 | Reactome |
| MyD88-independent TLR4 cascade | 101 | 12 (11.9%) | 0.00032 | 0.00317 | Reactome |
| Angiopoietin Like Protein 8 Regulatory Pathway | 131 | 14 (10.7%) | 0.000321 | 0.00317 | Wikipathways |
| TGF-beta Signaling Pathway | 132 | 14 (10.6%) | 0.000347 | 0.00335 | Wikipathways |
| L1CAM interactions | 103 | 12 (11.8%) | 0.000351 | 0.00335 | Reactome |
| Angiogenesis overview | 61 | 9 (14.8%) | 0.000356 | 0.00335 | Wikipathways |
| IL-3 Signaling Pathway | 49 | 8 (16.3%) | 0.000373 | 0.00345 | Wikipathways |
| Sphingolipid signaling pathway - Homo sapiens (human) | 118 | 13 (11.0%) | 0.000387 | 0.00353 | KEGG |
| TNFalpha | 234 | 20 (8.5%) | 0.000399 | 0.00358 | NetPath |
| Neurotrophin signaling pathway - Homo sapiens (human) | 119 | 13 (10.9%) | 0.00042 | 0.00372 | KEGG |
| role of erbb2 in signal transduction and oncology | 28 | 6 (21.4%) | 0.000449 | 0.00385 | BioCarta |
| EGF | 28 | 6 (21.4%) | 0.000449 | 0.00385 | INOH |
| T-Cell antigen Receptor (TCR) Signaling Pathway | 92 | 11 (12.0%) | 0.000534 | 0.00452 | Wikipathways |
| Toll-Like Receptors Cascades | 154 | 15 (9.7%) | 0.000542 | 0.00452 | Reactome |
| Shigellosis - Homo sapiens (human) | 65 | 9 (13.8%) | 0.000577 | 0.00469 | KEGG |
| Hepatitis C - Homo sapiens (human) | 155 | 15 (9.7%) | 0.00058 | 0.00469 | KEGG |
| TNF alpha Signaling Pathway | 93 | 11 (11.8%) | 0.000586 | 0.00469 | Wikipathways |
| TGF-beta signaling TAK1 | 20 | 5 (25.0%) | 0.000641 | 0.00499 | INOH |
| SUMOylation of transcription factors | 20 | 5 (25.0%) | 0.000641 | 0.00499 | Reactome |
| Cardiac Hypertrophic Response | 54 | 8 (14.8%) | 0.000732 | 0.00563 | Wikipathways |
| Splicing factor NOVA regulated synaptic proteins | 42 | 7 (16.7%) | 0.000761 | 0.00577 | Wikipathways |
| MAPK targets/ Nuclear events mediated by MAP kinases | 31 | 6 (19.4%) | 0.000799 | 0.00595 | Reactome |
| RAC1-PAK1-p38-MMP2 Pathway | 68 | 9 (13.2%) | 0.000807 | 0.00595 | Wikipathways |
| TSLP | 21 | 5 (23.8%) | 0.000816 | 0.00595 | NetPath |
| RANKL-RANK (Receptor activator of NFKB (ligand)) Signaling Pathway | 55 | 8 (14.5%) | 0.00083 | 0.00599 | Wikipathways |
| Role of Calcineurin-dependent NFAT signaling in lymphocytes | 56 | 8 (14.3%) | 0.000938 | 0.00668 | PID |
| Prolactin signaling pathway - Homo sapiens (human) | 70 | 9 (12.9%) | 0.000998 | 0.00702 | KEGG |
| Neuronal System | 368 | 26 (7.1%) | 0.00106 | 0.00737 | Reactome |
| Wnt signaling pathway - Homo sapiens (human) | 149 | 14 (9.5%) | 0.00109 | 0.00752 | KEGG |
| bioactive peptide induced signaling pathway | 33 | 6 (18.2%) | 0.00113 | 0.00767 | BioCarta |
| Developmental Biology | 620 | 38 (6.1%) | 0.00131 | 0.0088 | Reactome |
| Kit receptor signaling pathway | 59 | 8 (13.6%) | 0.00133 | 0.00883 | Wikipathways |
| Chromosomal and microsatellite instability in colorectal cancer | 73 | 9 (12.3%) | 0.00135 | 0.00887 | Wikipathways |
| Energy Metabolism | 47 | 7 (14.9%) | 0.00151 | 0.00977 | Wikipathways |
| Androgen receptor signaling pathway | 89 | 10 (11.2%) | 0.00153 | 0.00977 | Wikipathways |
| Axon guidance | 358 | 25 (7.0%) | 0.00154 | 0.00977 | Reactome |
| Fas | 24 | 5 (20.8%) | 0.00156 | 0.00977 | INOH |
| ***MIRLET7A2*** | | | | | |
| **let-7a-5p (previously let-7a)** | | | | | |
| Angiopoietin Like Protein 8 Regulatory Pathway | 131 | 22 (16.8%) | 1.3e-06 | 0.000881 | Wikipathways |
| AGE-RAGE signaling pathway in diabetic complications - Homo sapiens (human) | 99 | 18 (18.2%) | 3.73e-06 | 0.00123 | KEGG |
| miR-targeted genes in lymphocytes - TarBase | 489 | 50 (10.2%) | 5.43e-06 | 0.00123 | Wikipathways |
| Protein alkylation leading to liver fibrosis | 50 | 12 (24.0%) | 8.23e-06 | 0.00139 | Wikipathways |
| FoxO signaling pathway - Homo sapiens (human) | 132 | 20 (15.3%) | 1.72e-05 | 0.00232 | KEGG |
| Hepatitis C and Hepatocellular Carcinoma | 56 | 12 (21.4%) | 2.83e-05 | 0.00307 | Wikipathways |
| Scavenging by Class A Receptors | 19 | 7 (36.8%) | 3.23e-05 | 0.00307 | Reactome |
| miR-targeted genes in muscle cell - TarBase | 400 | 41 (10.2%) | 3.62e-05 | 0.00307 | Wikipathways |
| Collagen chain trimerization | 44 | 10 (22.7%) | 7.72e-05 | 0.00581 | Reactome |
| Collagen formation | 92 | 15 (16.3%) | 8.87e-05 | 0.00601 | Reactome |
| Insulin Signaling | 160 | 21 (13.1%) | 0.000105 | 0.00624 | Wikipathways |
| Type II diabetes mellitus - Homo sapiens (human) | 46 | 10 (21.7%) | 0.000115 | 0.00624 | KEGG |
| EGF-Core | 105 | 16 (15.2%) | 0.00012 | 0.00624 | Signalink |
| Collagen biosynthesis and modifying enzymes | 68 | 12 (17.6%) | 0.000206 | 0.00968 | Reactome |
| SHP2 signaling | 59 | 11 (18.6%) | 0.000228 | 0.00968 | PID |
| Bladder cancer - Homo sapiens (human) | 41 | 9 (22.0%) | 0.000233 | 0.00968 | KEGG |
| PI3K-Akt Signaling Pathway | 340 | 34 (10.0%) | 0.00025 | 0.00968 | Wikipathways |
| MAPK Signaling Pathway | 246 | 27 (11.0%) | 0.000257 | 0.00968 | Wikipathways |
| Prolactin signaling pathway - Homo sapiens (human) | 70 | 12 (17.1%) | 0.000273 | 0.00974 | KEGG |
| **let-7a-2-3p (previously let-7a-2)** | | | | | |
| Adipogenesis | 131 | 25 (19.1%) | 9.86e-09 | 6.9e-06 | Wikipathways |
| Prion disease pathway | 33 | 12 (36.4%) | 3.92e-08 | 1.37e-05 | Wikipathways |
| Ectoderm Differentiation | 142 | 23 (16.2%) | 8.45e-07 | 0.000197 | Wikipathways |
| Developmental Biology | 620 | 59 (9.5%) | 2.71e-06 | 0.000474 | Reactome |
| Signaling by Receptor Tyrosine Kinases | 423 | 43 (10.2%) | 1.35e-05 | 0.0018 | Reactome |
| Axon guidance | 358 | 38 (10.6%) | 1.54e-05 | 0.0018 | Reactome |
| JAK-STAT | 106 | 17 (16.0%) | 2.57e-05 | 0.00257 | Wikipathways |
| EGFR1 | 457 | 44 (9.7%) | 3.68e-05 | 0.00302 | NetPath |
| Receptor-type tyrosine-protein phosphatases | 20 | 7 (35.0%) | 3.88e-05 | 0.00302 | Reactome |
| MECP2 and Associated Rett Syndrome | 63 | 12 (19.0%) | 7.15e-05 | 0.00487 | Wikipathways |
| EGF-EGFR Signaling Pathway | 162 | 21 (13.0%) | 8.15e-05 | 0.00487 | Wikipathways |
| EGF-Core | 105 | 16 (15.2%) | 8.35e-05 | 0.00487 | Signalink |
| Prolactin Signaling Pathway | 76 | 13 (17.1%) | 0.000115 | 0.00621 | Wikipathways |
| White fat cell differentiation | 32 | 8 (25.0%) | 0.00016 | 0.00802 | Wikipathways |
| Integrin-mediated Cell Adhesion | 101 | 15 (14.9%) | 0.000185 | 0.00864 | Wikipathways |
| EPHA forward signaling | 33 | 8 (24.2%) | 0.000202 | 0.00886 | PID |
| **let-7a-5p (previously let-7a) and let-7a-2-3p (previously let-7a-2)** | | | | | |
| Adipogenesis | 131 | 33 (25.2%) | 2.61e-07 | 0.00023 | Wikipathways |
| miR-targeted genes in lymphocytes - TarBase | 489 | 83 (17.0%) | 3.64e-07 | 0.00023 | Wikipathways |
| EGF-Core | 105 | 28 (26.7%) | 5.99e-07 | 0.000242 | Signalink |
| Angiopoietin Like Protein 8 Regulatory Pathway | 131 | 32 (24.4%) | 8.3e-07 | 0.000242 | Wikipathways |
| Prion disease pathway | 33 | 14 (42.4%) | 9.56e-07 | 0.000242 | Wikipathways |
| Pathways Affected in Adenoid Cystic Carcinoma | 63 | 20 (31.7%) | 1.26e-06 | 0.000243 | Wikipathways |
| Interleukin-4 and Interleukin-13 signaling | 97 | 26 (26.8%) | 1.35e-06 | 0.000243 | Wikipathways |
| AGE-RAGE signaling pathway in diabetic complications - Homo sapiens (human) | 99 | 26 (26.3%) | 2.05e-06 | 0.000322 | KEGG |
| JAK-STAT | 106 | 27 (25.5%) | 2.5e-06 | 0.000322 | Wikipathways |
| Axon guidance | 358 | 63 (17.6%) | 2.55e-06 | 0.000322 | Reactome |
| Developmental Biology | 620 | 96 (15.5%) | 2.99e-06 | 0.000343 | Reactome |
| Ectoderm Differentiation | 142 | 32 (22.5%) | 5.35e-06 | 0.000563 | Wikipathways |
| miR-targeted genes in muscle cell - TarBase | 400 | 67 (16.8%) | 7.79e-06 | 0.000756 | Wikipathways |
| TGF-beta Signaling Pathway | 132 | 30 (22.7%) | 8.72e-06 | 0.000786 | Wikipathways |
| Insulin Signaling | 160 | 34 (21.2%) | 1.07e-05 | 0.000902 | Wikipathways |
| EGF-EGFR Signaling Pathway | 162 | 34 (21.0%) | 1.42e-05 | 0.00108 | Wikipathways |
| EGFR1 | 457 | 73 (16.0%) | 1.45e-05 | 0.00108 | NetPath |
| Gene expression (Transcription) | 1373 | 179 (13.1%) | 1.57e-05 | 0.0011 | Reactome |
| Focal Adhesion | 198 | 39 (19.7%) | 1.65e-05 | 0.0011 | Wikipathways |
| Focal adhesion - Homo sapiens (human) | 199 | 39 (19.6%) | 1.86e-05 | 0.00117 | KEGG |
| Integrin | 124 | 28 (22.6%) | 1.95e-05 | 0.00118 | INOH |
| FoxO signaling pathway - Homo sapiens (human) | 132 | 29 (22.1%) | 2.08e-05 | 0.0012 | KEGG |
| Signaling by Receptor Tyrosine Kinases | 423 | 68 (16.1%) | 2.67e-05 | 0.00146 | Reactome |
| Prolactin Signaling Pathway | 76 | 20 (26.3%) | 2.94e-05 | 0.00154 | Wikipathways |
| p53 signaling pathway - Homo sapiens (human) | 72 | 19 (26.4%) | 4.43e-05 | 0.00218 | KEGG |
| Transcriptional misregulation in cancer - Homo sapiens (human) | 186 | 36 (19.5%) | 4.5e-05 | 0.00218 | KEGG |
| Prostate cancer - Homo sapiens (human) | 97 | 23 (23.7%) | 4.69e-05 | 0.00219 | KEGG |
| Photodynamic therapy-induced AP-1 survival signaling. | 50 | 15 (30.0%) | 5.6e-05 | 0.0025 | Wikipathways |
| Generic Transcription Pathway | 1107 | 146 (13.3%) | 5.73e-05 | 0.0025 | Reactome |
| Leptin signaling pathway | 76 | 19 (25.0%) | 9.84e-05 | 0.00407 | Wikipathways |
| RNA Polymerase II Transcription | 1236 | 159 (12.9%) | 9.99e-05 | 0.00407 | Reactome |
| Energy Metabolism | 47 | 14 (29.8%) | 0.000107 | 0.00424 | Wikipathways |
| Brain-Derived Neurotrophic Factor (BDNF) signaling pathway | 144 | 29 (20.1%) | 0.000128 | 0.00491 | Wikipathways |
| DNA Damage Response (only ATM dependent) | 110 | 24 (21.8%) | 0.000133 | 0.00494 | Wikipathways |
| EPHA forward signaling | 33 | 11 (33.3%) | 0.000194 | 0.00699 | PID |
| Protein alkylation leading to liver fibrosis | 50 | 14 (28.0%) | 0.000223 | 0.0072 | Wikipathways |
| PI3K-Akt Signaling Pathway | 340 | 54 (15.9%) | 0.000223 | 0.0072 | Wikipathways |
| Sterol Regulatory Element-Binding Proteins (SREBP) signalling | 68 | 17 (25.0%) | 0.000225 | 0.0072 | Wikipathways |
| Hepatitis C and Hepatocellular Carcinoma | 56 | 15 (26.8%) | 0.00023 | 0.0072 | Wikipathways |
| Scavenging by Class A Receptors | 19 | 8 (42.1%) | 0.000234 | 0.0072 | Reactome |
| Mitochondrial Gene Expression | 19 | 8 (42.1%) | 0.000234 | 0.0072 | Wikipathways |
| mTOR signaling pathway - Homo sapiens (human) | 151 | 29 (19.3%) | 0.000268 | 0.00793 | KEGG |
| Glycosaminoglycan biosynthesis - heparan sulfate / heparin - Homo sapiens (human) | 24 | 9 (37.5%) | 0.000271 | 0.00793 | KEGG |
| Focal Adhesion-PI3K-Akt-mTOR-signaling pathway | 302 | 49 (16.2%) | 0.000276 | 0.00793 | Wikipathways |
| Caspase Cascade in Apoptosis | 57 | 15 (26.3%) | 0.000285 | 0.00799 | PID |
| MAPK signaling pathway - Homo sapiens (human) | 295 | 48 (16.3%) | 0.000297 | 0.00815 | KEGG |
| Hepatitis B - Homo sapiens (human) | 144 | 28 (19.4%) | 0.000308 | 0.00827 | KEGG |
| Prolactin signaling pathway - Homo sapiens (human) | 70 | 17 (24.3%) | 0.000327 | 0.00861 | KEGG |
| BDNF | 46 | 13 (28.3%) | 0.000336 | 0.00865 | NetPath |
| PI3K-Akt signaling pathway - Homo sapiens (human) | 354 | 55 (15.6%) | 0.000346 | 0.00875 | KEGG |
| Receptor-type tyrosine-protein phosphatases | 20 | 8 (40.0%) | 0.000356 | 0.00882 | Reactome |
| Factors and pathways affecting insulin-like growth factor (IGF1)-Akt signaling | 30 | 10 (33.3%) | 0.000378 | 0.00904 | Wikipathways |
| Mesodermal Commitment Pathway | 153 | 29 (19.0%) | 0.00038 | 0.00904 | Wikipathways |
| IL4-mediated signaling events | 65 | 16 (24.6%) | 0.000413 | 0.00965 | PID |
| ***MIR885*** | | | | | |
| **hsa-miR-885-3p** | | | | | |
| Choline metabolism in cancer - Homo sapiens (human) | 98 | 8 (8.2%) | 3.78e-05 | 0.00378 | KEGG |
| EGFR1 | 455 | 17 (3.7%) | 7.89e-05 | 0.00395 | NetPath |
| BCR | 129 | 8 (6.2%) | 0.000261 | 0.00871 | NetPath |
| ***MIR548I3*** | | | | | |
| **miR-548i** | | | | | |
| FoxO signaling pathway - Homo sapiens (human) | 132 | 27 (20.6%) | 3.17e-06 | 0.0032 | KEGG |
| ***MIR6854*** | | | | | |
| **miR-6854-3p** | | | | | |
| Axon guidance - Homo sapiens (human) | 175 | 6 (3.4%) | 5.33e-05 | 0.000479 | KEGG |
| Ras signaling pathway - Homo sapiens (human) | 232 | 5 (2.2%) | 0.00191 | 0.00709 | KEGG |
| Axon guidance | 358 | 6 (1.7%) | 0.00236 | 0.00709 | Reactome |
| ***MIRLET7C*** | | | | | |
| **let-7c-5p** | | | | | |
| Angiopoietin Like Protein 8 Regulatory Pathway | 131 | 22 (16.8%) | 1.3e-06 | 0.000881 | Wikipathways |
| AGE-RAGE signaling pathway in diabetic complications - Homo sapiens (human) | 99 | 18 (18.2%) | 3.73e-06 | 0.00123 | KEGG |
| miR-targeted genes in lymphocytes - TarBase | 489 | 50 (10.2%) | 5.43e-06 | 0.00123 | Wikipathways |
| Protein alkylation leading to liver fibrosis | 50 | 12 (24.0%) | 8.23e-06 | 0.00139 | Wikipathways |
| FoxO signaling pathway - Homo sapiens (human) | 132 | 20 (15.3%) | 1.72e-05 | 0.00232 | KEGG |
| Hepatitis C and Hepatocellular Carcinoma | 56 | 12 (21.4%) | 2.83e-05 | 0.00307 | Wikipathways |
| Scavenging by Class A Receptors | 19 | 7 (36.8%) | 3.23e-05 | 0.00307 | Reactome |
| miR-targeted genes in muscle cell - TarBase | 400 | 41 (10.2%) | 3.62e-05 | 0.00307 | Wikipathways |
| Collagen chain trimerization | 44 | 10 (22.7%) | 7.72e-05 | 0.00581 | Reactome |
| Collagen formation | 92 | 15 (16.3%) | 8.87e-05 | 0.00601 | Reactome |
| Insulin Signaling | 160 | 21 (13.1%) | 0.000105 | 0.00624 | Wikipathways |
| Type II diabetes mellitus - Homo sapiens (human) | 46 | 10 (21.7%) | 0.000115 | 0.00624 | KEGG |
| EGF-Core | 105 | 16 (15.2%) | 0.00012 | 0.00624 | Signalink |
| Collagen biosynthesis and modifying enzymes | 68 | 12 (17.6%) | 0.000206 | 0.00968 | Reactome |
| SHP2 signaling | 59 | 11 (18.6%) | 0.000228 | 0.00968 | PID |
| Bladder cancer - Homo sapiens (human) | 41 | 9 (22.0%) | 0.000233 | 0.00968 | KEGG |
| PI3K-Akt Signaling Pathway | 340 | 34 (10.0%) | 0.00025 | 0.00968 | Wikipathways |
| MAPK Signaling Pathway | 246 | 27 (11.0%) | 0.000257 | 0.00968 | Wikipathways |
| Prolactin signaling pathway - Homo sapiens (human) | 70 | 12 (17.1%) | 0.000273 | 0.00974 | KEGG |
| **let-7c-3p** | | | | | |
| TGF-Ncore | 45 | 18 (40.0%) | 2.05e-09 | 2.24e-06 | Signalink |
| Gene expression (Transcription) | 1373 | 163 (12.0%) | 5.39e-09 | 2.95e-06 | Reactome |
| RNA Polymerase II Transcription | 1236 | 146 (11.9%) | 5.47e-08 | 1.99e-05 | Reactome |
| Pathways Affected in Adenoid Cystic Carcinoma | 63 | 19 (30.2%) | 1.56e-07 | 4.26e-05 | Wikipathways |
| Generic Transcription Pathway | 1107 | 131 (11.9%) | 2.54e-07 | 5.56e-05 | Reactome |
| cGMP-PKG signaling pathway - Homo sapiens (human) | 163 | 31 (19.0%) | 2.48e-06 | 0.000452 | KEGG |
| Mesodermal Commitment Pathway | 153 | 29 (19.0%) | 5.56e-06 | 0.000869 | Wikipathways |
| TGF-beta Signaling Pathway | 132 | 26 (19.7%) | 8.17e-06 | 0.00112 | Wikipathways |
| cAMP signaling pathway - Homo sapiens (human) | 199 | 34 (17.1%) | 9.75e-06 | 0.00119 | KEGG |
| Membrane Trafficking | 582 | 74 (12.7%) | 1.42e-05 | 0.00156 | Reactome |
| White fat cell differentiation | 32 | 11 (34.4%) | 1.64e-05 | 0.00163 | Wikipathways |
| Prion disease pathway | 33 | 11 (33.3%) | 2.28e-05 | 0.00208 | Wikipathways |
| Ectoderm Differentiation | 142 | 26 (18.3%) | 3.14e-05 | 0.00264 | Wikipathways |
| Intra-Golgi and retrograde Golgi-to-ER traffic | 186 | 31 (16.7%) | 3.91e-05 | 0.00296 | Reactome |
| Signaling by WNT | 278 | 41 (14.9%) | 4.06e-05 | 0.00296 | Reactome |
| Sterol Regulatory Element-Binding Proteins (SREBP) signalling | 68 | 16 (23.5%) | 4.78e-05 | 0.00308 | Wikipathways |
| Wnt | 69 | 16 (23.5%) | 4.78e-05 | 0.00308 | NetPath |
| Vesicle-mediated transport | 620 | 75 (12.1%) | 6.8e-05 | 0.00413 | Reactome |
| mRNA Processing | 127 | 23 (18.1%) | 0.000105 | 0.00597 | Wikipathways |
| Adrenergic signaling in cardiomyocytes - Homo sapiens (human) | 144 | 25 (17.4%) | 0.000109 | 0.00597 | KEGG |
| Signaling by TGF-beta family members | 96 | 19 (19.8%) | 0.000123 | 0.00602 | Reactome |
| Excitatory Neural Signalling Through 5-HTR 7 and Serotonin | 8 | 5 (62.5%) | 0.000128 | 0.00602 | SMPDB |
| RUNX1 interacts with co-factors whose precise effect on RUNX1 targets is not known | 40 | 11 (28.2%) | 0.000128 | 0.00602 | Reactome |
| carm1 and regulation of the estrogen receptor | 12 | 6 (50.0%) | 0.000132 | 0.00602 | BioCarta |
| TGF_beta_Receptor | 174 | 28 (16.2%) | 0.000153 | 0.0067 | NetPath |
| Adipogenesis | 131 | 23 (17.6%) | 0.000171 | 0.00717 | Wikipathways |
| Energy Metabolism | 47 | 12 (25.5%) | 0.000182 | 0.00739 | Wikipathways |
| G Protein Signaling Pathways | 92 | 18 (19.6%) | 0.000213 | 0.00834 | Wikipathways |
| TGF-beta signaling pathway - Homo sapiens (human) | 85 | 17 (20.0%) | 0.000241 | 0.00868 | KEGG |
| Wnt Signaling Pathway and Pluripotency | 101 | 19 (18.8%) | 0.000246 | 0.00868 | Wikipathways |
| Neural Crest Differentiation | 101 | 19 (18.8%) | 0.000246 | 0.00868 | Wikipathways |
| Endoderm Differentiation | 71 | 15 (21.1%) | 0.000295 | 0.00978 | Wikipathways |
| Glucocorticoid Receptor Pathway | 71 | 15 (21.1%) | 0.000295 | 0.00978 | Wikipathways |
| **let-7c-5p and let-7c-3p** | | | | | |
| Gene expression (Transcription) | 1373 | 238 (17.4%) | 2.85e-09 | 4.36e-06 | Reactome |
| miR-targeted genes in lymphocytes - TarBase | 489 | 103 (21.1%) | 1.69e-08 | 1.3e-05 | Wikipathways |
| Pathways Affected in Adenoid Cystic Carcinoma | 63 | 25 (39.7%) | 3.29e-08 | 1.41e-05 | Wikipathways |
| RNA Polymerase II Transcription | 1236 | 213 (17.3%) | 3.69e-08 | 1.41e-05 | Reactome |
| TGF-beta Signaling Pathway | 132 | 39 (29.5%) | 9.14e-08 | 2.79e-05 | Wikipathways |
| Generic Transcription Pathway | 1107 | 192 (17.5%) | 1.12e-07 | 2.86e-05 | Reactome |
| TGF-Ncore | 45 | 19 (42.2%) | 4.65e-07 | 0.000102 | Signalink |
| Mesodermal Commitment Pathway | 153 | 41 (26.8%) | 7.93e-07 | 0.000144 | Wikipathways |
| Axon guidance | 358 | 76 (21.3%) | 8.49e-07 | 0.000144 | Reactome |
| miR-targeted genes in muscle cell - TarBase | 400 | 82 (20.5%) | 1.58e-06 | 0.000242 | Wikipathways |
| TGF_beta_Receptor | 174 | 43 (24.9%) | 3.77e-06 | 0.000524 | NetPath |
| Transcriptional misregulation in cancer - Homo sapiens (human) | 186 | 44 (23.8%) | 9.93e-06 | 0.00127 | KEGG |
| Developmental Biology | 620 | 111 (17.9%) | 2,00E-05 | 0.00235 | Reactome |
| Wnt Signaling Pathway and Pluripotency | 101 | 28 (27.7%) | 2.17e-05 | 0.00237 | Wikipathways |
| cGMP-PKG signaling pathway - Homo sapiens (human) | 163 | 39 (23.9%) | 2.69e-05 | 0.00274 | KEGG |
| Adipogenesis | 131 | 33 (25.2%) | 3.66e-05 | 0.0035 | Wikipathways |
| Wnt | 69 | 21 (30.9%) | 4.02e-05 | 0.00353 | NetPath |
| AGE-RAGE signaling pathway in diabetic complications - Homo sapiens (human) | 99 | 27 (27.3%) | 4.16e-05 | 0.00353 | KEGG |
| Signaling pathways regulating pluripotency of stem cells - Homo sapiens (human) | 139 | 34 (24.5%) | 5.37e-05 | 0.00432 | KEGG |
| Pathways in cancer - Homo sapiens (human) | 526 | 95 (18.1%) | 5.99e-05 | 0.00458 | KEGG |
| Osteoblast Signaling | 14 | 8 (57.1%) | 7.74e-05 | 0.00545 | Wikipathways |
| Hippo signaling pathway - Homo sapiens (human) | 154 | 36 (23.5%) | 7.84e-05 | 0.00545 | KEGG |
| Ectoderm Differentiation | 142 | 34 (23.9%) | 8.48e-05 | 0.00564 | Wikipathways |
| SREBF and miR33 in cholesterol and lipid homeostasis | 18 | 9 (50.0%) | 0.000107 | 0.00684 | Wikipathways |
| Factors and pathways affecting insulin-like growth factor (IGF1)-Akt signaling | 30 | 12 (40.0%) | 0.000117 | 0.00715 | Wikipathways |
| Transcription factor regulation in adipogenesis | 22 | 10 (45.5%) | 0.000122 | 0.00716 | Wikipathways |
| Sterol Regulatory Element-Binding Proteins (SREBP) signalling | 68 | 20 (29.4%) | 0.00013 | 0.00732 | Wikipathways |
| Signaling by Receptor Tyrosine Kinases | 423 | 78 (18.4%) | 0.000136 | 0.00732 | Reactome |
| cAMP signaling pathway - Homo sapiens (human) | 199 | 43 (21.6%) | 0.000139 | 0.00732 | KEGG |
| Platelet Aggregation Inhibitor Pathway, Pharmacodynamics | 49 | 16 (32.7%) | 0.000158 | 0.00803 | PharmGKB |
| Sudden Infant Death Syndrome (SIDS) Susceptibility Pathways | 159 | 36 (22.6%) | 0.00018 | 0.00871 | Wikipathways |
| Focal Adhesion-PI3K-Akt-mTOR-signaling pathway | 302 | 59 (19.5%) | 0.000182 | 0.00871 | Wikipathways |
| E2F transcription factor network | 75 | 21 (28.0%) | 0.000193 | 0.00886 | PID |
| AP-1 transcription factor network | 70 | 20 (28.6%) | 0.000201 | 0.00886 | PID |
| FoxO family signaling | 50 | 16 (32.0%) | 0.000206 | 0.00886 | PID |
| Prostate cancer - Homo sapiens (human) | 97 | 25 (25.8%) | 0.000211 | 0.00886 | KEGG |
| FoxO signaling pathway - Homo sapiens (human) | 132 | 31 (23.7%) | 0.000214 | 0.00886 | KEGG |
| ***MIR99A*** | | | | | |
| **miR-99a-5p** | | | | | |
| MAPK Signaling Pathway | 246 | 5 (2.0%) | 0.000621 | 0.00559 | Wikipathways |
| MAPK signaling pathway - Homo sapiens (human) | 295 | 5 (1.7%) | 0.0014 | 0.00631 | KEGG |
| **miR-99a-3p** | | | | | |
| Prolactin signaling pathway - Homo sapiens (human) | 70 | 4 (5.7%) | 3.57e-05 | 0.000927 | KEGG |
| **miR-99a-5p and miR-99a-3p** | | | | | |
| MAPK Signaling Pathway | 246 | 7 (2.8%) | 0.00038 | 0.00551 | Wikipathways |
| ESC Pluripotency Pathways | 116 | 5 (4.3%) | 0.000424 | 0.00551 | Wikipathways |
