# Supplementary material for: Differential methylation of microRNA encoding genes may contribute to high myopia
Source: Front Genet. 2023 Jan 4;13:1089784. doi: 10.3389/fgene.2022.1089784 (PMC9847511; doi:10.3389/fgene.2022.1089784)
Supplement: Supplementary file 5 [file Table2.DOCX]

**Supplementary Table 2. Decreased methylation levels: the highest-ranked CG dinucleotides in promoter regions and gene body of miRNA encoding genes, with at least 5% difference between HM cases and controls in methylation level**

| **TargetID** | **Chromosome** | **MicroRNA gene** | **p-value** | **FDR p-value** | **Methylation level in HM cases [%]** | **Methylation level in controls [%]** | **Difference in methylation level [%]** | **Localization in gene** |
| --- | --- | --- | --- | --- | --- | --- | --- | --- |
| cg21913981 | 12 | *MIR1178* | 5.15x10^-30^ | 4.45x10^-24^ | 64.65 | 82.02 | -17.38 | TSS200 |
| cg18590130 | 11 | *MIRLET7A2* | 2.79x10^-30^ | 2.41x10^-24^ | 69.71 | 85.71 | -16.00 | TSS1500 |
| cg05365685 | 3 | *MIR885* | 2.90x10^-23^ | 2.51x10^-17^ | 72.92 | 86.06 | -13.14 | TSS200 |
| cg23221889 | 3 | *MIR548G* | 1.09x10^-14^ | 9.47x10^-9^ | 62.28 | 75.37 | -13.09 | gene body |
| cg09705232 | 6 | *MIR548H3* | 2.25x10^-10^ | 1.95x10^-4^ | 53.90 | 67.00 | -13.09 | gene body |
| cg10443315 | 8 | *MIR548I3* | 2.94x10^-19^ | 2.54x10^-13^ | 69.17 | 82.06 | -12.89 | TSS1500 |
| cg21281732 | 9 | *MIR6854* | 2.90x10^-27^ | 2.51x10^-21^ | 78.26 | 90.43 | -12.17 | TSS1500 |
| cg27116069 | 14 | *MIR548AZ* | 6.28x10^-18^ | 5.43x10^-12^ | 73.91 | 85.11 | -11.20 | gene body |
| cg13210239 | 11 | *MIR675* | 8.46x10^-11^ | 7.31x10^-5^ | 61.51 | 72.56 | -11.05 | TSS1500 |
| cg25353401 | 21 | *MIRLET7C;MIR99A* | 1.57x10^-13^ | 1.36x10^-7^ | 69.54 | 80.46 | -10.93 | TSS1500;TSS1500 |
| cg27364880 | 6 | *MIR587* | 1.72x10^-12^ | 1.48x10^-6^ | 73.63 | 83.23 | -9.60 | TSS200 |
| cg01866220 | 16 | *MIR4517* | 2.14x10^-9^ | 1.85x10^-3^ | 68.52 | 77.95 | -9.43 | TSS1500 |
| cg16094326 | 17 | *MIR548W* | 3.90x10^-11^ | 3.37x10^-5^ | 73.13 | 82.29 | -9.16 | gene body |
| cg04779243 | 16 | *MIR548AE2* | 1.78x10^-8^ | 1.54x10^-2^ | 70.11 | 78.89 | -8.78 | gene body |
| cg25013978 | 5 | *MIR580* | 2.60x10^-9^ | 2.25x10^-3^ | 71.05 | 79.81 | -8.77 | TSS1500 |
| cg00941833 | 2 | *MIR1302-4* | 9.26x10^-19^ | 8.01x10^-13^ | 83.39 | 92.12 | -8.73 | TSS1500 |
| cg23682214 | 2 | *MIR7853* | 6.38x10^-9^ | 5.52x10^-3^ | 67.12 | 75.78 | -8.66 | gene body |
| cg22351390 | 1 | *MIR548F1* | 1.32x10^-12^ | 1.15x10^-6^ | 77.18 | 85.72 | -8.54 | gene body |
| cg04185799 | 4 | *MIR548AJ2* | 1.13x10^-10^ | 9.74x10^-5^ | 68.14 | 76.42 | -8.29 | gene body |
| cg15968925 | 6 | *MIR548H3* | 1.93x10^-13^ | 1.67x10^-7^ | 83.54 | 90.90 | -7.36 | gene body |
| cg01464247 | 16 | *MIR5095* | 1.50x10^-8^ | 1.29x10^-2^ | 77.91 | 85.12 | -7.20 | gene body |
| cg14953687 | 9 | *MIR1268A* | 8.11x10^-13^ | 7.02x10^-7^ | 84.08 | 91.21 | -7.12 | gene body |
| cg04371579 | 3 | *MIR548G* | 5.91x10^-14^ | 5.11x10^-8^ | 84.65 | 91.74 | -7.09 | gene body |
| cg13924954 | 19 | *MIR516B2* | 4.70x10^-16^ | 4.06x10^-10^ | 86.75 | 93.81 | -7.06 | TSS1500 |
| cg18135087 | 1 | *MIR554* | 2.38x10^-13^ | 2.05x10^-7^ | 82.92 | 89.91 | -6.99 | TSS1500 |
| cg16879857 | 2 | *MIR217HG* | 1.00x10^-8^ | 8.67x10^-3^ | 77.33 | 84.14 | -6.81 | TSS200 |
| cg02373104 | 7 | *MIR548F3* | 2.70x10^-8^ | 2.33x10^-2^ | 78.89 | 85.69 | -6.81 | gene body |
| cg17777998 | 7 | *MIR1183* | 1.83x10^-8^ | 1.58x10^-2^ | 77.55 | 84.33 | -6.78 | TSS200 |
| cg00707427 | 14 | *MIR544A;MIR381HG;*  *MIR655* | 3.92x10^-8^ | 3.39x10^-2^ | 79.06 | 85.73 | -6.67 | TSS200;gene body; TSS1500 |
| cg22781400 | 19 | *MIR372;MIR371;MIR373* | 3.49x10^-12^ | 3.02x10^-6^ | 84.87 | 91.48 | -6.61 | TSS1500;TSS200;  TSS1500 |
| cg26492825 | 16 | *MIR548AE2* | 2.30x10^-9^ | 1.99x10^-3^ | 82.56 | 88.88 | -6.32 | gene body |
| cg06792448 | 14 | *MIR432;MIR136* | 2.75x10^-13^ | 2.38x10^-7^ | 87.17 | 93.38 | -6.22 | TSS1500;TSS1500 |
| cg00823526 | 1 | *MIR29B2;MIR29C* | 4.48x10^-10^ | 3.87x10^-4^ | 84.30 | 90.35 | -6.06 | TSS1500;TSS1500 |
| cg18485627 | 14 | *MIR665;MIR337* | 5.53x10^-12^ | 4.78x10^-6^ | 87.49 | 93.39 | -5.90 | TSS1500;TSS1500 |
| cg10511110 | 5 | *MIR548P* | 1.27x10^-9^ | 1.10x10^-3^ | 84.46 | 90.25 | -5.80 | TSS1500 |
| cg15207708 | 13 | *MIR548F5* | 1.33x10^-10^ | 1.15x10^-4^ | 83.04 | 88.82 | -5.78 | gene body |
| cg05425602 | 8 | *MIR30B* | 4.89x10^-8^ | 4.23x10^-2^ | 83.62 | 89.35 | -5.74 | TSS200 |
| cg25429902 | 7 | *MIR548F4;MIR548I4* | 8.10x10^-11^ | 7.00x10^-5^ | 82.55 | 88.25 | -5.71 | TSS1500;gene body |
| cg26223117 | 8 | *MIR7641-2* | 2.00x10^-11^ | 1.73x10^-5^ | 85.72 | 91.33 | -5.61 | TSS1500 |
| cg09525623 | 7 | *MIR1183* | 1.21x10^-10^ | 1.05x10^-4^ | 82.39 | 87.89 | -5.49 | TSS1500 |
| cg26475094 | 16 | *MIR138-2* | 6.29x10^-13^ | 5.44x10^-7^ | 85.53 | 90.90 | -5.37 | TSS1500 |
| cg05106421 | 2 | *MIR548N* | 3.20x10^-8^ | 2.77x10^-2^ | 85.09 | 90.28 | -5.19 | gene body |
| cg15272005 | 4 | *MIR1273H* | 5.44x10^-11^ | 4.70x10^-5^ | 86.88 | 92.06 | -5.17 | gene body |
| cg17933893 | 2 | *MIR548N* | 2.67x10^-8^ | 2.31x10^-2^ | 86.28 | 91.39 | -5.10 | gene body |
| cg16430726 | 13 | *MIR320D1* | 3.77x10^-8^ | 3.26x10^-2^ | 87.00 | 92.00 | -5.00 | TSS1500 |

FDR - false discovery rate, TSS200 - 0-200 bases upstream of the transcriptional start site, TSS1500 - 200-1500 bases upstream of the transcriptional start site,

* Methylation levels in children are presented as mean values
