# Supplementary material for: Differential methylation of microRNA encoding genes may contribute to high myopia
Source: Front Genet. 2023 Jan 4;13:1089784. doi: 10.3389/fgene.2022.1089784 (PMC9847511; doi:10.3389/fgene.2022.1089784)
Supplement: Supplementary file 6 [file Table5.docx]

**Supplementary Table 5. RNA sequencing data (Samuel et al., 2017) of ARPE-19 cell line for target genes of the highest-ranked miRNAs with CG dinucleotides with at least 10% higher methylation level in cases versus controls and localization within promoter region**

Values are presented as transcripts per million [TPM] reads, obtained from three replicates, in a 4 day culture

| **Gene name** | **Target score** | **4 days culture [TPM]** | | | |
| --- | --- | --- | --- | --- | --- |
|  |  | **rep1** | **rep2** | **rep3** | **mean** |
| **MIR3621** | | | | | |
| **miR-3621** | | | | | |
| *USP19* | 90 | 10.61 | 11.77 | 11.73 | **11.37** |
| *C4orf19* | 90 | 0.32 | 0.32 | 0.25 | **0.30** |
| **MIR34C** | | | | | |
| **miR-34c-5p** | | | | | |
| *FAM76A* | 100 | 3.46 | 3.54 | 3.31 | **3.44** |
| *DLL1* | 100 | 0.08 | 0.04 | 0.03 | **0.05** |
| *MDM4* | 100 | 11.24 | 13 | 11.81 | **12.02** |
| *HCN3* | 100 | 0.45 | 0.6 | 0.67 | **0.57** |
| *FAM167A* | 99 | 0.19 | 0.26 | 0.32 | **0.26** |
| *FKBP1B* | 99 | 6.46 | 5.65 | 6.25 | **6.12** |
| *SYT1* | 99 | 31.09 | 36.96 | 31.85 | **33.30** |
| *E2F5* | 99 | 12.06 | 13.34 | 12.08 | **12.49** |
| *RAP1GDS1* | 99 | 22.27 | 25.29 | 23.97 | **23.84** |
| *PPP1R11* | 99 | 30.29 | 30.97 | 30.67 | **30.64** |
| *SDK2* | 99 | 0.02 | 0 | 0.01 | **0.01** |
| *SATB2* | 99 | 1.48 | 1.63 | 2.04 | **1.72** |
| *MYCN* | 98 | 0 | 0.04 | 0 | **0.01** |
| *LGR4* | 98 | 13.71 | 15.85 | 14.75 | **14.77** |
| *FLOT2* | 98 | 11.24 | 11.7 | 11.94 | **11.63** |
| *CELF3* | 98 | 0 | 0 | 0.02 | **0.01** |
| *NAV1* | 98 | 2.56 | 2.97 | 3.05 | **2.86** |
| *MET* | 98 | 160.16 | 178.09 | 173.21 | **170.49** |
| *MGAT4A* | 98 | 2 | 2.14 | 2.22 | **2.12** |
| *NAV3* | 98 | 2.23 | 2.24 | 2.27 | **2.25** |
| *SCN2B* | 98 | 0.06 | 0.08 | 0.05 | **0.06** |
| *NECTIN1* | 98 | 0.74 | 0.76 | 0.7 | **0.73** |
| *AHCYL2* | 97 | 4.89 | 5.27 | 5.26 | **5.14** |
| *XYLT1* | 97 | 0.04 | 0.04 | 0.04 | **0.04** |
| *VAMP2* | 96 | 2.04 | 1.89 | 1.84 | **1.92** |
| *MPP2* | 96 | 3.15 | 2.99 | 2.72 | **2.95** |
| *PACS1* | 96 | 5.06 | 5.04 | 5.33 | **5.14** |
| *PKP4* | 96 | 89.88 | 94.45 | 97.37 | **93.90** |
| *CACNA1E* | 96 | 0 | 0 | 0 | **0.00** |
| *RRAS* | 96 | 27.66 | 26.85 | 25.71 | **26.74** |
| *TGIF2* | 96 | 10.03 | 9.78 | 10.01 | **9.94** |
| *MLLT3* | 96 | 9.89 | 11.85 | 11.94 | **11.23** |
| *PITPNC1* | 96 | 1.71 | 1.57 | 1.46 | **1.58** |
| *FUT9* | 96 | 0.06 | 0.05 | 0.08 | **0.06** |
| *FOXP1* | 95 | 8.5 | 10.53 | 9.82 | **9.62** |
| *SRPRA* | 95 | 25.55 | 27.41 | 26.62 | **26.53** |
| *CAMTA1* | 95 | 34.82 | 35 | 35.57 | **35.13** |
| *MEX3C* | 95 | 27.06 | 29.73 | 29.26 | **28.68** |
| *SLC25A27* | 95 | 3.67 | 4.03 | 3.55 | **3.75** |
| *ABR* | 95 | 28.32 | 30.14 | 30.86 | **29.77** |
| *NPNT* | 94 | 0.02 | 0 | 0 | **0.01** |
| *JAKMIP1* | 94 | 0 | 0 | 0 | **0.00** |
| *TOB2* | 94 | 6.91 | 8.2 | 7.6 | **7.57** |
| *ELMOD1* | 94 | 1.18 | 1.1 | 0.87 | **1.05** |
| *FUT8* | 94 | 11.13 | 12.93 | 12.65 | **12.24** |
| *SHANK3* | 94 | 0.09 | 0.11 | 0.19 | **0.13** |
| *LEF1* | 94 | 0.57 | 0.6 | 0.5 | **0.56** |
| *UBP1* | 93 | 19.02 | 20.9 | 19.8 | **19.91** |
| *GABRA3* | 93 | 0 | 0 | 0.01 | **0.00** |
| *DAAM1* | 93 | 12.13 | 14.55 | 14.77 | **13.82** |
| *CUEDC1* | 93 | 7.35 | 7.29 | 6.39 | **7.01** |
| *ZMYM4* | 93 | 25.34 | 28.84 | 26.23 | **26.80** |
| *ASIC2* | 93 | 0.64 | 0.67 | 0.59 | **0.63** |
| *BMP3* | 93 | 0.34 | 0.43 | 0.44 | **0.40** |
| *GALNT7* | 93 | 25.93 | 30.09 | 28.96 | **28.33** |
| *NUMBL* | 93 | 0.96 | 1.07 | 1.25 | **1.09** |
| *GPR22* | 93 | 0 | 0 | 0 | **0.00** |
| *KIAA1217* | 93 | 8.28 | 9.42 | 10.21 | **9.30** |
| *PPARGC1B* | 93 | 0.19 | 0.22 | 0.18 | **0.20** |
| *TBL1XR1* | 93 | 58.12 | 65.09 | 61.06 | **61.42** |
| *TNRC18* | 93 | 2.26 | 2.67 | 3.1 | **2.68** |
| *CDK6* | 92 | 80.16 | 102.01 | 95.78 | **92.65** |
| *EML5* | 92 | 1.87 | 2.14 | 1.76 | **1.92** |
| *SAR1A* | 92 | 100.62 | 108.29 | 103.11 | **104.01** |
| *TASOR* | 92 | 32.4 | 40.44 | 36.71 | **36.52** |
| *ATMIN* | 92 | 27.64 | 30.25 | 30.6 | **29.50** |
| *FGD6* | 92 | 3.55 | 3.58 | 3.46 | **3.53** |
| *FAM117B* | 92 | 2.86 | 3.58 | 3.07 | **3.17** |
| *CYREN* | 92 | 16.05 | 17.05 | 16.24 | **16.45** |
| *HNF4A* | 92 | 0 | 0 | 0 | **0.00** |
| *ARID4B* | 92 | 31.46 | 41.48 | 36.17 | **36.37** |
| *SFT2D1* | 92 | 41.07 | 46.49 | 43.43 | **43.66** |
| *TPPP* | 92 | 0.34 | 0.33 | 0.27 | **0.31** |
| *NRN1* | 92 | 0 | 0 | 0 | **0.00** |
| *FOXN2* | 92 | 8.51 | 10.5 | 9.86 | **9.62** |
| *TMEM255A* | 92 | 0 | 0 | 0 | **0.00** |
| *LMAN1* | 91 | 77.24 | 90.09 | 82.29 | **83.21** |
| *UNC13C* | 91 | 0 | 0 | 0 | **0.00** |
| *CTNND2* | 91 | 1.08 | 1.23 | 1.33 | **1.21** |
| *POGZ* | 91 | 8.65 | 10.02 | 9.56 | **9.41** |
| *ADO* | 91 | 12.95 | 15.95 | 14.8 | **14.57** |
| *PDE7B* | 91 | 1.54 | 1.75 | 1.74 | **1.68** |
| *SNAI1* | 91 | 0.04 | 0.07 | 0.03 | **0.05** |
| *KDM5D* | 91 | 8.08 | 9.23 | 8.91 | **8.74** |
| *ANK3* | 91 | 7.88 | 7.44 | 6.9 | **7.41** |
| *PPFIA1* | 90 | 19.33 | 21.65 | 20.66 | **20.55** |
| *GPR158* | 90 | 0.02 | 0.02 | 0.02 | **0.02** |
| *ADIPOR2* | 90 | 22.1 | 22.55 | 23.5 | **22.72** |
| *FGF23* | 90 | 0 | 0 | 0 | **0.00** |
| *AKIP1* | 90 | 18.32 | 18.88 | 18.72 | **18.64** |
| *CREB3L2* | 90 | 3.17 | 3.73 | 3.17 | **3.36** |
| *DNM1L* | 90 | 31.18 | 36.57 | 34.61 | **34.12** |
| *FOXJ2* | 90 | 1.56 | 1.68 | 1.91 | **1.72** |
| *TAF4B* | 90 | 3.28 | 3.5 | 3.58 | **3.45** |
| *PPP2R3A* | 90 | 14.27 | 16.57 | 15.11 | **15.32** |
| *CBX3* | 90 | 178.99 | 214.68 | 197.72 | **197.13** |
| *UCN2* | 90 | 0.17 | 0.08 | 0.04 | **0.10** |
| *SERPINF2* | 90 | 0.02 | 0.06 | 0.01 | **0.03** |
| *AMER1* | 90 | 1.02 | 1.25 | 1.02 | **1.10** |
| *RPS6KL1* | 90 | 2.53 | 2.74 | 2.58 | **2.62** |
| *SLC4A7* | 90 | 37.96 | 45.53 | 43.17 | **42.22** |
| *SMIM15* | 90 | 36.73 | 42.84 | 38.31 | **39.29** |
| *STRN3* | 90 | 21.59 | 22.84 | 22.28 | **22.24** |
| **miR-34c-3p** | | | | | |
| *NCKAP1* | 98 | 95.52 | 111.54 | 104.61 | **103.89** |
| *MAGI3* | 98 | 6.24 | 7.25 | 6.97 | **6.82** |
| *LYST* | 98 | 15.43 | 18.02 | 16.84 | **16.76** |
| *EIF4E* | 96 | 52.55 | 57.65 | 55.86 | **55.35** |
| *MARK1* | 94 | 3.07 | 3.42 | 3.18 | **3.22** |
| *TAF1B* | 94 | 21.34 | 23.14 | 22.36 | **22.28** |
| *KIF3A* | 93 | 9.87 | 12.38 | 10.7 | **10.98** |
| *DCBLD2* | 93 | 779.31 | 847.18 | 818.99 | **815.16** |
| *TIPARP* | 93 | 23.3 | 25.61 | 26.33 | **25.08** |
| *UGT2A3* | 93 | 0 | 0 | 0 | **0.00** |
| *CAPN1* | 92 | 13.73 | 14.9 | 15.42 | **14.68** |
| *PIH1D3* | 92 | 0.04 | 0.02 | 0.03 | **0.03** |
| *PRSS23* | 91 | 346.43 | 350.36 | 364.48 | **353.76** |
| *SVIP* | 91 | 15.9 | 19.89 | 19.03 | **18.27** |
| *APC* | 91 | 14.87 | 19.15 | 17.97 | **17.33** |
| *GPATCH8* | 91 | 4.42 | 5.6 | 5.16 | **5.06** |
| *AEBP2* | 90 | 8.87 | 11.15 | 10.21 | **10.08** |
| **MIR423** | | | | | |
| **miR-423-5p** | | | | | |
| *FAM222B* | 99 | 1.28 | 1.3 | 1.32 | **1.30** |
| *FOXP4* | 99 | 0.4 | 0.51 | 0.51 | **0.47** |
| *PLCB1* | 97 | 1.79 | 1.7 | 1.56 | **1.68** |
| *ADGRL1* | 97 | 0.63 | 0.55 | 0.65 | **0.61** |
| *C1QTNF6* | 96 | 2.29 | 2.2 | 2.31 | **2.27** |
| *EVC* | 96 | 18.19 | 18.85 | 19.48 | **18.84** |
| *PLA2G6* | 95 | 2.51 | 2.54 | 2.74 | **2.60** |
| *TMEM150A* | 94 | 3.5 | 3.9 | 4.16 | **3.85** |
| *SCN4A* | 94 | 0 | 0 | 0 | **0.00** |
| *TSPAN11* | 94 | 0.03 | 0.02 | 0.03 | **0.03** |
| *FRMD3* | 94 | 0.36 | 0.37 | 0.48 | **0.40** |
| *TRABD2B* | 93 | nd | nd | nd | **nd** |
| *CALM3* | 93 | 60.63 | 61.04 | 58.48 | **60.05** |
| *SLC20A2* | 93 | 13.99 | 15.04 | 14.56 | **14.53** |
| *ASB6* | 93 | 5.81 | 6.01 | 6.63 | **6.15** |
| *NAT8L* | 92 | 1.64 | 1.73 | 1.68 | **1.68** |
| *ST6GALNAC6* | 92 | 13.54 | 13.22 | 14.55 | **13.77** |
| *HIC2* | 92 | 0.45 | 0.41 | 0.53 | **0.46** |
| *PLEKHO1* | 92 | 30.6 | 31.95 | 32.56 | **31.70** |
| *RIMS4* | 92 | 0.13 | 0.11 | 0.11 | **0.12** |
| *MUL1* | 91 | 10.75 | 10.3 | 11.49 | **10.85** |
| *UBE2O* | 91 | 3.07 | 3.31 | 3.49 | **3.29** |
| *ODF3L1* | 91 | 0.06 | 0 | 0.05 | **0.04** |
| *NNAT* | 91 | 0 | 0 | 0 | **0.00** |
| *STK40* | 91 | 5.41 | 5.95 | 5.39 | **5.58** |
| *PGRMC2* | 91 | 41.06 | 47.84 | 44.54 | **44.48** |
| *NRSN2* | 91 | 6.31 | 6.49 | 6.67 | **6.49** |
| *SHANK1* | 91 | 0 | 0 | 0 | **0.00** |
| *CELSR2* | 90 | 0.65 | 0.71 | 0.89 | **0.75** |
| *RAD9B* | 90 | 0.47 | 0.39 | 0.47 | **0.44** |
| *SYP* | 90 | 0.33 | 0.3 | 0.4 | **0.34** |
| *DNALI1* | 90 | 5.86 | 5.54 | 5.83 | **5.74** |
| *NFIC* | 90 | 1.05 | 1.16 | 1.65 | **1.29** |
| *LZTS3* | 90 | 2.45 | 2.58 | 2.6 | **2.54** |
| *COL1A1* | 90 | 0.63 | 0.8 | 0.8 | **0.74** |
| *SUFU* | 90 | 0.83 | 0.82 | 0.92 | **0.86** |
| **miR-423-3p** | | | | | |
| *PABPC1* | 96 | 286.31 | 304.27 | 307.93 | **299.50** |

nd - no data
