# Supplementary material for: Differential methylation of microRNA encoding genes may contribute to high myopia
Source: Front Genet. 2023 Jan 4;13:1089784. doi: 10.3389/fgene.2022.1089784 (PMC9847511; doi:10.3389/fgene.2022.1089784)
Supplement: Supplementary file 8 [file Table7.PDF]

Supplementary Table 7. Characteristics of genes involved in the function or structure of the eye, which are targets of the highest-ranked miRNAs. Expression values are based on RNA sequencing results of the ARPE-19 cell line (TPM)(Samuel et al., 2017) and the Human Protein Atlas (RNA expression in retina and retinal cells)(nTPM). The table shows target genes with the highest and the lowest TPM value in a four-day culture of the ARPE-19 cell line.

Gene name	Target score	ARPE-19 cell line [TPM]	whole retina [nTPM]	bipolar cells [nTPM]	horizontal cells [nTPM]	muller glia cells [nTPM]	endothelial cells [nTPM]	cone photoreceptor cells [nTPM]	rod photoreceptor cells [nTPM]	Coding protein	*Locus*	Myopia *locus*	Associations with eye related phenotypes (GWAS Catalog)	Mouse ocular phenotype (Mouse Genome Informatics)	Aspects involved in myopia
**A. Target genes of the highest-ranked miRNAs with the highest expression level in ARPE-19 cell line**															
DCBLD2	93	815.16	22.4	20.6	13.5	73.3	21.8	77.2	55.1	discoidin, CUB and LCCL domain containing 2	3q12.1; 3			Mutation in this gene causes abnormal retinal vasculature morphology (MGI:1920629)	
NAP1L1	98	733.22	199.8	198.7	252.4	461.3	203.9	79.2	177.5	nucleosome assembly protein 1 like 1	12q21.2	12q21-q23 (MYP3)			
EIF4B	95	609.71	114.5	98.1	63.9	163.6	112.5	31.3	114.2	eukaryotic translation initiation factor 4B	12q13.13	12q13 (MYP24)	Low myopia vs hyperopia in Europeans (GCST012400)		
HSPD1	98	391.44	71.8	100.8	95.9	167.5	137.6	265.5	150.9	heat shock protein family D (Hsp60) member 1	2q33.1				
PRSS23	91	353.76	2.7	5.3	0	1.0	200.6	2.1	1.9	serine protease 23	11q14.2		Intraocular pressure in Europeans (GCST010376, GCST006412, GCST006394), glaucoma in Europeans (GCST006395)		

ITGAV	93	299.11	80.4	169.4	15.2	93.9	30.7	77.2	72.0	integrin subunit alpha V	2q32.1				
SUMO2	95	286.72	205.9	277.5	233.9	749.9	297.7	50.1	300.6	small ubiquitin like modifier 2	17q25.1				
CAPN2	96	255.84	19.2	17.2	13.5	28.1	62.3	33.3	23.7	calpain 2	1q41				
GNAS	96	252.62	731.2	456.3	1886.1	1833.3	750.1	56.3	394.7	GNAS complex locus	20q13.3 2		High myopia in Europeans (GCST012403)	Mutation in this gene causes ocular hypertelorism (MGI:95777)	
TRAM1	90	243.24	33.3	25.2	23.5	72.7	86.6	14.6	65.2	translocation associated membrane protein 1	8q13.3		Myopia in Europeans (GCST90134549), Refractive error in Europeans (GCST010002), Spherical equivalent or myopia (age of diagnosis) in multiethnic population (GCST006291), Spherical equivalent in Europeans (GCST010378)		
PLS3	96	236.08	11.9	0.7	0	30.7	65.6	0	3.2	plastin 3	Xq23	Xq23-q27 (MYP13)			
CTNNB1	97	212.84	88.8	48.8	13.5	121.7	199.8	33.3	79.5	catenin beta 1	3p22.1		Myopia in Europeans (GCST90134549), Refractive error in Europeans (GCST010002), Retinal vascular fractal dimension in Europeans (GCST90095200), Retinal vascular fractal	Mutations in this gene cause abnormal morphology of the eye (retina, lens) and disorders e. g. cataract, microphthalmia (MGI:88276)	(Wan et al., 2018; Liu and Li, 2022)

													density in Europeans (GCST90095201)		
BZW1	92	212.27	45.8	42.4	58.9	128.3	109.2	18.8	36.5	basic leucine zipper and W2 domains 1	2q33.1			Mutation in this gene causes cataract and persistence of hyaloid vascular system (MGI:1914132)	
ACTN4	96	206.92	26.0	42.6	23.5	172.7	205.5	14.6	39.4	actinin alpha 4	19q13.2		Retinal venular tortuosity in Europeans (GCST009422)	Mutations in this gene cause abnormal morphology of retina, eye muscle, lens epithelium, and optic cup (MGI:1890773)	
CBX3	90	197.13	122.7	127.7	50.5	169.9	68.8	77.2	149.6	chromobox 3	7p15.2	7p15 (MYP17)			
LIMCH1	97	192.36	35.7	50.3	0	135.7	130.2	37.5	90.5	LIM and calponin homology domains 1	4p13		Optic disc size in Europeans (GCST009462), Macular thickness in Europeans (GCST006976)	Mutation in this gene causes abnormal retina morphology (MGI:1924819)	
MRFAP1	91	187.92	165.7	147.1	111.0	230.8	120.6	16.7	208.9	Morf4 family associated protein 1	4p16.1	4p16 (MYP23)			
MET	98	170.49	3.9	13.7	0	9.0	0	0	0.4	MET proto-oncogene, receptor tyrosine kinase	7q31.2				(Khor et al., 2009; Yang et al., 2014)
SRSF3	94	145.26	45.2	163.8	99.3	439.5	314.0	54.2	243.4	serine and arginine rich splicing factor 3	6p21.31-p21.2		Glaucoma in multiethnic populations (GCST90011770, GCST90011768,		

													GCST90018852, GCST011438, GCST011439)
**B. Target genes of the highest-ranked miRNAs with the lowest expression level in ARPE-19 cell line**													
PRTG	100	5.01	10.4	22.2	0	6.2	14.6	191.8	92.6	protogenin	15q21.3		Corneal resistance factor in Europeans (GCST90102517), Intraocular pressure in Europeans (GCST005580)
ZMIZ1	96	5.04	14.6	70.5	63.9	16.4	23.5	0	4.8	zinc finger MIZ-type containing 1	10q22.3		Hyperopia in Europeans (GCST012401), Iris heterochromicity in East Asians (GCST005095)
GPATCH8	91	5.06	30.1	106.6	146.4	64.9	44.5	123.0	186.6	G-patch domain containing 8	17q21.31	17q21-q22 (MYP5)	
TENM3	98	5.09	3.9	87.7	5.0	13.4	0	4.2	3.8	teneurin transmembrane protein 3	4q34.3-q35.1	4q35 (MYP22)	Refractive error in Europeans (GCST010002), DNA methylation variation (age effect) (GCST006660)
BAZ2A	90	5.1	48.8	80.4	32.0	34.3	38.0	137.6	85.4	bromodomain adjacent to zinc finger domain 2A	12q13.3	12q13 (MYP24)	
ATAD2B	96	5.11	9.4	30.1	10.1	18.9	17.8	47.9	70.1	ATPase family AAA domain containing 2B	2p24.1-p23.3		Macular thickness in Europeans (GCST006976)
ATXN1	96	5.12	13.9	43.9	38.7	26.1	29.9	100.1	134.4	ataxin 1	6p22.3		Ccentral corneal thickness in Europeans (GCST90102518), Corneal resistance factor in Europeans (GCST90102517)

PACS1	96	5.14	35.1	56.9	92.5	17.4	6.5	50.1	47.3	phosphofurin acidic cluster sorting protein 1	11q13.1-q13.2		
AMMECR1	99	5.32	14.5	58.5	1.7	10.0	4.1	52.1	121.5	AMMECR nuclear protein 1	Xq23	Xq23-q27 (MYP13)	
JAZF1	91	5.4	7.8	14.4	16.8	4.9	2.4	6.2	7.1	JAZF zinc finger 1	7p15.2-p15.1	7p15 (MYP17)	Eye color in Europeans (GCST012219)	
PIM3	94	5.48	3.1	40.2	62.2	16.6	66.3	2.1	24.3	Pim-3 proto-oncogene, serine/threonine kinase	22q13.33	22q13 (MYP6)		
DNA2	93	5.58	5.3	2.8	0	1.2	0.8	6.2	7.9	DNA replication helicase/nuclease 2	10q21.3	10q21.1 (MYP15)		
GTPBP1	93	5.59	34.7	44.0	20.2	17.9	12.2	22.9	46.1	GTP binding protein 1	22q13.1	22q13 (MYP6)		
VAV2	93	5.73	3.0	1.0	0	6.3	2.4	0	3.5	vav guanine nucleotide exchange factor 2	9q34.2		Corneal astigmatism in Europeans (GCST007159), Corneal resistance factor in Europeans (GCST90100568), Central corneal thickness in Europeans (GCST006366)	Mutation in this gene causes abnormal iridocorneal angle, anterior iris synechia, ocular hypertension (MGI:102718)
DNALI1	90	5.74	11.8	4.1	3.3	22.1	4.8	8.4	20.8	dynein axonemal light intermediate chain 1	1p34.3		Intraocular pressure in Europeans (GCST005580)	
RUNX1	93	5.8	0.3	7.3	0	0.6	17.8	0	1.5	RUNX family transcription factor 1	21q22.12		Refractive error in Europeans (GCST010002), Corneal curvature in Europeans	

													(GCST001680), DNA methylation variation (age effect) (GCST006660)	
ENKD1	96	5.84	8.5	4.8	3.3	11.4	9.7	4.2	13.1	enkurin domain containing 1	16q22.1			Mutation in this gene causes abnormal photoreceptor connecting cilium morphology, decreased a-wave and b-wave amplitude, abnormal visual evoked potential, abnormal vision (MGI:2142593)
ITGB3	92	5.95	0.9	0	0	0	4.1	0	0	integrin subunit beta 3	17q21.32	17q21-q22 (MYP5)	Glaucoma (primary open-angle) in South Asians (Indians)(GCST003446)	Mutation in this gene causes vision/eye phenotype, abnormal induced retina neovascularization (MGI:96612)
DDI2	91	5.99	10.2	30.2	35.3	12.2	5.7	14.6	66.4	DNA damage inducible 1 homolog 2	1p36.21	1p36 (MYP14)		
FKBP1B	99	6.12	7.7	3.5	1.7	13.1	9.7	0	8.3	FKBP prolyl isomerase 1B	2p23.3			Mutation in this gene causes abnormal retina blood vessel (MGI:1336205)
HECTD2	91	6.13	11.7	24.8	28.6	13.8	12.9	27.1	9.3	HECT domain E3 ubiquitin protein ligase 2	10q23.32			

References:

Khor, C. C., Grignani, R., Ng, D. P. K., Toh, K. Y., Chia, K.-S., Tan, D., et al. (2009). cMET and refractive error progression in children. Ophthalmology 116, 1469–1474, 1474.e1. doi: 10.1016/j.ophtha.2009.02.026.

Liu, S., and Li, F. (2022). Analysis of Multifactor-Driven Myopia Disease Modules to Guide Personalized Treatment and Drug Development. Comput. Math. Methods Med. 2022, 5262259. doi: 10.1155/2022/5262259.

Wan, L., Deng, B., Wu, Z., and Chen, X. (2018). Exome sequencing study of 20 patients with high myopia. PeerJ 6, e5552. doi: 10.7717/peerj.5552.

Yang, X., Liu, X., Peng, J., Zheng, H., Lu, F., Gong, B., et al. (2014). Evaluation of MYOC, ACAN, HGF, and MET as candidate genes for high myopia in a Han Chinese population. Genet. Test. Mol. Biomark. 18, 446–452. doi: 10.1089/gtmb.2013.0479.
