# Supplementary material for: Differential methylation of microRNA encoding genes may contribute to high myopia
Source: Front Genet. 2023 Jan 4;13:1089784. doi: 10.3389/fgene.2022.1089784 (PMC9847511; doi:10.3389/fgene.2022.1089784)
Supplement: Supplementary file 9 [file Table6.docx]

**Supplementary Table 6. RNA sequencing data (Samuel et al., 2017) of ARPE-19 cell line for target genes of the highest-ranked miRNAs with CG dinucleotides with at least 10% lower methylation level in cases versus controls and localization within promoter region**

Values are presented as transcripts per million [TPM] reads, obtained from three replicates, in a 4 day culture

| ***Gene name*** | **Target score** | **4 days** | | | |
| --- | --- | --- | --- | --- | --- |
|  |  | **rep1** | **rep2** | **rep3** | **mean** |
| ***MIR1178*** | | | | | |
| **miR-1178-5p** | | | | | |
| *ELP4* | 96 | 16.95 | 18.59 | 17.05 | **17.53** |
| *ARL10* | 93 | 3.09 | 3.34 | 3.25 | **3.23** |
| *TFAP2B* | 92 | 0 | 0 | 0 | **0.00** |
| *FGF9* | 91 | 0.43 | 0.44 | 0.42 | **0.43** |
| *WARS* | 90 | 37.9 | 41.37 | 38.84 | **39.37** |
| *ERCC4* | 90 | 8.89 | 10.08 | 9.28 | **9.42** |
| *RASSF8* | 90 | 108.98 | 132.57 | 120.16 | **120.57** |
| *GTF3C3* | 90 | 26.63 | 29.72 | 26.77 | **27.71** |
| **miR-1178-3p** | | | | | |
| *AMMECR1* | 99 | 4.7 | 5.72 | 5.54 | **5.32** |
| *DNAH6* | 97 | 0.21 | 0.18 | 0.2 | **0.20** |
| *SLC39A6* | 96 | 44.16 | 46.76 | 46.1 | **45.67** |
| *GNAS* | 96 | 252.61 | 252.42 | 252.82 | **252.62** |
| *GLS* | 96 | 123.58 | 150.67 | 137.15 | **137.13** |
| *ATP1B1* | 95 | 121.59 | 134.43 | 120.63 | **125.55** |
| *ZMAT4* | 94 | 0 | 0.01 | 0 | **0.00** |
| *GPM6A* | 94 | 8.57 | 9.19 | 9.12 | **8.96** |
| *LEMD3* | 94 | 13.26 | 15.17 | 14.72 | **14.38** |
| *NPTN* | 94 | 73.27 | 80.81 | 79.57 | **77.88** |
| *SPAG9* | 94 | 56.71 | 64.15 | 59.62 | **60.16** |
| *ZZZ3* | 94 | 36.57 | 41.47 | 40.24 | **39.43** |
| *SNCA* | 93 | 15.13 | 14.8 | 16.24 | **15.39** |
| *FAM199X* | 93 | 22.84 | 27.08 | 24.06 | **24.66** |
| *ZNF385A* | 93 | 0.49 | 0.52 | 0.37 | **0.46** |
| *EIF1B* | 93 | 23.19 | 27.04 | 23.53 | **24.59** |
| *MSL3* | 93 | 14.14 | 15.68 | 14.23 | **14.68** |
| *FAM98A* | 92 | 21.02 | 22.69 | 21.54 | **21.75** |
| *RIMBP2* | 92 | 0 | 0 | 0 | **0.00** |
| *PPP3CB* | 92 | 15.69 | 17.06 | 15.67 | **16.14** |
| *AFF4* | 92 | 35.47 | 42.43 | 40.02 | **39.31** |
| *TMPRSS11E* | 92 | 0 | 0 | 0 | **0.00** |
| *ZNF888* | 91 | nd | nd | nd |  |
| *BCL2* | 91 | 1.79 | 2.57 | 2.39 | **2.25** |
| *SPTLC1* | 91 | 59 | 63.24 | 58.46 | **60.23** |
| *CDH13* | 91 | 1.52 | 1.78 | 1.65 | **1.65** |
| *STMN2* | 90 | 0 | 0 | 0 | **0.00** |
| *CDK8* | 90 | 9.67 | 10.95 | 9.82 | **10.15** |
| *AADAC* | 90 | 0 | 0 | 0 | **0.00** |
| *SRPRA* | 90 | 25.55 | 27.41 | 26.62 | **26.53** |
| *ZXDC* | 90 | 7.53 | 8.43 | 8.04 | **8.00** |
| ***MIRLET7A2*** | | | | | |
| **let-7a-5p** | | | | | |
| *FIGNL2* | 100 | 0 | 0 | 0.01 | **0.00** |
| *HMGA2* | 100 | 3.8 | 5.34 | 5.03 | **4.72** |
| *IGF2BP1* | 100 | 0.02 | 0.02 | 0.03 | **0.02** |
| *LIN28B* | 100 | 0 | 0 | 0 | **0.00** |
| *TRIM71* | 100 | 0.06 | 0.03 | 0.01 | **0.03** |
| *NR6A1* | 100 | 0.17 | 0.09 | 0.09 | **0.12** |
| *STARD13* | 100 | 17.48 | 19.73 | 19.34 | **18.85** |
| *IGDCC3* | 100 | 0.06 | 0.1 | 0.1 | **0.09** |
| *PRTG* | 100 | 4.8 | 5.06 | 5.17 | **5.01** |
| *C14orf28* | 100 | 4.67 | 4.79 | 4.32 | **4.59** |
| *ARID3B* | 100 | 0.73 | 0.95 | 0.79 | **0.82** |
| *FRMD4B* | 99 | 10.38 | 12.12 | 11.01 | **11.17** |
| *SMARCAD1* | 99 | 33.38 | 42.41 | 40.41 | **38.73** |
| *NPHP3* | 99 | 12.05 | 13.32 | 12.74 | **12.70** |
| *PTAFR* | 99 | 0.3 | 0.35 | 0.43 | **0.36** |
| *GATM* | 99 | 3.18 | 3.23 | 3.42 | **3.28** |
| *FIGN* | 99 | 0.53 | 0.81 | 0.7 | **0.68** |
| *HIC2* | 98 | 0.45 | 0.41 | 0.53 | **0.46** |
| *CBX5* | 98 | 36.65 | 41.16 | 37.76 | **38.52** |
| *PGRMC1* | 98 | 85 | 96.45 | 89.11 | **90.19** |
| *NAP1L1* | 98 | 707.49 | 781.91 | 710.25 | **733.22** |
| *CCND2* | 98 | 2.02 | 2.11 | 1.97 | **2.03** |
| *ZNF512B* | 97 | 4.78 | 5.04 | 5.09 | **4.97** |
| *MIB1* | 97 | 26.8 | 31.65 | 29.21 | **29.22** |
| *SLC10A7* | 97 | 2.5 | 3.07 | 3.06 | **2.88** |
| *CDC34* | 97 | 17.01 | 19.1 | 18.81 | **18.31** |
| *PAPPA* | 97 | 0.05 | 0.04 | 0.04 | **0.04** |
| *SALL4* | 97 | 0.01 | 0.05 | 0.02 | **0.03** |
| *LRIG3* | 97 | 6.7 | 7.41 | 7.01 | **7.04** |
| *NME6* | 97 | 9.33 | 10.79 | 10.21 | **10.11** |
| *SFMBT1* | 97 | 3.24 | 3.87 | 4.06 | **3.72** |
| *SLF2* | 97 | 16.41 | 18.42 | 17.2 | **17.34** |
| *ADAMTS15* | 97 | 0.96 | 1.11 | 1.31 | **1.13** |
| *ZBTB5* | 96 | 7.41 | 7.52 | 6.94 | **7.29** |
| *ZNF710* | 96 | 0.85 | 0.9 | 0.97 | **0.91** |
| *UTRN* | 96 | 40.93 | 50.24 | 48.5 | **46.56** |
| *STX3* | 96 | 54.48 | 58.46 | 57.54 | **56.83** |
| *SKIL* | 96 | 33.18 | 42.18 | 39.22 | **38.19** |
| *PIK3IP1* | 96 | 24.23 | 23.37 | 23.25 | **23.62** |
| *PPP1R15B* | 96 | 23.85 | 27.26 | 25.95 | **25.69** |
| *GXYLT1* | 96 | 7.03 | 8.34 | 8.11 | **7.83** |
| *GNG5* | 96 | 51.77 | 59.47 | 55.57 | **55.60** |
| *LPGAT1* | 96 | 10.97 | 13.1 | 12.91 | **12.33** |
| *YOD1* | 96 | 25.37 | 29.62 | 27.57 | **27.52** |
| *ZSWIM5* | 96 | 0.04 | 0.06 | 0.04 | **0.05** |
| *IGF1R* | 96 | 8.53 | 10.98 | 11.43 | **10.31** |
| *USP44* | 96 | 5.91 | 6.88 | 6.08 | **6.29** |
| *FZD3* | 96 | 2.23 | 2.49 | 2.43 | **2.38** |
| *CCNJ* | 96 | 8.48 | 9.72 | 8.36 | **8.85** |
| *DLC1* | 96 | 19.65 | 23.17 | 22.96 | **21.93** |
| *NRAS* | 96 | 43.34 | 47.83 | 44.34 | **45.17** |
| *BACH1* | 96 | 31.23 | 36.65 | 33.95 | **33.94** |
| *COIL* | 95 | 15.35 | 15.81 | 15.33 | **15.50** |
| *SMC1A* | 95 | 12.7 | 14.26 | 13.82 | **13.59** |
| *C8orf58* | 95 | 6.63 | 6.56 | 7.06 | **6.75** |
| *PXT1* | 95 | 0 | 0 | 0.05 | **0.02** |
| *CLCN5* | 95 | 1.49 | 1.62 | 1.69 | **1.60** |
| *THRSP* | 95 | 0 | 0 | 0.02 | **0.01** |
| *COL3A1* | 95 | 0.03 | 0.02 | 0 | **0.02** |
| *TET3* | 95 | 1.32 | 1.42 | 1.39 | **1.38** |
| *SMIM3* | 95 | 4.97 | 4.74 | 4.59 | **4.77** |
| *AGO4* | 95 | 8.88 | 9.13 | 9.05 | **9.02** |
| *CLDN12* | 95 | 27.08 | 30.78 | 28.18 | **28.68** |
| *BIN3* | 95 | 8.55 | 8.65 | 8.39 | **8.53** |
| *NIPAL4* | 95 | 4.57 | 5.49 | 5.16 | **5.07** |
| *ZNF644* | 95 | 34.51 | 45.54 | 41.97 | **40.67** |
| *GNPTAB* | 95 | 73.65 | 80.8 | 74.76 | **76.40** |
| *ADAMTS8* | 95 | 0 | 0.01 | 0.02 | **0.01** |
| *ADRB2* | 95 | 12.68 | 12.41 | 11.42 | **12.17** |
| *E2F5* | 94 | 12.06 | 13.34 | 12.08 | **12.49** |
| *ATP8B4* | 94 | 2.44 | 2.86 | 2.71 | **2.67** |
| *ADRB3* | 94 | 0 | 0 | 0 | **0.00** |
| *CPA4* | 94 | 45.09 | 48.63 | 49.22 | **47.65** |
| *IGF2BP3* | 94 | 6.95 | 8.24 | 7.91 | **7.70** |
| *STK40* | 94 | 5.41 | 5.95 | 5.39 | **5.58** |
| *DLST* | 94 | 37.2 | 36.42 | 36.1 | **36.57** |
| *MAP4K3* | 94 | 26.17 | 30.09 | 26.61 | **27.62** |
| *SLC16A9* | 94 | 0.02 | 0.1 | 0.1 | **0.07** |
| *C5orf51* | 94 | 8.96 | 10.26 | 9.58 | **9.60** |
| *TGFBR3* | 94 | 16.14 | 17.55 | 17.27 | **16.99** |
| *SLC35D2* | 94 | 21.02 | 23.06 | 21.91 | **22.00** |
| *HAND1* | 94 | 0 | 0 | 0 | **0.00** |
| *ONECUT2* | 94 | 0.16 | 0.18 | 0.19 | **0.18** |
| *DTX4* | 93 | 2.1 | 2.12 | 2.49 | **2.24** |
| *SENP2* | 93 | 16.85 | 17.63 | 17.04 | **17.17** |
| *EDN1* | 93 | 6.02 | 7.14 | 7.42 | **6.86** |
| *PLEKHA8* | 93 | 7.89 | 9.56 | 8.72 | **8.72** |
| *TMPRSS2* | 93 | 0 | 0 | 0 | **0.00** |
| *FRAS1* | 93 | 2.31 | 3.19 | 3.25 | **2.92** |
| *PTPRD* | 93 | 4.12 | 4.52 | 4.47 | **4.37** |
| *MARS2* | 93 | 4.38 | 4.9 | 4.95 | **4.74** |
| *FAM189A1* | 93 | 1.71 | 1.72 | 1.82 | **1.75** |
| *ARHGEF38* | 93 | 0.01 | 0.01 | 0 | **0.01** |
| *PEX11B* | 93 | 14.11 | 15.59 | 15.56 | **15.09** |
| *DNA2* | 93 | 4.99 | 6.13 | 5.62 | **5.58** |
| *AHCTF1* | 93 | 22.83 | 27.17 | 26.77 | **25.59** |
| *DMD* | 93 | 32.34 | 40.18 | 36.41 | **36.31** |
| *ZNF280B* | 93 | 1.91 | 1.72 | 1.81 | **1.81** |
| *GAS7* | 93 | 9.92 | 10.1 | 10.51 | **10.18** |
| *DTX2* | 93 | 1.13 | 1.11 | 1.28 | **1.17** |
| *BZW1* | 92 | 201.92 | 226.46 | 208.43 | **212.27** |
| *CEMIP2* | 92 | 15.57 | 17.78 | 17.36 | **16.90** |
| *TSEN34* | 92 | 19.6 | 19.61 | 19.44 | **19.55** |
| *USP38* | 92 | 17.72 | 19.11 | 18.91 | **18.58** |
| *ITGB3* | 92 | 5.55 | 6.08 | 6.21 | **5.95** |
| *WNT9B* | 92 | 0.29 | 0.43 | 0.31 | **0.34** |
| *CPEB2* | 92 | 6.23 | 7.09 | 6.44 | **6.59** |
| *ZBP1* | 92 | 0.07 | 0.05 | 0.08 | **0.07** |
| *PCGF3* | 92 | 16.42 | 16.6 | 16.14 | **16.39** |
| *COL1A2* | 92 | 0 | 0 | 0 | **0.00** |
| *HAS2* | 92 | 0 | 0 | 0 | **0.00** |
| *LAMP2* | 92 | 115.84 | 128.44 | 121.56 | **121.95** |
| *CLP1* | 92 | 11.11 | 12.06 | 10.73 | **11.30** |
| *CPEB1* | 92 | 0.28 | 0.39 | 0.3 | **0.32** |
| *PBX2* | 92 | 8.54 | 9.05 | 8.99 | **8.86** |
| *TGFBR1* | 92 | 31.16 | 33.64 | 30.87 | **31.89** |
| *TTLL4* | 92 | 3.18 | 3.65 | 3.73 | **3.52** |
| *PXDN* | 92 | 34.09 | 40.25 | 43.31 | **39.22** |
| *ZNF322* | 92 | 23.66 | 25.2 | 24.71 | **24.52** |
| *NHLRC3* | 92 | 11.5 | 12.65 | 11.89 | **12.01** |
| *RASGRP1* | 92 | 5.71 | 7.04 | 6.95 | **6.57** |
| *TMEM65* | 92 | 13.56 | 15.16 | 13.78 | **14.17** |
| *RUFY3* | 92 | 36.37 | 45.17 | 41.88 | **41.14** |
| *MDM4* | 92 | 11.24 | 13 | 11.81 | **12.02** |
| *COL4A2* | 92 | 34.37 | 40.47 | 43 | **39.28** |
| *TRIM67* | 92 | 0 | 0 | 0 | **0.00** |
| *GDF6* | 92 | 0.25 | 0.24 | 0.25 | **0.25** |
| *AMT* | 91 | 0.68 | 0.84 | 0.72 | **0.75** |
| *SLC5A9* | 91 | 0 | 0 | 0 | **0.00** |
| *MAPK6* | 91 | 58.95 | 64.85 | 59.31 | **61.04** |
| *ZFYVE26* | 91 | 7.95 | 8.61 | 8.57 | **8.38** |
| *IGDCC4* | 91 | 0.13 | 0.12 | 0.17 | **0.14** |
| *E2F6* | 91 | 9.29 | 9.85 | 9.65 | **9.60** |
| *FAM135A* | 91 | 8.72 | 10.85 | 10.45 | **10.01** |
| *B3GNT7* | 91 | 0.05 | 0.05 | 0.03 | **0.04** |
| *PBX1* | 91 | 3.61 | 4.35 | 3.85 | **3.94** |
| *FNIP1* | 91 | 22.67 | 25.92 | 23.55 | **24.05** |
| *ASAP1* | 91 | 8.94 | 9.76 | 9.28 | **9.33** |
| *ERCC6* | 91 | 16.63 | 18.66 | 17.43 | **17.57** |
| *ACVR1C* | 91 | 0.06 | 0.05 | 0.03 | **0.05** |
| *RGS16* | 91 | 0.14 | 0.14 | 0.16 | **0.15** |
| *DDI2* | 91 | 5.59 | 6.67 | 5.72 | **5.99** |
| *XRN1* | 91 | 22.59 | 26.94 | 25.22 | **24.92** |
| *PLXNC1* | 91 | 1.87 | 2.02 | 1.77 | **1.89** |
| *HECTD2* | 91 | 5.32 | 7.09 | 5.99 | **6.13** |
| *TMPPE* | 91 | 1.98 | 2.42 | 2.17 | **2.19** |
| *SOCS4* | 91 | 19.46 | 23.63 | 21.61 | **21.57** |
| *PBX3* | 91 | 54.16 | 60.25 | 55.4 | **56.60** |
| *CEP135* | 91 | 8.04 | 10.26 | 8.9 | **9.07** |
| *RBFOX2* | 91 | 26.64 | 29.35 | 27.12 | **27.70** |
| *PALD1* | 90 | 1.47 | 1.66 | 1.62 | **1.58** |
| *GALNT2* | 90 | 44.47 | 47.65 | 46.89 | **46.34** |
| *CARNMT1* | 90 | 13.17 | 14.44 | 14.69 | **14.10** |
| *IL13* | 90 | 0 | 0 | 0 | **0.00** |
| *PLXND1* | 90 | 2.72 | 2.7 | 2.74 | **2.72** |
| *SRGAP1* | 90 | 1.13 | 1.6 | 1.69 | **1.47** |
| *HIP1* | 90 | 4.39 | 5.21 | 5.06 | **4.89** |
| *THOC2* | 90 | 36.1 | 53.48 | 45.47 | **45.02** |
| *FAM122A* | 90 | 7.53 | 8.4 | 8.68 | **8.20** |
| *KCTD17* | 90 | 4.65 | 4.2 | 4.46 | **4.44** |
| *LIMD2* | 90 | 0.72 | 0.94 | 0.68 | **0.78** |
| *KLHDC8B* | 90 | 11.06 | 10.36 | 10.93 | **10.78** |
| *CD59* | 90 | 117.65 | 123.3 | 121.85 | **120.93** |
| *DPP6* | 90 | 0 | 0 | 0.01 | **0.00** |
| *DDX19B* | 90 | 14.55 | 16.46 | 15.44 | **15.48** |
| *GALC* | 90 | 20.75 | 22.15 | 21.11 | **21.34** |
| *KCTD21* | 90 | 3.45 | 2.77 | 3.32 | **3.18** |
| *BEGAIN* | 90 | 1.32 | 1.2 | 1.35 | **1.29** |
| *CNOT6L* | 90 | 5.68 | 6.19 | 5.96 | **5.94** |
| *HSPA14* | 90 | 24.54 | 28.8 | 27.25 | **26.86** |
| **let-7a-2-3p** | | | | | |
| *TANC2* | 100 | 0.84 | 0.93 | 0.85 | **0.87** |
| *BACH2* | 100 | 1.29 | 1.55 | 1.36 | **1.40** |
| *ARFGEF1* | 100 | 23.57 | 26.05 | 24.68 | **24.77** |
| *LRIG1* | 100 | 1.84 | 2.16 | 2.58 | **2.19** |
| *KCNJ6* | 99 | 0 | 0 | 0 | **0.00** |
| *STXBP5L* | 99 | 0 | 0 | 0 | **0.00** |
| *MBTD1* | 99 | 9.41 | 10.61 | 10.42 | **10.15** |
| *MBNL2* | 99 | 130.42 | 156.94 | 144.15 | **143.84** |
| *ABTB2* | 98 | 0.26 | 0.29 | 0.4 | **0.32** |
| *SP3* | 98 | 34.19 | 38.21 | 37.08 | **36.49** |
| *HAT1* | 98 | 47.88 | 53.93 | 52.16 | **51.32** |
| *VLDLR* | 98 | 9.3 | 9.38 | 9.86 | **9.51** |
| *TENM3* | 98 | 4.86 | 5 | 5.42 | **5.09** |
| *RBM27* | 98 | 25.03 | 27.41 | 27.56 | **26.67** |
| *PPP4R1* | 98 | 46.39 | 48.92 | 48.3 | **47.87** |
| *FLRT3* | 98 | 8.23 | 8.65 | 7.62 | **8.17** |
| *ATP13A3* | 98 | 327.17 | 376.22 | 355.23 | **352.87** |
| *DACH1* | 98 | 0.38 | 0.63 | 0.51 | **0.51** |
| *SHOC2* | 97 | 22.56 | 26.7 | 23.46 | **24.24** |
| *HMGN1* | 97 | 239.34 | 272.72 | 256.25 | **256.10** |
| *DSCAML1* | 97 | 0 | 0 | 0 | **0.00** |
| *MBD5* | 97 | 3.77 | 4.33 | 3.95 | **4.02** |
| *TAB2* | 97 | 19.92 | 22.09 | 21.4 | **21.14** |
| *SH2B3* | 96 | 2.19 | 2.5 | 2.52 | **2.40** |
| *RFX4* | 96 | 0.01 | 0.03 | 0.01 | **0.02** |
| *ATL2* | 96 | 16.85 | 19.53 | 18.7 | **18.36** |
| *CD83* | 96 | 1.49 | 1.48 | 1.4 | **1.46** |
| *CDYL* | 96 | 12.1 | 12.4 | 11.58 | **12.03** |
| *CCDC126* | 96 | 8.22 | 10.12 | 9.26 | **9.20** |
| *ANP32B* | 96 | 15.8 | 18.23 | 17.32 | **17.12** |
| *PGAP1* | 96 | 3.73 | 4.05 | 3.67 | **3.82** |
| *RAB5C* | 96 | 50.39 | 48.09 | 49.11 | **49.20** |
| *MAGI2* | 96 | 1.44 | 1.45 | 1.44 | **1.44** |
| *B3GNT9* | 96 | 3.59 | 4.05 | 4.19 | **3.94** |
| *FBXL3* | 96 | 34.86 | 42.5 | 39.19 | **38.85** |
| *NFKBIA* | 95 | 21.9 | 21.06 | 22.26 | **21.74** |
| *SUSD6* | 95 | 2.39 | 2.81 | 2.68 | **2.63** |
| *DCUN1D1* | 95 | 26.12 | 32.78 | 29.08 | **29.33** |
| *USP6NL* | 95 | 9.99 | 11.96 | 10.39 | **10.78** |
| *TBP* | 95 | 30.01 | 32.25 | 32.04 | **31.43** |
| *ANTXR1* | 95 | 46.81 | 52.21 | 48.81 | **49.28** |
| *TDO2* | 95 | 0.24 | 0.24 | 0.13 | **0.20** |
| *KDM3A* | 95 | 26.6 | 27.76 | 27.96 | **27.44** |
| *PPP1CC* | 95 | 122.23 | 137.25 | 128.06 | **129.18** |
| *MYT1L* | 95 | 0 | 0.01 | 0 | **0.00** |
| *TMEM165* | 95 | 70.68 | 82.52 | 76.95 | **76.72** |
| *HS6ST3* | 95 | 0.01 | 0.01 | 0.01 | **0.01** |
| *KIAA2026* | 95 | 6.39 | 8.8 | 7.51 | **7.57** |
| *SLC6A17* | 95 | 0.22 | 0.46 | 0.29 | **0.32** |
| *SLC20A2* | 95 | 13.99 | 15.04 | 14.56 | **14.53** |
| *THAP11* | 95 | 7.63 | 8.05 | 8.24 | **7.97** |
| *PLCB1* | 95 | 1.79 | 1.7 | 1.56 | **1.68** |
| *GABRA5* | 94 | 4.21 | 4.39 | 4.28 | **4.29** |
| *MAP4K3* | 94 | 26.17 | 30.09 | 26.61 | **27.62** |
| *NET1* | 94 | 12.31 | 12.9 | 13.22 | **12.81** |
| *RASL11B* | 94 | 0.4 | 0.41 | 0.45 | **0.42** |
| *AAK1* | 94 | 1.5 | 1.64 | 1.58 | **1.57** |
| *PCGF5* | 94 | 17 | 21.55 | 20.08 | **19.54** |
| *MEGF11* | 94 | 0.07 | 0.14 | 0.08 | **0.10** |
| *DYRK4* | 94 | 21.85 | 21.41 | 23.2 | **22.15** |
| *ZNF341* | 94 | 0.74 | 0.78 | 0.7 | **0.74** |
| *BAZ1A* | 94 | 19.64 | 24.97 | 22.87 | **22.49** |
| *ZNF143* | 94 | 10.93 | 11.45 | 11.67 | **11.35** |
| *CDH11* | 94 | 80.35 | 95.53 | 92.74 | **89.54** |
| *AKAP1* | 94 | 9 | 8.85 | 9.41 | **9.09** |
| *SNRPB2* | 94 | 58.41 | 63.22 | 61.71 | **61.11** |
| *ZEB1* | 94 | 8.62 | 10.09 | 9.38 | **9.36** |
| *FLI1* | 94 | 0.04 | 0.07 | 0.03 | **0.05** |
| *REV3L* | 93 | 23.89 | 31.54 | 28.27 | **27.90** |
| *TCF7L2* | 93 | 2.29 | 2.95 | 2.6 | **2.61** |
| *MSH3* | 93 | 12.44 | 14.62 | 12.63 | **13.23** |
| *FMR1* | 93 | 24.54 | 29.7 | 28.29 | **27.51** |
| *PAPSS2* | 93 | 129.53 | 147.75 | 137.41 | **138.23** |
| *CPEB3* | 93 | 0.5 | 0.53 | 0.51 | **0.51** |
| *VAV2* | 93 | 5.78 | 5.71 | 5.7 | **5.73** |
| *CACNA1D* | 93 | 0.63 | 0.73 | 0.67 | **0.68** |
| *RBBP5* | 92 | 13.93 | 14.63 | 14.07 | **14.21** |
| *MYCBP2* | 92 | 10.95 | 12.72 | 12.47 | **12.05** |
| *JMJD1C* | 92 | 36.66 | 48.75 | 44.02 | **43.14** |
| *CCSER1* | 92 | 0.51 | 0.52 | 0.64 | **0.56** |
| *BAP1* | 92 | 15.15 | 15.17 | 15.01 | **15.11** |
| *FEZF2* | 92 | 0 | 0 | 0 | **0.00** |
| *FBXO38* | 92 | 26.3 | 28.31 | 27.2 | **27.27** |
| *PPARGC1A* | 92 | 1.74 | 1.73 | 1.57 | **1.68** |
| *FAT1* | 92 | 135.87 | 147.01 | 151.65 | **144.84** |
| *TNFSF11* | 92 | 0 | 0 | 0 | **0.00** |
| *MEA1* | 92 | 74.43 | 71.86 | 72.5 | **72.93** |
| *BCL11B* | 92 | 0.21 | 0.23 | 0.23 | **0.22** |
| *HOXA1* | 92 | 0 | 0 | 0 | **0.00** |
| *IQCK* | 91 | 8.18 | 6.84 | 7.18 | **7.40** |
| *GDNF* | 91 | 0.06 | 0.06 | 0.05 | **0.06** |
| *MRFAP1* | 91 | 183.91 | 192.59 | 187.26 | **187.92** |
| *HPS1* | 91 | 14.93 | 13.95 | 14.17 | **14.35** |
| *NID1* | 91 | 1.86 | 2.46 | 2.02 | **2.11** |
| *BBC3* | 91 | 6.1 | 6.4 | 6.78 | **6.43** |
| *TMSB4Y* | 91 | 0.75 | 0.91 | 0.66 | **0.77** |
| *RAD21* | 91 | 59.74 | 65.79 | 62.31 | **62.61** |
| *BAG3* | 91 | 10.49 | 12.27 | 11.37 | **11.38** |
| *KCNK1* | 91 | 1.87 | 2.66 | 2.17 | **2.23** |
| *RTL8A* | 91 | 14.27 | 15.4 | 12.53 | **14.07** |
| *CUL1* | 90 | 34.87 | 37.62 | 36.42 | **36.30** |
| *PDE4D* | 90 | 0.21 | 0.25 | 0.22 | **0.23** |
| *FUBP1* | 90 | 82.02 | 84.76 | 81.02 | **82.60** |
| *RASGRF1* | 90 | 0.46 | 0.47 | 0.39 | **0.44** |
| *NAT8L* | 90 | 1.64 | 1.73 | 1.68 | **1.68** |
| *BCL6* | 90 | 9.64 | 9.9 | 9.64 | **9.73** |
| *LARP4* | 90 | 20.52 | 24.71 | 24.03 | **23.09** |
| *PDSS2* | 90 | 7.89 | 9.37 | 9.53 | **8.93** |
| *C1QBP* | 90 | 121.08 | 120.79 | 119.08 | **120.32** |
| *CCNG2* | 90 | 18.04 | 18.75 | 17.78 | **18.19** |
| *SMG1* | 90 | 14.36 | 16.64 | 16.53 | **15.84** |
| *HMGB2* | 90 | 122.19 | 126.14 | 127 | **125.11** |
| *MYO1E* | 90 | 72.62 | 77.13 | 72.82 | **74.19** |
| *MED13L* | 90 | 9.94 | 10.98 | 11.1 | **10.67** |
| *ZFAND5* | 90 | 39.83 | 43.52 | 41.38 | **41.58** |
| *ADGRL2* | 90 | 9.17 | 9.76 | 9.39 | **9.44** |
| *GNAL* | 90 | 0.48 | 0.59 | 0.57 | **0.55** |
| *CXXC4* | 90 | 0 | 0.03 | 0 | **0.01** |
| ***MIR885*** | | | | | |
| **miR-885-5p** | | | | | |
| *GALNT3* | 100 | 0.51 | 0.76 | 0.75 | **0.67** |
| *ZNF367* | 99 | 12.98 | 15.63 | 14.44 | **14.35** |
| *C9orf3* | 97 | 20.71 | 21.26 | 21.65 | **21.21** |
| *TMEM135* | 97 | 6.38 | 7.25 | 6.77 | **6.8** |
| *CTNNB1* | 97 | 212.42 | 215.61 | 210.5 | **212.84** |
| *ZNF354A* | 96 | 9.18 | 10.99 | 9.98 | **10.05** |
| *OXR1* | 96 | 24.2 | 30.28 | 28.83 | **27.77** |
| *SYTL4* | 95 | 1.98 | 2.51 | 1.99 | **2.16** |
| *CPEB2* | 95 | 6.23 | 7.09 | 6.44 | **6.59** |
| *ZFP91* | 95 | 33.28 | 37.77 | 34.63 | **35.23** |
| *LRP4* | 95 | 0.29 | 0.32 | 0.26 | **0.29** |
| *PGK2* | 94 | 0 | 0 | 0 | **0** |
| *SLC11A2* | 94 | 28.88 | 30.33 | 29.52 | **29.58** |
| *ZNF281* | 94 | 14.17 | 16.93 | 16.54 | **15.88** |
| *LCP1* | 93 | 1.12 | 1.09 | 1.19 | **1.13** |
| *CAMK4* | 93 | 0.63 | 0.67 | 0.71 | **0.67** |
| *FAM81A* | 93 | 0.65 | 0.72 | 0.7 | **0.69** |
| *MXD1* | 93 | 3.06 | 3.33 | 3.06 | **3.15** |
| *ASCL4* | 93 | 0 | 0 | 0 | **0** |
| *KIF21A* | 93 | 11.08 | 14.1 | 13.53 | **12.9** |
| *FAM241A* | 92 | 2.88 | 3.12 | 2.45 | **2.82** |
| *GPR68* | 92 | 0,00 | 0,00 | 0.02 | **0.01** |
| *CCDC71L* | 92 | 6.36 | 6.95 | 6.32 | **6.54** |
| *UPF2* | 92 | 16.69 | 20.73 | 18.84 | **18.75** |
| *ATXN1L* | 92 | 8.07 | 7.97 | 8.02 | **8.02** |
| *ADD1* | 92 | 38.03 | 40.08 | 40.58 | **39.56** |
| *C8orf88* | 92 | nd | nd | nd | **nd** |
| *JAZF1* | 91 | 4.96 | 5.67 | 5.58 | **5.4** |
| *MAN1C1* | 91 | 0.57 | 0.71 | 0.74 | **0.67** |
| *PANK3* | 91 | 66.58 | 79.6 | 75.03 | **73.74** |
| *ZADH2* | 91 | 7.53 | 9.08 | 7.98 | **8.2** |
| *PFDN4* | 91 | 35.46 | 41.66 | 36.97 | **38.03** |
| *ELAVL1* | 91 | 11.58 | 11.91 | 11.66 | **11.72** |
| *CDC73* | 90 | 13.79 | 17.39 | 16.13 | **15.77** |
| *OR51E1* | 90 | 0 | 0 | 0 | **0** |
| *FURIN* | 90 | 8.03 | 7.98 | 8.45 | **8.15** |
| *FNTA* | 90 | 55.84 | 62.4 | 58.63 | **58.96** |
| *ECE1* | 90 | 7.16 | 8.66 | 9.16 | **8.33** |
| **miR-885-3p** | | | | | |
| *RGS8* | 95 | 0 | 0 | 0 | **0** |
| *GPCPD1* | 94 | 9.3 | 10.38 | 9.43 | **9.7** |
| *RAVER2* | 93 | 4.75 | 5.53 | 5.37 | **5.22** |
| *SOCS6* | 92 | 17.37 | 19.69 | 18.24 | **18.43** |
| *SPATS2L* | 91 | 20.54 | 21.16 | 19.83 | **20.51** |
| *RARG* | 90 | 3.96 | 3.97 | 4.14 | **4.02** |
| *ARMH3* | 90 | 7.06 | 6.59 | 6.56 | **6.74** |
| *NAT8L* | 90 | 1.64 | 1.73 | 1.68 | **1.68** |
| ***MIR548I3*** | | | | | |
| **miR-548i** | | | | | |
| *FAM135A* | 100 | 8.72 | 10.85 | 10.45 | **10.01** |
| *NFAT5* | 100 | 6.01 | 7.06 | 7.13 | **6.73** |
| *RORA* | 100 | 3.41 | 4.57 | 4 | **3.99** |
| *FIGN* | 99 | 0.53 | 0.81 | 0.7 | **0.68** |
| *ACAT2* | 99 | 78.75 | 82.26 | 77.38 | **79.46** |
| *ZDHHC21* | 99 | 8.2 | 10.85 | 10.45 | **9.83** |
| *STXBP5* | 99 | 14.18 | 17.5 | 16.26 | **15.98** |
| *IGF2BP3* | 99 | 6.95 | 8.24 | 7.91 | **7.70** |
| *NEGR1* | 99 | 16.94 | 21.86 | 19.53 | **19.44** |
| *CCNY* | 99 | 33.76 | 37.39 | 35.24 | **35.46** |
| *DENND1B* | 99 | 10.31 | 13.64 | 13.19 | **12.38** |
| *PREX2* | 99 | 0.01 | 0 | 0 | **0.00** |
| *MINDY2* | 99 | 8.18 | 10.69 | 9.4 | **9.42** |
| *MEX3D* | 99 | 9.4 | 10.33 | 9.92 | **9.88** |
| *SACS* | 99 | 46.1 | 59.67 | 55.56 | **53.78** |
| *EPHA3* | 99 | 0.49 | 0.61 | 0.64 | **0.58** |
| *ADAM22* | 99 | 2.1 | 2.24 | 1.78 | **2.04** |
| *TTC13* | 98 | 5.99 | 6.53 | 6.27 | **6.26** |
| *REV3L* | 98 | 23.89 | 31.54 | 28.27 | **27.90** |
| *CNTN1* | 98 | 0.84 | 1.03 | 0.97 | **0.95** |
| *ELMSAN1* | 98 | 5.57 | 6.21 | 5.59 | **5.79** |
| *FLRT3* | 98 | 8.23 | 8.65 | 7.62 | **8.17** |
| *RMND5A* | 98 | 34.76 | 38.27 | 34.98 | **36.00** |
| *PRKAA1* | 98 | 29.97 | 35.34 | 33.17 | **32.83** |
| *BTF3L4* | 98 | 29.85 | 32.41 | 29.94 | **30.73** |
| *MAST3* | 98 | 0.57 | 0.56 | 0.65 | **0.59** |
| *LRRTM3* | 98 | 0 | 0.01 | 0 | **0.00** |
| *CDK6* | 98 | 80.16 | 102.01 | 95.78 | **92.65** |
| *RNF138* | 98 | 12.8 | 15.3 | 14.45 | **14.18** |
| *SRP9* | 98 | 338.65 | 380.15 | 354.7 | **357.83** |
| *RALA* | 98 | 69.28 | 77.54 | 71.23 | **72.68** |
| *AIDA* | 98 | 25.56 | 30.71 | 29.37 | **28.55** |
| *BMT2* | 98 | 11.39 | 13.88 | 12.58 | **12.62** |
| *UBE2A* | 98 | 41.53 | 42.39 | 41.58 | **41.83** |
| *C5orf24* | 98 | 35.77 | 41.77 | 37.04 | **38.19** |
| *ZNF680* | 98 | 9.89 | 15.15 | 12.61 | **12.55** |
| *GULP1* | 98 | 10.45 | 13.03 | 10.97 | **11.48** |
| *DNAJB14* | 97 | 30.13 | 39.67 | 36.03 | **35.28** |
| *S1PR1* | 97 | 1.34 | 1.57 | 1.69 | **1.53** |
| *STYX* | 97 | 11.27 | 13.42 | 11.93 | **12.21** |
| *SCML2* | 97 | 0.88 | 0.83 | 1.02 | **0.91** |
| *IKZF2* | 97 | 1.76 | 2.2 | 2.01 | **1.99** |
| *TMEM135* | 97 | 6.38 | 7.25 | 6.77 | **6.80** |
| *B3GALT5* | 97 | 0.11 | 0.33 | 0.28 | **0.24** |
| *WDR26* | 97 | 14.58 | 15.89 | 16.12 | **15.53** |
| *ADAMTS1* | 97 | 16.84 | 19.95 | 19.81 | **18.87** |
| *ADGRB3* | 97 | 0.02 | 0.01 | 0.03 | **0.02** |
| *PRRC1* | 97 | 23.98 | 24.34 | 24.06 | **24.13** |
| *ZEB2* | 97 | 36.81 | 40.23 | 39.74 | **38.93** |
| *MTA1* | 97 | 13.39 | 12.57 | 13.23 | **13.06** |
| *RC3H1* | 97 | 12.32 | 12.76 | 12.8 | **12.63** |
| *ZNF608* | 97 | 10.72 | 12.01 | 11.61 | **11.45** |
| *C6orf120* | 97 | 22.72 | 25.58 | 23.86 | **24.05** |
| *DEFA6* | 96 | 0 | 0 | 0 | **0.00** |
| *SAMD8* | 96 | 13.08 | 14.07 | 13.56 | **13.57** |
| *ZBTB11* | 96 | 15.96 | 18.95 | 17.93 | **17.61** |
| *ACTN4* | 96 | 187.75 | 218.37 | 214.63 | **206.92** |
| *SCAMP1* | 96 | 31.26 | 35.42 | 32.3 | **32.99** |
| *GPATCH11* | 96 | 9.46 | 8.79 | 8.71 | **8.99** |
| *AFDN* | 96 | 93.51 | 105.04 | 101.08 | **99.88** |
| *GPR85* | 96 | 0 | 0 | 0 | **0.00** |
| *EDIL3* | 96 | 9.02 | 10.39 | 9.02 | **9.48** |
| *ZBTB44* | 96 | 17.78 | 20.14 | 19.66 | **19.19** |
| *PPP1R9A* | 96 | 20.94 | 24.34 | 22.27 | **22.52** |
| *SNX16* | 96 | 4.23 | 5.04 | 5.41 | **4.89** |
| *SLC30A5* | 96 | 30.85 | 33.49 | 33.37 | **32.57** |
| *CAPN2* | 96 | 245.13 | 261.12 | 261.26 | **255.84** |
| *FEM1C* | 96 | 10.78 | 12.54 | 11.43 | **11.58** |
| *NUP50* | 96 | 26.47 | 29.56 | 27.75 | **27.93** |
| *GOPC* | 96 | 38.4 | 44.05 | 41.45 | **41.30** |
| *CEP120* | 96 | 9.85 | 11.37 | 10.47 | **10.56** |
| *FBXL3* | 96 | 34.86 | 42.5 | 39.19 | **38.85** |
| *ANKRD22* | 95 | 0.01 | 0.04 | 0.02 | **0.02** |
| *MYCN* | 95 | 0 | 0.04 | 0 | **0.01** |
| *CCSER1* | 92 | 0.51 | 0.52 | 0.64 | **0.56** |
| *SOX5* | 95 | 0.87 | 0.87 | 1.03 | **0.92** |
| *FZD7* | 95 | 0.35 | 0.3 | 0.39 | **0.35** |
| *TCF12* | 95 | 40.47 | 46.23 | 43.44 | **43.38** |
| *NFKB1* | 95 | 12.51 | 13.1 | 13.41 | **13.01** |
| *PPARG* | 95 | 6.67 | 6.73 | 6.46 | **6.62** |
| *KLF10* | 95 | 27.25 | 30.37 | 29.36 | **28.99** |
| *ARL6IP6* | 95 | 12.15 | 13.91 | 14.11 | **13.39** |
| *CTNNA3* | 95 | 0.05 | 0.15 | 0.09 | **0.10** |
| *GPALPP1* | 95 | 34.34 | 39.12 | 34.65 | **36.04** |
| *SIX4* | 95 | 3.33 | 3.84 | 3.5 | **3.56** |
| *HECA* | 95 | 9.51 | 11.04 | 10.51 | **10.35** |
| *NRG4* | 95 | 0 | 0 | 0 | **0.00** |
| *TMED7* | 95 | 104.32 | 120.62 | 112.41 | **112.45** |
| *LATS1* | 95 | 7.9 | 9.08 | 8.4 | **8.46** |
| *TRIM2* | 95 | 18.06 | 19.84 | 18.75 | **18.88** |
| *MAML1* | 95 | 3.33 | 3.61 | 3.69 | **3.54** |
| *CCNB1* | 95 | 31.57 | 35.66 | 36.95 | **34.73** |
| *MAGT1* | 95 | 35.38 | 35.99 | 36.54 | **35.97** |
| *PTPRG* | 95 | 4.21 | 4.71 | 4.64 | **4.52** |
| *UNC80* | 95 | 3.66 | 4.62 | 5.03 | **4.44** |
| *PLEKHG1* | 95 | 0.16 | 0.27 | 0.28 | **0.24** |
| *SPATA6L* | 95 | 0.98 | 0.88 | 1.07 | **0.98** |
| *CACUL1* | 94 | 24.24 | 28.59 | 25.5 | **26.11** |
| *NDC1* | 94 | 20.35 | 22.25 | 21.56 | **21.39** |
| *CBX3* | 94 | 178.99 | 214.68 | 197.72 | **197.13** |
| *BBS10* | 94 | 17.78 | 19.99 | 18.71 | **18.83** |
| *MBNL2* | 94 | 130.42 | 156.94 | 144.15 | **143.84** |
| *TMTC1* | 94 | 24.36 | 25.8 | 25.14 | **25.10** |
| *SLU7* | 94 | 31.86 | 37.19 | 34.07 | **34.37** |
| *SRSF3* | 94 | 139.58 | 150.72 | 145.49 | **145.26** |
| *UGT8* | 94 | 6.23 | 6.65 | 6.34 | **6.41** |
| *PTBP3* | 94 | 16.6 | 18.81 | 18.55 | **17.99** |
| *PGRMC2* | 94 | 41.06 | 47.84 | 44.54 | **44.48** |
| *EEA1* | 94 | 22.15 | 30.52 | 26.58 | **26.42** |
| *MIGA1* | 94 | 14.03 | 16.41 | 15.6 | **15.35** |
| *URI1* | 94 | 6.83 | 7.37 | 7.36 | **7.19** |
| *THSD7A* | 94 | 0.26 | 0.45 | 0.41 | **0.37** |
| *GCC2* | 94 | 20.68 | 29.25 | 23.95 | **24.63** |
| *RNF149* | 94 | 20.09 | 22.44 | 21.03 | **21.19** |
| *C11orf87* | 94 | 0 | 0 | 0 | **0.00** |
| *TBCA* | 94 | 180.44 | 199.15 | 178.84 | **186.14** |
| *RAB8B* | 94 | 34.85 | 39.81 | 36.91 | **37.19** |
| *SETD2* | 94 | 17.52 | 21.37 | 20.87 | **19.92** |
| *MBIP* | 94 | 17.06 | 18.08 | 16.16 | **17.10** |
| *RGS7BP* | 94 | 33.09 | 44.01 | 37.46 | **38.19** |
| *FAM199X* | 94 | 22.84 | 27.08 | 24.06 | **24.66** |
| *NOTUM* | 94 | 0.02 | 0.02 | 0 | **0.01** |
| *METTL6* | 93 | 16.35 | 18.46 | 17.87 | **17.56** |
| *METTL8* | 93 | 5.52 | 5.74 | 5.55 | **5.60** |
| *TRUB1* | 93 | 33.96 | 37.86 | 34.71 | **35.51** |
| *ELL2* | 93 | 21.3 | 23.15 | 22.19 | **22.21** |
| *PRELID2* | 93 | 7.94 | 7.74 | 7.8 | **7.83** |
| *MFSD8* | 93 | 9.19 | 10.59 | 10.49 | **10.09** |
| *LMX1A* | 93 | 0 | 0 | 0 | **0.00** |
| *ME1* | 93 | 12.17 | 13.35 | 12.16 | **12.56** |
| *SCN8A* | 93 | 3.8 | 3.93 | 3.38 | **3.70** |
| *ANKRD26* | 93 | 8.13 | 11.56 | 10.05 | **9.91** |
| *SLC24A3* | 93 | 0 | 0 | 0 | **0.00** |
| *MARCH6* | 93 | 28.81 | 31.2 | 30.53 | **30.18** |
| *EVI2A* | 93 | 0.03 | 0.33 | 0.13 | **0.16** |
| *MEIS2* | 93 | 22.73 | 26.29 | 24.61 | **24.54** |
| *KLF7* | 93 | 7.85 | 7.7 | 7.77 | **7.77** |
| *AGTR1* | 93 | 0 | 0.02 | 0 | **0.01** |
| *NUMB* | 93 | 12.57 | 12.06 | 13.17 | **12.60** |
| *GPD2* | 93 | 22.1 | 22.82 | 22.17 | **22.36** |
| *PRKAA2* | 93 | 10.13 | 12.12 | 11.09 | **11.11** |
| *RFC3* | 93 | 17.58 | 19.57 | 19.24 | **18.80** |
| *ZRANB2* | 93 | 33.88 | 45.57 | 42.79 | **40.75** |
| *GSE1* | 93 | 2.46 | 3.02 | 2.96 | **2.81** |
| *ITGAV* | 93 | 261.26 | 326.77 | 309.31 | **299.11** |
| *RHOQ* | 93 | 39.43 | 45.15 | 41.88 | **42.15** |
| *ODAPH* | 93 | 0.5 | 0.98 | 0.71 | **0.73** |
| *SLCO5A1* | 93 | 0.05 | 0.06 | 0.08 | **0.06** |
| *GTF3C3* | 93 | 26.63 | 29.72 | 26.77 | **27.71** |
| *NCKAP1* | 92 | 95.52 | 111.54 | 104.61 | **103.89** |
| *SLC4A7* | 92 | 37.96 | 45.53 | 43.17 | **42.22** |
| *MBNL3* | 92 | 1.84 | 2.1 | 1.89 | **1.94** |
| *BOD1L1* | 92 | 16.14 | 21.78 | 19.51 | **19.14** |
| *NTF3* | 92 | 0 | 0 | 0 | **0.00** |
| *C3orf38* | 92 | 20.89 | 22.56 | 21.07 | **21.51** |
| *ARL13B* | 92 | 7.03 | 8.44 | 8.32 | **7.93** |
| *TMEM65* | 92 | 13.56 | 15.16 | 13.78 | **14.17** |
| *FMNL2* | 92 | 39.14 | 46.23 | 42.88 | **42.75** |
| *LRRC7* | 92 | 2.28 | 2.48 | 2.16 | **2.31** |
| *CACNA2D3* | 92 | 0.52 | 0.61 | 0.8 | **0.64** |
| *C18orf25* | 92 | 14.26 | 16.1 | 15.08 | **15.15** |
| *ZNF326* | 92 | 32.48 | 41.81 | 35.25 | **36.51** |
| *ZNF792* | 92 | 2.18 | 2 | 2.15 | **2.11** |
| *CLCN4* | 92 | 4.61 | 5.08 | 5.06 | **4.92** |
| *PITX2* | 91 | 0.01 | 0 | 0 | **0.00** |
| *KIAA1841* | 91 | 5.44 | 7.14 | 5.98 | **6.19** |
| *CAMLG* | 91 | 35.52 | 40.34 | 38.15 | **38.00** |
| *SH3D19* | 91 | 18.29 | 19.52 | 18.66 | **18.82** |
| *ETF1* | 91 | 86.24 | 92.93 | 87.77 | **88.98** |
| *SKIDA1* | 91 | 0.34 | 0.28 | 0.28 | **0.30** |
| *MGARP* | 91 | 0.78 | 0.92 | 0.88 | **0.86** |
| *TMTC3* | 91 | 19.37 | 24.6 | 24.64 | **22.87** |
| *SCN3A* | 91 | 0.04 | 0.03 | 0.03 | **0.03** |
| *DNAJB4* | 91 | 67.86 | 83.15 | 73.3 | **74.77** |
| *EPB41L5* | 91 | 12.14 | 13.64 | 12.85 | **12.88** |
| *LCOR* | 91 | 5.82 | 7.36 | 6.72 | **6.63** |
| *ZNF148* | 91 | 20.79 | 24.46 | 22.81 | **22.69** |
| *SECISBP2L* | 91 | 21.3 | 25.38 | 22.47 | **23.05** |
| *CLVS2* | 91 | 0 | 0 | 0 | **0.00** |
| *MIER3* | 91 | 14.46 | 16.41 | 15.15 | **15.34** |
| *CCDC117* | 90 | 15.13 | 16.44 | 16.27 | **15.95** |
| *CBWD6* | 90 | 4.3 | 6.07 | 4.69 | **5.02** |
| *COMMD3-BMI1* | 90 | 24.25 | 25.22 | 24.1 | **24.52** |
| *ITGB6* | 90 | 0.07 | 0.18 | 0.12 | **0.12** |
| *ZNF492* | 90 | 0.76 | 1.01 | 0.93 | **0.90** |
| *CSGALNACT2* | 90 | 10.62 | 11.35 | 12.05 | **11.34** |
| *CBWD1* | 90 | 32.6 | 37.9 | 39.58 | **36.69** |
| *PTPRR* | 90 | 3.81 | 4.14 | 3.85 | **3.93** |
| *RAP2A* | 90 | 33.37 | 38.59 | 36.14 | **36.03** |
| *CBWD5* | 90 | 32.58 | 34.7 | 33.77 | **33.68** |
| *APPBP2* | 90 | 22.63 | 25.55 | 23.88 | **24.02** |
| *CBWD2* | 90 | 22.38 | 24.84 | 23.22 | **23.48** |
| *LACTB* | 90 | 42.59 | 50.43 | 47.08 | **46.70** |
| *SEC24A* | 90 | 9.73 | 11.14 | 10.82 | **10.56** |
| *SMAD5* | 90 | 37.82 | 43.68 | 41.64 | **41.05** |
| *MMUT* | 90 | 22.2 | 23.4 | 22.06 | **22.55** |
| *DHRS1* | 90 | 10.26 | 10.94 | 12.35 | **11.18** |
| *C21orf91* | 90 | 7.09 | 8.11 | 7.7 | **7.63** |
| *ARID2* | 90 | 15.27 | 15.79 | 15.32 | **15.46** |
| *ANKRD10* | 90 | 28.56 | 33.04 | 32.04 | **31.21** |
| *WDR47* | 90 | 17.65 | 17.54 | 16.79 | **17.33** |
| *CBWD3* | 90 | 22.94 | 25.77 | 26.43 | **25.05** |
| *NUP54* | 90 | 24.68 | 26.48 | 26.13 | **25.76** |
| *PCDH11X* | 90 | 0.25 | 0.28 | 0.28 | **0.27** |
| *MCF2L2* | 90 | 0.34 | 0.36 | 0.35 | **0.35** |
| *TRAM1* | 90 | 232.15 | 258.19 | 239.37 | **243.24** |
| *PAQR9* | 90 | 0.63 | 0.78 | 0.32 | **0.58** |
| *NOTCH2* | 90 | 45.63 | 50.15 | 51.32 | **49.03** |
| *MMP16* | 90 | 4.26 | 5.05 | 4.6 | **4.64** |
| *GABPA* | 90 | 20.58 | 24.28 | 23.17 | **22.68** |
| *BEND7* | 90 | 11.36 | 12.98 | 12.03 | **12.12** |
| *PSMC2* | 90 | 58.76 | 60.32 | 61.78 | **60.29** |
| *UGDH* | 90 | 28.48 | 31.55 | 29.24 | **29.76** |
| ***MIR6854*** | | | | | |
| **miR-6854-5p** | | | | | |
| *LIMCH1* | 97 | 178.52 | 205.84 | 192.73 | **192.36** |
| *CD36* | 95 | 0.53 | 0.38 | 0.35 | **0.42** |
| *ARMC8* | 94 | 22.01 | 23.57 | 22.09 | **22.56** |
| *NFE2L2* | 94 | 61.98 | 66.35 | 64.85 | **64.39** |
| *STON2* | 93 | 6.47 | 8.08 | 7.35 | **7.30** |
| *SMARCC1* | 93 | 35.12 | 40.71 | 36.8 | **37.54** |
| *METAP2* | 93 | 45.98 | 51.17 | 47.61 | **48.25** |
| *ADRA2B* | 92 | 0.02 | 0 | 0.01 | **0.01** |
| *CPEB1* | 92 | 0.28 | 0.39 | 0.3 | **0.32** |
| *JAG1* | 92 | 7.92 | 9.16 | 9.01 | **8.70** |
| *CLMN* | 92 | 0.73 | 0.7 | 0.73 | **0.72** |
| *FBN2* | 92 | 47.92 | 54.05 | 56.2 | **52.72** |
| *CNOT2* | 91 | 33.66 | 35.95 | 33.84 | **34.48** |
| *CAV1* | 91 | 21.64 | 23.34 | 21.69 | **22.22** |
| *PCSK7* | 91 | 5.97 | 5.84 | 6.8 | **6.20** |
| *DUSP22* | 90 | 8.05 | 8.39 | 7.78 | **8.07** |
| *STARD7* | 90 | 76.46 | 80.63 | 81.54 | **79.54** |
| *RABGGTB* | 90 | 114.88 | 123.63 | 114.3 | **117.60** |
| *KLRG1* | 90 | 0.34 | 0.24 | 0.32 | **0.30** |
| **miR-6854-3p** | | | | | |
| *CHSY3* | 91 | 0.5 | 0.51 | 0.52 | **0.51** |
| *UNC5B* | 90 | 0.75 | 0.8 | 0.84 | **0.80** |
| ***MIR675*** | | | | | |
| **miR-675-5p** | | | | | |
| *RUNX1* | 93 | 4.9 | 6.5 | 6.01 | **5.80** |
| **miR-675-3p** | | | | | |
| *ATXN1* | 96 | 4.76 | 5.81 | 4.78 | **5.12** |
| *ADAM22* | 95 | 2.1 | 2.24 | 1.78 | **2.04** |
| *AGPS* | 95 | 37.41 | 42.65 | 40.08 | **40.05** |
| *SERPINF1* | 94 | 8.97 | 10.16 | 10.85 | **9.99** |
| *CHN2* | 94 | 0.17 | 0.26 | 0.18 | **0.20** |
| *CUL3* | 93 | 19.15 | 20.64 | 20.17 | **19.99** |
| *CSMD3* | 93 | 0.36 | 0.43 | 0.34 | **0.38** |
| *PLPP4* | 92 | 25.08 | 28.32 | 27.37 | **26.92** |
| *FMR1* | 91 | 24.54 | 29.7 | 28.29 | **27.51** |
| *PPP4R3A* | 91 | 36.59 | 46.19 | 41.34 | **41.37** |
| *CDH11* | 90 | 80.35 | 95.53 | 92.74 | **89.54** |
| *PAPOLA* | 90 | 47.54 | 56.38 | 53.52 | **52.48** |
| *KMT5A* | 90 | 16.27 | 17.25 | 17.31 | **16.94** |
| *FAM227B* | 90 | 1.56 | 2.01 | 1.5 | **1.69** |
| *DDX3X* | 90 | 113.09 | 121.45 | 117.32 | **117.29** |
| *KCNA4* | 90 | 0.05 | 0.11 | 0.05 | **0.07** |
| ***MIRLET7C*** | | | | | |
| **let-7c-5p** | | | | | |
| *FIGNL2* | 100 | 0 | 0 | 0.01 | **0.00** |
| *HMGA2* | 100 | 3.8 | 5.34 | 5.03 | **4.72** |
| *IGF2BP1* | 100 | 0.02 | 0.02 | 0.03 | **0.02** |
| *LIN28B* | 100 | 0 | 0 | 0 | **0.00** |
| *TRIM71* | 100 | 0.06 | 0.03 | 0.01 | **0.03** |
| *NR6A1* | 100 | 0.17 | 0.09 | 0.09 | **0.12** |
| *STARD13* | 100 | 17.48 | 19.73 | 19.34 | **18.85** |
| *IGDCC3* | 100 | 0.06 | 0.1 | 0.1 | **0.09** |
| *PRTG* | 100 | 4.8 | 5.06 | 5.17 | **5.01** |
| *C14orf28* | 100 | 4.67 | 4.79 | 4.32 | **4.59** |
| *ARID3B* | 100 | 0.73 | 0.95 | 0.79 | **0.82** |
| *FRMD4B* | 99 | 10.38 | 12.12 | 11.01 | **11.17** |
| *SMARCAD1* | 99 | 33.38 | 42.41 | 40.41 | **38.73** |
| *NPHP3* | 99 | 12.05 | 13.32 | 12.74 | **12.70** |
| *PTAFR* | 99 | 0.3 | 0.35 | 0.43 | **0.36** |
| *GATM* | 99 | 3.18 | 3.23 | 3.42 | **3.28** |
| *FIGN* | 99 | 0.53 | 0.81 | 0.7 | **0.68** |
| *HIC2* | 98 | 0.45 | 0.41 | 0.53 | **0.46** |
| *CBX5* | 98 | 36.65 | 41.16 | 37.76 | **38.52** |
| *PGRMC1* | 98 | 85 | 96.45 | 89.11 | **90.19** |
| *NAP1L1* | 98 | 707.49 | 781.91 | 710.25 | **733.22** |
| *CCND2* | 98 | 2.02 | 2.11 | 1.97 | **2.03** |
| *ZNF512B* | 97 | 4.78 | 5.04 | 5.09 | **4.97** |
| *MIB1* | 97 | 26.8 | 31.65 | 29.21 | **29.22** |
| *SLC10A7* | 97 | 2.5 | 3.07 | 3.06 | **2.88** |
| *CDC34* | 97 | 17.01 | 19.1 | 18.81 | **18.31** |
| *PAPPA* | 97 | 0.05 | 0.04 | 0.04 | **0.04** |
| *SALL4* | 97 | 0.01 | 0.05 | 0.02 | **0.03** |
| *LRIG3* | 97 | 6.7 | 7.41 | 7.01 | **7.04** |
| *NME6* | 97 | 9.33 | 10.79 | 10.21 | **10.11** |
| *SFMBT1* | 97 | 3.24 | 3.87 | 4.06 | **3.72** |
| *SLF2* | 97 | 16.41 | 18.42 | 17.2 | **17.34** |
| *ADAMTS15* | 97 | 0.96 | 1.11 | 1.31 | **1.13** |
| *ZBTB5* | 96 | 7.41 | 7.52 | 6.94 | **7.29** |
| *ZNF710* | 96 | 0.85 | 0.9 | 0.97 | **0.91** |
| *UTRN* | 96 | 40.93 | 50.24 | 48.5 | **46.56** |
| *STX3* | 96 | 54.48 | 58.46 | 57.54 | **56.83** |
| *SKIL* | 96 | 33.18 | 42.18 | 39.22 | **38.19** |
| *PIK3IP1* | 96 | 24.23 | 23.37 | 23.25 | **23.62** |
| *PPP1R15B* | 96 | 23.85 | 27.26 | 25.95 | **25.69** |
| *GXYLT1* | 96 | 7.03 | 8.34 | 8.11 | **7.83** |
| *GNG5* | 96 | 51.77 | 59.47 | 55.57 | **55.60** |
| *LPGAT1* | 96 | 10.97 | 13.1 | 12.91 | **12.33** |
| *YOD1* | 96 | 25.37 | 29.62 | 27.57 | **27.52** |
| *ZSWIM5* | 96 | 0.04 | 0.06 | 0.04 | **0.05** |
| *IGF1R* | 96 | 8.53 | 10.98 | 11.43 | **10.31** |
| *USP44* | 96 | 5.91 | 6.88 | 6.08 | **6.29** |
| *FZD3* | 96 | 2.23 | 2.49 | 2.43 | **2.38** |
| *CCNJ* | 96 | 8.48 | 9.72 | 8.36 | **8.85** |
| *DLC1* | 96 | 19.65 | 23.17 | 22.96 | **21.93** |
| *NRAS* | 96 | 43.34 | 47.83 | 44.34 | **45.17** |
| *BACH1* | 96 | 31.23 | 36.65 | 33.95 | **33.94** |
| *COIL* | 95 | 15.35 | 15.81 | 15.33 | **15.50** |
| *SMC1A* | 95 | 12.7 | 14.26 | 13.82 | **13.59** |
| *C8orf58* | 95 | 6.63 | 6.56 | 7.06 | **6.75** |
| *PXT1* | 95 | 0 | 0 | 0.05 | **0.02** |
| *CLCN5* | 95 | 1.49 | 1.62 | 1.69 | **1.60** |
| *THRSP* | 95 | 0 | 0 | 0.02 | **0.01** |
| *COL3A1* | 95 | 0.03 | 0.02 | 0 | **0.02** |
| *TET3* | 95 | 1.32 | 1.42 | 1.39 | **1.38** |
| *SMIM3* | 95 | 4.97 | 4.74 | 4.59 | **4.77** |
| *AGO4* | 95 | 8.88 | 9.13 | 9.05 | **9.02** |
| *CLDN12* | 95 | 27.08 | 30.78 | 28.18 | **28.68** |
| *BIN3* | 95 | 8.55 | 8.65 | 8.39 | **8.53** |
| *NIPAL4* | 95 | 4.57 | 5.49 | 5.16 | **5.07** |
| *ZNF644* | 95 | 34.51 | 45.54 | 41.97 | **40.67** |
| *GNPTAB* | 95 | 73.65 | 80.8 | 74.76 | **76.40** |
| *ADAMTS8* | 95 | 0 | 0.01 | 0.02 | **0.01** |
| *ADRB2* | 95 | 12.68 | 12.41 | 11.42 | **12.17** |
| *E2F5* | 94 | 12.06 | 13.34 | 12.08 | **12.49** |
| *ATP8B4* | 94 | 2.44 | 2.86 | 2.71 | **2.67** |
| *ADRB3* | 94 | 0 | 0 | 0 | **0.00** |
| *CPA4* | 94 | 45.09 | 48.63 | 49.22 | **47.65** |
| *IGF2BP3* | 94 | 6.95 | 8.24 | 7.91 | **7.70** |
| *STK40* | 94 | 5.41 | 5.95 | 5.39 | **5.58** |
| *DLST* | 94 | 37.2 | 36.42 | 36.1 | **36.57** |
| *MAP4K3* | 94 | 26.17 | 30.09 | 26.61 | **27.62** |
| *SLC16A9* | 94 | 0.02 | 0.1 | 0.1 | **0.07** |
| *C5orf51* | 94 | 8.96 | 10.26 | 9.58 | **9.60** |
| *TGFBR3* | 94 | 16.14 | 17.55 | 17.27 | **16.99** |
| *SLC35D2* | 94 | 21.02 | 23.06 | 21.91 | **22.00** |
| *HAND1* | 94 | 0 | 0 | 0 | **0.00** |
| *ONECUT2* | 94 | 0.16 | 0.18 | 0.19 | **0.18** |
| *DTX4* | 93 | 2.1 | 2.12 | 2.49 | **2.24** |
| *SENP2* | 93 | 16.85 | 17.63 | 17.04 | **17.17** |
| *EDN1* | 93 | 6.02 | 7.14 | 7.42 | **6.86** |
| *PLEKHA8* | 93 | 7.89 | 9.56 | 8.72 | **8.72** |
| *TMPRSS2* | 93 | 0 | 0 | 0 | **0.00** |
| *FRAS1* | 93 | 2.31 | 3.19 | 3.25 | **2.92** |
| *PTPRD* | 93 | 4.12 | 4.52 | 4.47 | **4.37** |
| *MARS2* | 93 | 4.38 | 4.9 | 4.95 | **4.74** |
| *FAM189A1* | 93 | 1.71 | 1.72 | 1.82 | **1.75** |
| *ARHGEF38* | 93 | 0.01 | 0.01 | 0 | **0.01** |
| *PEX11B* | 93 | 14.11 | 15.59 | 15.56 | **15.09** |
| *DNA2* | 93 | 4.99 | 6.13 | 5.62 | **5.58** |
| *AHCTF1* | 93 | 22.83 | 27.17 | 26.77 | **25.59** |
| *DMD* | 93 | 32.34 | 40.18 | 36.41 | **36.31** |
| *ZNF280B* | 93 | 1.91 | 1.72 | 1.81 | **1.81** |
| *GAS7* | 93 | 9.92 | 10.1 | 10.51 | **10.18** |
| *DTX2* | 93 | 1.13 | 1.11 | 1.28 | **1.17** |
| *BZW1* | 92 | 201.92 | 226.46 | 208.43 | **212.27** |
| *CEMIP2* | 92 | 15.57 | 17.78 | 17.36 | **16.90** |
| *TSEN34* | 92 | 19.6 | 19.61 | 19.44 | **19.55** |
| *USP38* | 92 | 17.72 | 19.11 | 18.91 | **18.58** |
| *ITGB3* | 92 | 5.55 | 6.08 | 6.21 | **5.95** |
| *WNT9B* | 92 | 0.29 | 0.43 | 0.31 | **0.34** |
| *CPEB2* | 92 | 6.23 | 7.09 | 6.44 | **6.59** |
| *ZBP1* | 92 | 0.07 | 0.05 | 0.08 | **0.07** |
| *PCGF3* | 92 | 16.42 | 16.6 | 16.14 | **16.39** |
| *COL1A2* | 92 | 0 | 0 | 0 | **0.00** |
| *HAS2* | 92 | 0 | 0 | 0 | **0.00** |
| *LAMP2* | 92 | 115.84 | 128.44 | 121.56 | **121.95** |
| *CLP1* | 92 | 11.11 | 12.06 | 10.73 | **11.30** |
| *CPEB1* | 92 | 0.28 | 0.39 | 0.3 | **0.32** |
| *PBX2* | 92 | 8.54 | 9.05 | 8.99 | **8.86** |
| *TGFBR1* | 92 | 31.16 | 33.64 | 30.87 | **31.89** |
| *TTLL4* | 92 | 3.18 | 3.65 | 3.73 | **3.52** |
| *PXDN* | 92 | 34.09 | 40.25 | 43.31 | **39.22** |
| *ZNF322* | 92 | 23.66 | 25.2 | 24.71 | **24.52** |
| *NHLRC3* | 92 | 11.5 | 12.65 | 11.89 | **12.01** |
| *RASGRP1* | 92 | 5.71 | 7.04 | 6.95 | **6.57** |
| *TMEM65* | 92 | 13.56 | 15.16 | 13.78 | **14.17** |
| *RUFY3* | 92 | 36.37 | 45.17 | 41.88 | **41.14** |
| *MDM4* | 92 | 11.24 | 13 | 11.81 | **12.02** |
| *COL4A2* | 92 | 34.37 | 40.47 | 43 | **39.28** |
| *TRIM67* | 92 | 0 | 0 | 0 | **0.00** |
| *GDF6* | 92 | 0.25 | 0.24 | 0.25 | **0.25** |
| *AMT* | 91 | 0.68 | 0.84 | 0.72 | **0.75** |
| *SLC5A9* | 91 | 0 | 0 | 0 | **0.00** |
| *MAPK6* | 91 | 58.95 | 64.85 | 59.31 | **61.04** |
| *ZFYVE26* | 91 | 7.95 | 8.61 | 8.57 | **8.38** |
| *IGDCC4* | 91 | 0.13 | 0.12 | 0.17 | **0.14** |
| *E2F6* | 91 | 9.29 | 9.85 | 9.65 | **9.60** |
| *FAM135A* | 91 | 8.72 | 10.85 | 10.45 | **10.01** |
| *B3GNT7* | 91 | 0.05 | 0.05 | 0.03 | **0.04** |
| *PBX1* | 91 | 3.61 | 4.35 | 3.85 | **3.94** |
| *FNIP1* | 91 | 22.67 | 25.92 | 23.55 | **24.05** |
| *ASAP1* | 91 | 8.94 | 9.76 | 9.28 | **9.33** |
| *ERCC6* | 91 | 16.63 | 18.66 | 17.43 | **17.57** |
| *ACVR1C* | 91 | 0.06 | 0.05 | 0.03 | **0.05** |
| *RGS16* | 91 | 0.14 | 0.14 | 0.16 | **0.15** |
| *DDI2* | 91 | 5.59 | 6.67 | 5.72 | **5.99** |
| *XRN1* | 91 | 22.59 | 26.94 | 25.22 | **24.92** |
| *PLXNC1* | 91 | 1.87 | 2.02 | 1.77 | **1.89** |
| *HECTD2* | 91 | 5.32 | 7.09 | 5.99 | **6.13** |
| *TMPPE* | 91 | 1.98 | 2.42 | 2.17 | **2.19** |
| *SOCS4* | 91 | 19.46 | 23.63 | 21.61 | **21.57** |
| *PBX3* | 91 | 54.16 | 60.25 | 55.4 | **56.60** |
| *CEP135* | 91 | 8.04 | 10.26 | 8.9 | **9.07** |
| *RBFOX2* | 91 | 26.64 | 29.35 | 27.12 | **27.70** |
| *PALD1* | 90 | 1.47 | 1.66 | 1.62 | **1.58** |
| *GALNT2* | 90 | 44.47 | 47.65 | 46.89 | **46.34** |
| *CARNMT1* | 90 | 13.17 | 14.44 | 14.69 | **14.10** |
| *IL13* | 90 | 0 | 0 | 0 | **0.00** |
| *PLXND1* | 90 | 2.72 | 2.7 | 2.74 | **2.72** |
| *SRGAP1* | 90 | 1.13 | 1.6 | 1.69 | **1.47** |
| *HIP1* | 90 | 4.39 | 5.21 | 5.06 | **4.89** |
| *THOC2* | 90 | 36.1 | 53.48 | 45.47 | **45.02** |
| *FAM122A* | 90 | 7.53 | 8.4 | 8.68 | **8.20** |
| *KCTD17* | 90 | 4.65 | 4.2 | 4.46 | **4.44** |
| *LIMD2* | 90 | 0.72 | 0.94 | 0.68 | **0.78** |
| *KLHDC8B* | 90 | 11.06 | 10.36 | 10.93 | **10.78** |
| *CD59* | 90 | 117.65 | 123.3 | 121.85 | **120.93** |
| *DPP6* | 90 | 0 | 0 | 0.01 | **0.00** |
| *DDX19B* | 90 | 14.55 | 16.46 | 15.44 | **15.48** |
| *GALC* | 90 | 20.75 | 22.15 | 21.11 | **21.34** |
| *KCTD21* | 90 | 3.45 | 2.77 | 3.32 | **3.18** |
| *BEGAIN* | 90 | 1.32 | 1.2 | 1.35 | **1.29** |
| *CNOT6L* | 90 | 5.68 | 6.19 | 5.96 | **5.94** |
| *HSPA14* | 90 | 24.54 | 28.8 | 27.25 | **26.86** |
| **let-7c-3p** | | | | | |
| *ARID4B* | 100 | 31.46 | 41.48 | 36.17 | **36.37** |
| *PLPPR1* | 100 | 0.03 | 0.03 | 0 | **0.02** |
| *FOXO1* | 100 | 2.47 | 2.83 | 2.43 | **2.58** |
| *SOX2* | 100 | 0 | 0 | 0.01 | **0.00** |
| *KIAA2026* | 100 | 6.39 | 8.8 | 7.51 | **7.57** |
| *FBXL3* | 100 | 34.86 | 42.5 | 39.19 | **38.85** |
| *GPM6A* | 100 | 8.57 | 9.19 | 9.12 | **8.96** |
| *MIER3* | 100 | 14.46 | 16.41 | 15.15 | **15.34** |
| *PPP2R5C* | 99 | 49.67 | 58.93 | 55.19 | **54.60** |
| *NAMPT* | 99 | 37.94 | 44.65 | 40.7 | **41.10** |
| *SIRT1* | 99 | 11.14 | 11.69 | 11.07 | **11.30** |
| *EPHA5* | 99 | 2.6 | 3.08 | 3.22 | **2.97** |
| *PRKCE* | 99 | 1.1 | 1.22 | 1.22 | **1.18** |
| *PUM1* | 99 | 42.08 | 45.51 | 43.52 | **43.70** |
| *OTUD1* | 99 | 11.29 | 11.51 | 11.49 | **11.43** |
| *PHIP* | 99 | 18.54 | 22.67 | 20.74 | **20.65** |
| *MAP4K3* | 99 | 26.17 | 30.09 | 26.61 | **27.62** |
| *ADAM10* | 99 | 52.36 | 62.32 | 58.19 | **57.62** |
| *PDIA3* | 99 | 207.95 | 214.42 | 202.81 | **208.39** |
| *TLK1* | 99 | 11.67 | 13.83 | 12.89 | **12.80** |
| *KRAS* | 99 | 14.76 | 16.59 | 15.31 | **15.55** |
| *ZFAND5* | 99 | 39.83 | 43.52 | 41.38 | **41.58** |
| *ANKRD12* | 99 | 16.9 | 20.65 | 18.17 | **18.57** |
| *RAB40C* | 99 | 2.32 | 2.83 | 2.62 | **2.59** |
| *RAP1B* | 99 | 98.53 | 108.27 | 100.02 | **102.27** |
| *CHL1* | 99 | 0 | 0 | 0 | **0.00** |
| *TCERG1L* | 99 | 0 | 0.01 | 0 | **0.00** |
| *KDM2B* | 99 | 5.79 | 7.46 | 7.11 | **6.79** |
| *TAOK1* | 99 | 32.12 | 38.27 | 36.32 | **35.57** |
| *SLC25A12* | 99 | 6.23 | 7.13 | 6.25 | **6.54** |
| *SLAIN2* | 99 | 13.92 | 16.4 | 15.93 | **15.42** |
| *HERC1* | 99 | 10.6 | 10.86 | 10.66 | **10.71** |
| *PLK1* | 99 | 3.85 | 3.48 | 3.85 | **3.73** |
| *HECTD2* | 99 | 5.32 | 7.09 | 5.99 | **6.13** |
| *RARB* | 99 | 70.52 | 76.58 | 75.47 | **74.19** |
| *AKAP1* | 99 | 9 | 8.85 | 9.41 | **9.09** |
| *MEGF11* | 99 | 0.07 | 0.14 | 0.08 | **0.10** |
| *ELL* | 99 | 1.79 | 1.9 | 1.69 | **1.79** |
| *FAXC* | 99 | 1.6 | 1.8 | 1.96 | **1.79** |
| *MBD5* | 99 | 3.77 | 4.33 | 3.95 | **4.02** |
| *SETBP1* | 99 | 0.73 | 1.15 | 1.02 | **0.97** |
| *SORBS2* | 99 | 0.63 | 0.48 | 0.76 | **0.62** |
| *CDH11* | 99 | 80.35 | 95.53 | 92.74 | **89.54** |
| *HMGN1* | 99 | 239.34 | 272.72 | 256.25 | **256.10** |
| *RBBP5* | 98 | 13.93 | 14.63 | 14.07 | **14.21** |
| *NAP1L4* | 98 | 85.62 | 87.07 | 84.66 | **85.78** |
| *ADCY6* | 98 | 6.49 | 7.43 | 7.72 | **7.21** |
| *COMMD3-BMI1* | 98 | 24.25 | 25.22 | 24.1 | **24.52** |
| *MBTD1* | 98 | 9.41 | 10.61 | 10.42 | **10.15** |
| *CSMD1* | 98 | 0.15 | 0.15 | 0.19 | **0.16** |
| *RAPGEF2* | 98 | 21.97 | 24.46 | 23.53 | **23.32** |
| *SP3* | 98 | 34.19 | 38.21 | 37.08 | **36.49** |
| *MRTFB* | 98 | 3.42 | 3.71 | 3.36 | **3.50** |
| *GRHL2* | 98 | 0.18 | 0.35 | 0.21 | **0.25** |
| *CDC42SE2* | 98 | 19.15 | 20.75 | 19.19 | **19.70** |
| *SEC63* | 98 | 8.37 | 10.22 | 9.25 | **9.28** |
| *BBX* | 98 | 28.71 | 35.29 | 33.8 | **32.60** |
| *CITED2* | 98 | 69.31 | 80.06 | 73.76 | **74.38** |
| *JMJD1C* | 98 | 36.66 | 48.75 | 44.02 | **43.14** |
| *SUSD6* | 95 | 2.39 | 2.81 | 2.68 | **2.63** |
| *SEC11A* | 98 | 157.67 | 171.15 | 168.58 | **165.80** |
| *HSPD1* | 98 | 378.39 | 406.4 | 389.52 | **391.44** |
| *UBE2K* | 98 | 16.26 | 20.5 | 18.23 | **18.33** |
| *MEMO1* | 98 | 41.79 | 43.9 | 42.79 | **42.83** |
| *PCLO* | 98 | 2.56 | 3.24 | 3.06 | **2.95** |
| *ADAMTSL1* | 98 | 0.13 | 0.09 | 0.04 | **0.09** |
| *FGF7* | 98 | 2.11 | 2.67 | 2.57 | **2.45** |
| *TSC22D1* | 97 | 22.48 | 24.66 | 24.32 | **23.82** |
| *SERPINE2* | 97 | 2.46 | 3.51 | 3.43 | **3.13** |
| *CADM2* | 97 | 1.2 | 1.6 | 1.34 | **1.38** |
| *ATP5MD* | 97 | 272.28 | 299.53 | 283.64 | **285.15** |
| *RAB10* | 97 | 75.02 | 86.15 | 79.16 | **80.11** |
| *DSCAML1* | 97 | 0 | 0 | 0 | **0.00** |
| *CSMD2* | 97 | 0.04 | 0.04 | 0.07 | **0.05** |
| *DYRK4* | 97 | 21.85 | 21.41 | 23.2 | **22.15** |
| *LRP12* | 97 | 21.06 | 22.71 | 21.33 | **21.70** |
| *UBE2G1* | 97 | 19.22 | 21.84 | 20.23 | **20.43** |
| *HIVEP2* | 97 | 6.01 | 6.63 | 6.21 | **6.28** |
| *ORC4* | 97 | 25.42 | 27.55 | 26.51 | **26.49** |
| *DACH1* | 97 | 0.38 | 0.63 | 0.51 | **0.51** |
| *ARFGEF1* | 97 | 23.57 | 26.05 | 24.68 | **24.77** |
| *PDE10A* | 97 | 1.94 | 2.31 | 2.35 | **2.20** |
| *SLC24A2* | 97 | 0.02 | 0.02 | 0.01 | **0.02** |
| *NFKBIA* | 97 | 21.9 | 21.06 | 22.26 | **21.74** |
| *KLHL14* | 97 | 0 | 0 | 0 | **0.00** |
| *TRRAP* | 97 | 3.26 | 4.18 | 4.31 | **3.92** |
| *ZIC5* | 97 | 0 | 0 | 0 | **0.00** |
| *ATP13A3* | 96 | 327.17 | 376.22 | 355.23 | **352.87** |
| *NUDT21* | 96 | 53.62 | 59.01 | 55.38 | **56.00** |
| *CEP170* | 96 | 42.72 | 53.41 | 48.65 | **48.26** |
| *SUMO3* | 96 | 51.95 | 56.95 | 54.93 | **54.61** |
| *HNRNPU* | 96 | 105.15 | 123.25 | 115.15 | **114.52** |
| *GABPB1* | 96 | 14.12 | 15.83 | 15.5 | **15.15** |
| *GPHN* | 96 | 3.42 | 3.47 | 3.2 | **3.36** |
| *ZMIZ1* | 96 | 4.58 | 5.02 | 5.53 | **5.04** |
| *MYT1L* | 96 | 0 | 0.01 | 0 | **0.00** |
| *WWC1* | 96 | 22.52 | 24.18 | 26.24 | **24.31** |
| *EBF2* | 96 | 0.07 | 0.04 | 0.08 | **0.06** |
| *NKTR* | 96 | 25.34 | 34.17 | 30.38 | **29.96** |
| *NR4A3* | 96 | 0 | 0.01 | 0.01 | **0.01** |
| *FGF18* | 96 | 0.46 | 0.52 | 0.39 | **0.46** |
| *KPNA4* | 96 | 27.81 | 30.86 | 29.5 | **29.39** |
| *SUN1* | 96 | 39.22 | 43.51 | 41.57 | **41.43** |
| *EP300* | 96 | 3.5 | 4.42 | 4.33 | **4.08** |
| *PLS3* | 96 | 226.79 | 246.88 | 234.58 | **236.08** |
| *TCF7L2* | 96 | 2.29 | 2.95 | 2.6 | **2.61** |
| *LRIG1* | 96 | 1.84 | 2.16 | 2.58 | **2.19** |
| *DNAJC8* | 96 | 90.04 | 94.19 | 90.41 | **91.55** |
| *JAZF1* | 96 | 4.96 | 5.67 | 5.58 | **5.40** |
| *ATAD2B* | 96 | 4.59 | 5.43 | 5.3 | **5.11** |
| *ENKD1* | 96 | 5.55 | 5.31 | 6.67 | **5.84** |
| *GDNF* | 96 | 0.06 | 0.06 | 0.05 | **0.06** |
| *SNRPB2* | 96 | 58.41 | 63.22 | 61.71 | **61.11** |
| *PPP3R1* | 96 | 17.96 | 18.57 | 17.72 | **18.08** |
| *DNAJA2* | 96 | 56.01 | 58.41 | 57.36 | **57.26** |
| *PUS7* | 96 | 15.22 | 16.73 | 16.16 | **16.04** |
| *PARD6B* | 96 | 21.19 | 23.07 | 21.58 | **21.95** |
| *HAPLN1* | 96 | 0 | 0 | 0 | **0.00** |
| *GLIS3* | 96 | 9.3 | 9.35 | 11.13 | **9.93** |
| *PHF2* | 96 | 5.86 | 7.15 | 6.42 | **6.48** |
| *EIF4B* | 95 | 598.9 | 625.05 | 605.19 | **609.71** |
| *BAZ1A* | 95 | 19.64 | 24.97 | 22.87 | **22.49** |
| *MEF2C* | 95 | 0.21 | 0.36 | 0.3 | **0.29** |
| *HMGCR* | 95 | 57.6 | 62.79 | 59.7 | **60.03** |
| *SETD1A* | 95 | 0.7 | 0.95 | 1.03 | **0.89** |
| *BPTF* | 95 | 16.09 | 18.7 | 18.38 | **17.72** |
| *SLIT2* | 95 | 1.59 | 2.07 | 2.06 | **1.91** |
| *ERBIN* | 95 | 41.97 | 52.71 | 48.04 | **47.57** |
| *COL4A6* | 95 | 0.63 | 0.54 | 0.52 | **0.56** |
| *CLDN23* | 95 | 0.53 | 0.56 | 0.53 | **0.54** |
| *PPP1R12A* | 95 | 34.15 | 42.37 | 39.78 | **38.77** |
| *MED4* | 95 | 24.53 | 27.51 | 24.41 | **25.48** |
| *P3H2* | 95 | 10.13 | 11.93 | 11.91 | **11.32** |
| *UBR5* | 95 | 29.68 | 32.58 | 31.88 | **31.38** |
| *ZHX2* | 95 | 3.9 | 4.47 | 3.75 | **4.04** |
| *TRIO* | 95 | 57.18 | 62.79 | 65.01 | **61.66** |
| *COL13A1* | 95 | 0 | 0 | 0.01 | **0.00** |
| *TAB3* | 95 | 10.95 | 12.13 | 11.68 | **11.59** |
| *FBXO38* | 95 | 26.3 | 28.31 | 27.2 | **27.27** |
| *BRD4* | 95 | 2.03 | 2.58 | 2.59 | **2.40** |
| *RXRA* | 95 | 2.9 | 2.93 | 3.23 | **3.02** |
| *ZNF638* | 95 | 54.03 | 67.49 | 62.59 | **61.37** |
| *ZNF148* | 95 | 20.79 | 24.46 | 22.81 | **22.69** |
| *SUMO2* | 95 | 275.38 | 305.88 | 278.91 | **286.72** |
| *PPP6R3* | 95 | 53.75 | 59.72 | 57.89 | **57.12** |
| *RORA* | 95 | 3.41 | 4.57 | 4 | **3.99** |
| *SPRY2* | 95 | 24.44 | 25.9 | 24.15 | **24.83** |
| *GCLC* | 95 | 15.33 | 17.27 | 15.49 | **16.03** |
| *FZD1* | 94 | 15.24 | 16.8 | 16.36 | **16.13** |
| *PIM3* | 94 | 5.78 | 5.38 | 5.27 | **5.48** |
| *L3MBTL3* | 94 | 7.78 | 8.42 | 7.58 | **7.93** |
| *GDF6* | 94 | 0.25 | 0.24 | 0.25 | **0.25** |
| *TTLL5* | 94 | 10.06 | 10.79 | 10.68 | **10.51** |
| *CREBRF* | 94 | 17.69 | 20.51 | 20.89 | **19.70** |
| *BCL11A* | 94 | 0.14 | 0.03 | 0.03 | **0.07** |
| *SP1* | 94 | 13.81 | 15.92 | 15.32 | **15.02** |
| *IRF6* | 94 | 1.24 | 1.32 | 1.13 | **1.23** |
| *ATP1B3* | 94 | 60.54 | 69.7 | 62.28 | **64.17** |
| *TP63* | 94 | 1.13 | 1.51 | 1.28 | **1.31** |
| *AFF4* | 94 | 35.47 | 42.43 | 40.02 | **39.31** |
| *PDE4A* | 94 | 0.29 | 0.25 | 0.31 | **0.28** |
| *ANTXR1* | 94 | 46.81 | 52.21 | 48.81 | **49.28** |
| *TNFRSF11B* | 94 | 0.23 | 0.26 | 0.17 | **0.22** |
| *GLRB* | 94 | 41.84 | 48.3 | 46.25 | **45.46** |
| *FMN1* | 94 | 1.23 | 1.66 | 1.37 | **1.42** |
| *SEC24A* | 94 | 9.73 | 11.14 | 10.82 | **10.56** |
| *KLF9* | 94 | 12.66 | 14.59 | 14 | **13.75** |
| *ASCL1* | 94 | 0.09 | 0.06 | 0.03 | **0.06** |
| *JARID2* | 94 | 4.82 | 4.99 | 4.44 | **4.75** |
| *ZNF12* | 94 | 20.5 | 23.04 | 21.6 | **21.71** |
| *CTHRC1* | 94 | 1.67 | 1.8 | 1.53 | **1.67** |
| *RASL11B* | 94 | 0.4 | 0.41 | 0.45 | **0.42** |
| *STXBP5L* | 94 | 0 | 0 | 0 | **0.00** |
| *M6PR* | 94 | 85.86 | 89.13 | 89.24 | **88.08** |
| *EBF1* | 94 | 0 | 0 | 0 | **0.00** |
| *JADE2* | 94 | 4.72 | 4.79 | 4.27 | **4.59** |
| *TOP2B* | 94 | 76.04 | 95.59 | 88.2 | **86.61** |
| *PPP1R8* | 94 | 35.78 | 37.02 | 35.29 | **36.03** |
| *CD55* | 94 | 27.45 | 31.01 | 31.58 | **30.01** |
| *GABRA5* | 94 | 4.21 | 4.39 | 4.28 | **4.29** |
| *TMEM170A* | 94 | 9.01 | 9.78 | 9.71 | **9.50** |
| *DIDO1* | 93 | 16.03 | 16.6 | 17.23 | **16.62** |
| *PDIA5* | 93 | 57.64 | 57.55 | 57.13 | **57.44** |
| *HIVEP1* | 93 | 10.7 | 12.37 | 12.18 | **11.75** |
| *GNB4* | 93 | 9.58 | 10.98 | 10.53 | **10.36** |
| *KCNA2* | 93 | 0 | 0 | 0 | **0.00** |
| *PRRC2C* | 93 | 24.45 | 29.58 | 30.86 | **28.30** |
| *AKIRIN1* | 93 | 45.33 | 47.33 | 45.25 | **45.97** |
| *SIPA1L2* | 93 | 0.77 | 0.75 | 0.9 | **0.81** |
| *ARHGAP11A* | 93 | 7.61 | 10.17 | 10.28 | **9.35** |
| *MBNL1* | 93 | 35.66 | 43.89 | 40.65 | **40.07** |
| *PHTF2* | 93 | 16.45 | 19.31 | 17.02 | **17.59** |
| *YPEL2* | 93 | 8.23 | 8.84 | 8.2 | **8.42** |
| *GLI2* | 93 | 0.03 | 0.01 | 0 | **0.01** |
| *NEDD1* | 93 | 23 | 25.93 | 26.04 | **24.99** |
| *MORF4L1* | 93 | 152.82 | 163.16 | 158.64 | **158.21** |
| *CDKN1C* | 93 | 0.66 | 0.44 | 0.53 | **0.54** |
| *DOCK4* | 93 | 1.1 | 1.29 | 1.13 | **1.17** |
| *MCTP1* | 93 | 3.89 | 3.88 | 3.87 | **3.88** |
| *PRKAA2* | 93 | 10.13 | 12.12 | 11.09 | **11.11** |
| *RPL29* | 93 | 1060.19 | 1073.55 | 1050.52 | **1061.42** |
| *CXCL3* | 93 | 0.22 | 0.48 | 0.38 | **0.36** |
| *COLEC12* | 93 | 0.74 | 0.84 | 0.89 | **0.82** |
| *PUM2* | 93 | 19.9 | 21.71 | 22.26 | **21.29** |
| *KMT2E* | 93 | 16.02 | 20.32 | 19.48 | **18.61** |
| *GTPBP1* | 93 | 4.93 | 5.91 | 5.93 | **5.59** |
| *FOXF1* | 93 | 0 | 0.01 | 0.03 | **0.01** |
| *AICDA* | 92 | 0.31 | 0.26 | 0.44 | **0.34** |
| *MLLT10* | 92 | 11.67 | 12.14 | 13.26 | **12.36** |
| *RGPD3* | 92 | 0.2 | 0.29 | 0.17 | **0.22** |
| *ZBTB7A* | 92 | 2.23 | 2.63 | 2.66 | **2.51** |
| *FBXO21* | 92 | 33.24 | 35.89 | 33.81 | **34.31** |
| *SEC24D* | 92 | 18.11 | 19.56 | 18.39 | **18.69** |
| *GPR6* | 92 | 0 | 0 | 0 | **0.00** |
| *RAB40B* | 92 | 4.6 | 5.37 | 4.31 | **4.76** |
| *E2F4* | 92 | 14.95 | 16.21 | 16.84 | **16.00** |
| *KHDRBS1* | 92 | 44.31 | 48.45 | 46.51 | **46.42** |
| *GNA13* | 92 | 28.14 | 31 | 29.97 | **29.70** |
| *MRPS21* | 92 | 150.81 | 149.73 | 147.73 | **149.42** |
| *ATXN7* | 92 | 6.6 | 7.96 | 8.03 | **7.53** |
| *GNPTAB* | 92 | 73.65 | 80.8 | 74.76 | **76.40** |
| *ZZEF1* | 92 | 11.33 | 12.98 | 13.12 | **12.48** |
| *SEPT7* | 92 | 69.21 | 80.37 | 76.43 | **75.34** |
| *MNT* | 92 | 0.61 | 0.6 | 0.66 | **0.62** |
| *CAMK2D* | 92 | 22.28 | 24.09 | 23.41 | **23.26** |
| *ZNF268* | 92 | 18.41 | 21.61 | 22.32 | **20.78** |
| *COX10* | 92 | 4.08 | 4.84 | 4.64 | **4.52** |
| *NR3C1* | 92 | 21.47 | 21.15 | 23.28 | **21.97** |
| *TCEANC* | 92 | 1.2 | 1.37 | 1.27 | **1.28** |
| *MITF* | 92 | 62.57 | 68.44 | 65.5 | **65.50** |
| *KLF4* | 92 | 0.62 | 0.71 | 0.67 | **0.67** |
| *CDYL* | 92 | 12.1 | 12.4 | 11.58 | **12.03** |
| *CEMIP2* | 92 | 15.57 | 17.78 | 17.36 | **16.90** |
| *PRR12* | 92 | 0.17 | 0.15 | 0.16 | **0.16** |
| *ELAVL1* | 92 | 11.58 | 11.91 | 11.66 | **11.72** |
| *TMEM248* | 92 | 52.01 | 55.63 | 55.87 | **54.50** |
| *MAP3K2* | 92 | 25.26 | 30.64 | 28.32 | **28.07** |
| *KNTC1* | 92 | 15.01 | 17.25 | 17.23 | **16.50** |
| *C1QL1* | 91 | 1.97 | 1.65 | 1.98 | **1.87** |
| *FAM76B* | 91 | 10 | 13.92 | 12.26 | **12.06** |
| *NLK* | 91 | 7.48 | 8.45 | 8.08 | **8.00** |
| *ABTB2* | 91 | 0.26 | 0.29 | 0.4 | **0.32** |
| *KMT5B* | 91 | 15.72 | 18.14 | 17.95 | **17.27** |
| *ANK3* | 91 | 7.88 | 7.44 | 6.9 | **7.41** |
| *E2F3* | 91 | 7.41 | 8.7 | 8.58 | **8.23** |
| *GNAI1* | 91 | 85.77 | 93.42 | 84.63 | **87.94** |
| *LMBR1* | 91 | 18.25 | 19.96 | 19.12 | **19.11** |
| *LZTS2* | 91 | 15.64 | 16.73 | 16.41 | **16.26** |
| *SHOC2* | 91 | 22.56 | 26.7 | 23.46 | **24.24** |
| *PAK1IP1* | 91 | 27.1 | 27.53 | 28.59 | **27.74** |
| *IL17RD* | 91 | 2.88 | 3.34 | 3.1 | **3.11** |
| *ASH1L* | 91 | 14.27 | 17.93 | 17.45 | **16.55** |
| *VRK1* | 91 | 21.83 | 24.66 | 24.65 | **23.71** |
| *RSL24D1* | 91 | 143.1 | 158.33 | 146.55 | **149.33** |
| *TCF21* | 91 | 0 | 0 | 0 | **0.00** |
| *LMTK2* | 91 | 4.65 | 5.41 | 5.4 | **5.15** |
| *BACH2* | 91 | 1.29 | 1.55 | 1.36 | **1.40** |
| *ADNP* | 91 | 12.82 | 14.16 | 15.27 | **14.08** |
| *SMURF1* | 91 | 7.8 | 9.56 | 8.83 | **8.73** |
| *THBD* | 91 | 0.58 | 0.49 | 0.82 | **0.63** |
| *FMNL3* | 91 | 1.79 | 2.08 | 1.98 | **1.95** |
| *PAN3* | 91 | 12.11 | 14.29 | 12.44 | **12.95** |
| *ALX1* | 91 | 0.06 | 0.03 | 0 | **0.03** |
| *ABCC5* | 91 | 18.25 | 21.28 | 20.43 | **19.99** |
| *GBX2* | 91 | 0.16 | 0.29 | 0.29 | **0.25** |
| *MT1X* | 90 | 11.22 | 12.58 | 13.02 | **12.27** |
| *HOXB7* | 90 | 0.03 | 0 | 0 | **0.01** |
| *CYFIP2* | 90 | 26.46 | 28.3 | 28.55 | **27.77** |
| *FAM83B* | 90 | 4.95 | 5.66 | 4.71 | **5.11** |
| *NECAB1* | 90 | 0.01 | 0.05 | 0 | **0.02** |
| *ICE2* | 90 | 19.87 | 23.06 | 21.09 | **21.34** |
| *CXCR4* | 90 | 12.85 | 13.38 | 13.67 | **13.30** |
| *DCLK2* | 90 | 1.52 | 1.9 | 1.68 | **1.70** |
| *SIX1* | 90 | 0.11 | 0.12 | 0.18 | **0.14** |
| *GJA1* | 90 | 84.85 | 90.45 | 83.19 | **86.16** |
| *SCYL1* | 90 | 17.58 | 18.41 | 19.31 | **18.43** |
| *SH2B3* | 90 | 2.19 | 2.5 | 2.52 | **2.40** |
| *BCL7A* | 90 | 4.33 | 4.68 | 4.78 | **4.60** |
| *CUX1* | 90 | 15.98 | 15.28 | 15.42 | **15.56** |
| *RIMS2* | 90 | 1.31 | 1.65 | 1.51 | **1.49** |
| *MRPL49* | 90 | 31.01 | 30.16 | 30.91 | **30.69** |
| *PEX3* | 90 | 13.9 | 14.83 | 12.03 | **13.59** |
| *SLC10A7* | 90 | 2.5 | 3.07 | 3.06 | **2.88** |
| *PIAS3* | 90 | 3.91 | 4.04 | 3.55 | **3.83** |
| *FUBP1* | 90 | 82.02 | 84.76 | 81.02 | **82.60** |
| *WARS2* | 90 | 14.94 | 16.35 | 13.96 | **15.08** |
| *CCL2* | 90 | 46.63 | 49.67 | 54.6 | **50.30** |
| *GCC2* | 90 | 20.68 | 29.25 | 23.95 | **24.63** |
| *ZNF704* | 90 | 0.08 | 0.08 | 0.05 | **0.07** |
| *DNM1L* | 90 | 31.18 | 36.57 | 34.61 | **34.12** |
| *AKAP13* | 90 | 16.33 | 16.72 | 16.49 | **16.51** |
| *CACNB4* | 90 | 0.19 | 0.29 | 0.2 | **0.23** |
| *CUL2* | 90 | 22.54 | 27.51 | 26.6 | **25.55** |
| *SCAF8* | 90 | 21.85 | 23.58 | 23.1 | **22.84** |
| *LMNB2* | 90 | 12.55 | 13.86 | 13.29 | **13.23** |
| *ELOVL5* | 90 | 68.83 | 76.01 | 71.8 | **72.21** |
| *NPAS3* | 90 | 0.23 | 0.32 | 0.28 | **0.28** |
| *RASSF3* | 90 | 20.62 | 21.73 | 21.84 | **21.40** |
| *HS6ST2* | 90 | 4.29 | 5.29 | 5.13 | **4.90** |
| *MYBL1* | 90 | 6.07 | 7.67 | 7.19 | **6.98** |
| *POF1B* | 90 | 0 | 0 | 0 | **0.00** |
| **MIR99A** | | | | | |
| **miR-99a-5p** | | | | | |
| *TRIB2* | 96 | 3.82 | 4.46 | 4.51 | **4.26** |
| *KBTBD8* | 94 | 2.47 | 3.28 | 2.95 | **2.90** |
| *SMARCA5* | 94 | 38.66 | 44.73 | 41.85 | **41.75** |
| *MTOR* | 93 | 16.88 | 17.71 | 18.73 | **17.77** |
| *HS3ST2* | 91 | 0 | 0 | 0 | **0.00** |
| *RAVER2* | 91 | 4.75 | 5.53 | 5.37 | **5.22** |
| *ZZEF1* | 90 | 11.33 | 12.98 | 13.12 | **12.48** |
| *BAZ2A* | 90 | 4.47 | 5.56 | 5.26 | **5.10** |
| **miR-99a-3p** | | | | | |
| *RSBN1L* | 93 | 8.7 | 10.24 | 9.34 | **9.43** |
